# Guides for Developing Hundreds of Novel Chiral MXenes and MBenes Nanosheets/Quantum Dots for Next‐Generation Chiral Engineered Biomaterials Applications

**DOI:** 10.1002/adhm.202502422

**Published:** 2025-09-04

**Authors:** Alireza Rafieerad, Ahmad Amiri

**Affiliations:** ^1^ Institute for Molecular Biosciences Johann Wolfgang Goethe Universität 60438 Frankfurt am Main Germany; ^2^ Institute for Biology and Biotechnology of Plants University of Münster Schlossplatz 8 48143 Münster Germany; ^3^ Regenerative Medicine Program Institute of Cardiovascular Sciences St. Boniface Hospital Research Centre Department of Physiology and Pathophysiology Rady Faculty of Health Sciences University of Manitoba Winnipeg Canada; ^4^ Russell School of Chemical Engineering The University of Tulsa Tulsa OK 74104 USA; ^5^ Department of Mechanical Engineering University of Tulsa Tulsa OK 74104 USA

**Keywords:** bio‐related applications, enhanced colloidal properties, l/d‐handed biomaterials, periodic chiral MXenes/MBenes, prediction/optimization, prospective modification vacancies

## Abstract

The development and multiple bio‐applications of chiral MXene nanosheets and derived quantum dots‐based heterostructures as next‐generation plant biostimulants are recently reported in **Small** for the first time. This chirality‐induction came at a critical juncture in the field, as the safety efficacy of synthetic low‐dimensional materials, including MXenes, challenges their clinical, agricultural, and environmental translatability. Using a rational surface engineering and structural‐modification strategy, distinct left‐ or right‐handed chiral MXenes are developed. These tailored asymmetric MXenes inherently leverage desirable durability, long‐term biocompatibility, and multifunctional bioactivities. Chirality, as a natural biological specification of living organisms and most bio‐macromolecules, plays a pivotal role in their cellular functions, interactions, enzyme–substrate recognition, protein folding, genetic encoding, and immune‐related mechanisms. Chiral engineering of nanomaterials has set paradigms to open up avenues of studies toward designing numerous novel chiral MXenes and MBenes. Here, this innovative perspective presents a roadmap for periodic development of chiral MXenes/MBenes of diverse chemical compositions and forms. In particular, prospective vacancies and step‐by‐step guides for constructing hundreds of different MXene/MBene formulations are proposed through diverse chiral‐active sources. Induced chirality is anticipated to relatively enhance the properties and biocompatibility of their original materials. It paves the way for extending and optimizing this nano‐biotechnology to more effectively activate, regulate, or control biological responses. These prospects are anticipated to cover antimicrobial coating, immune modulation, tissue engineering, drug delivery, cancer treatments, cell therapies, organ‐transplant rejection prevention, and other healthcare/diagnostic aspects of electrochemically active‐nanomaterials in bio‐tracking, wearable bioelectronics, and biomedical sensors. The basic predictions of their toxicity behaviors across various biological settings are also discussed, and their bio‐properties are speculated on to prioritize design recommendations and strategize the possible chiral‐functionalization challenges.

## Graphical abstract

The emerging field of MXene/MBene materials has progressed rapidly, advancing diverse research fields, including biomedical engineering, biomedicine, agriculture, and the environment. This nanobiotechnology can tackle longstanding challenges in these areas. However, stability, long‐term biocompatibility, and environmental safety are key for their practical applications. A novel and rational strategy is recently reported to enhance these properties by inducing stable chirality in MXene nanosheets and derived quantum dots. This approach has set paradigms for developing chiral MXene/MBene biomaterials with asymmetric left‐ or right‐handed structures. It may support the modeling and construction of hundreds of MXenes/MBenes. This innovative perspective presents the idea, guide, and step‐by‐step chemical reactions, paving the way for designing new research on applications of next‐generation chiral‐engineered MXenes/MBenes.

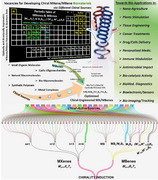

## 1 Introduction

Chirality, or chiral recognition, is one of the most fundamental and crucial attributes of nature, living organisms, most bio‐macromolecules, and various natural systems.^[^ ^1^, ^2^ ^]^ In particular, the DNA structures, amino acids, proteins, lipids, carbohydrates, and most cell metabolites are intrinsically chiral or have left/right‐handed chirality in their biochemical structures.^[^ ^3^, ^4^ ^]^ Chirality is characteristic of objects that are non‐superimposable on their mirror images. It plays a key role in the mechanisms and interactions of biological molecules.^[^ ^5^ ^]^ For instance, cells and their DNA contents can naturally sense chirality and chiral‐induced characteristics. This ability to readily interact and communicate with enantiomorphous surfaces positively impacts various cellular interactions, adhesion, and activation behaviors. Thus, chirality induction into the structure of low‐dimensional materials has enabled chiral engineering vacancies for various applications in diverse bio‐related fields.

Chiral‐active substances and chirality‐induced materials have significantly impacted various sectors of basic sciences and research and development for decades. These natural or synthetic materials offer notable advantages in catalysis and electrochemical applications,^[^ ^6^ ^]^ and have become increasingly important in diverse bio‐fields, including biomedical engineering, immune regulation, and nanomedicine^[^ ^7^, ^8^, ^9^, ^10^, ^11^, ^12^, ^13^, ^14^, ^15^, ^16^, ^17^, ^18^, ^19^ ^]^ and agriculture.^[^ ^20^ ^]^ Indeed, the emergence of these technologies has created new possibilities for healthcare and therapeutic advancements, drug developments, biomedical diagnostics, sensors, the food industry, and agrochemical production.

Recently, there has been a tremendous interest in designing and developing low‐dimensional chiral‐active biomaterials. They can potentially offer enhanced biocompatibility and bioactivity over their similar non‐chiral structures to more effectively address the longstanding challenges in bio‐related or environmental fields. Chiral‐tuned properties may also contribute to reduce the safety risks associated with probable nano‐toxicity concerns of engineered nanomaterials, especially at higher doses and longer exposure. Induction of chirality into the surface or structure of nano‐/quantum‐sized biomaterials improves their biocompatibility and bio‐functional properties beyond those of their original forms. Progress includes chiral inorganic nanomaterials—such as metals, metal oxides, inorganic semiconductors, and composites—and organic nanomaterials, like molecule‐based monomers and polymers, porous organic frameworks or tree‐like branched units, and supramolecular and carbon‐based nanostructures. Furthermore, chiral‐modified hybrid and composite materials may offer synergistic effects to enhance their multifunctional properties. This is especially the case when single materials cannot provide all the specific requirements of the targeted applications.

From biological viewpoints, the need to avoid unnecessary chemical complexities on bioactive nanomaterials has highlighted the value of carbon‐based structures. They are appreciated for their sustainability, natural abundance, biocompatibility, and tunable physicochemical properties. This fast‐growing class of low‐dimensional biomaterials is a nascent area of the field, showing promise for bio‐related applications. MXene is the latest‐discovered, largest, and most tunable family of carbon‐based nanomaterials.^[^ ^16^, ^17^ ^]^ Based on a “Google Scholar Citations” topic search in 2025, there have been over 21 000 publications with “MXene” in the title. Emerging transition metal carbides, nitrides, and/or carbonitrides (MXenes) have a general formal of M_n+1_X_n_T_x_, where “M” stands for one or multiple transition metals in the periodic table, “A” is carbon and/or nitrogen, and “T*x*” represents surface functional terminations like ─OH, ─F, ─O, ─Cl along with the possibility of expanding to further surface functional groups such as carboxyl, amine, etc. Over the recent years, the technology of MXenes has profoundly progressed and advanced many research fields, including but not limited to energy storage/transfer, electronics and optoelectronics, biomedicine, biomedical engineering, sensors, nano‐agriculture, and the environment.^[^ ^17^, ^21^, ^22^, ^23^, ^24^, ^25^, ^26^, ^27^, ^28^, ^29^, ^30^ ^]^

More recently, research interests have grown on potential applications of bioactive MXenes to improve the proposed nanomedicine technologies for treating cancer and degenerative diseases, developing efficient neurochemical sensors, and improving translational therapies by modulating immune responses to control inflammation and prevent cell/organ transplant rejection. Indeed, the technology of MXenes has revolutionized these fields, acting as next‐generation multifunctional nano‐bioactive agents with programmable surface and properties. The field of MXene biomaterials remains dynamic, with lots of room for advancing their technology for optimized productions, microstructural stability, reproducibility enhancements, and large‐scale cost reductions to reach their maximum potential. The promising future of MXene is anticipated once these nanomaterials have been proven sufficiently safe for translation/implementation in clinics, agriculture, and the environment. Thus, designing, developing, and optimizing highly biocompatible chiral MXenes with enhanced bio‐properties can significantly extend the boundaries of these technologies to their higher levels to improve efficacy and potentially help bring them closer to real‐world applications.

More recently, the theoretical or experimental development of a new derivation of the MXene family known as “MBene” has been reported.^[^ ^31^, ^32^, ^33^, ^34^ ^]^ Striking similarities in structure and properties of transition metal borides (MBenes) with MXenes highlight the potential of this new class of low‐dimensional materials for various bio‐related applications. To date, in comparison to MXenes, a lower frequency of MBenes has been reported in the literature. However, this particular field is also growing fast in terms of synthesis and applications. For instance, MBenes have been shown to have potential for electronic and optoelectronic properties, suggesting their potential properties to be used for next‐generation bio‐sensors and wearable/medical electronics. Further, MBenes have been reported to possess high structural and physico–mechanical properties, highlighting their suitability for those applications that require more durability and long‐term stability of these bioactive and electroactive‐functional materials. Thus, it is anticipated that chiral engineering of MBenes would also effectively enhance their biocompatibility and bioactivity properties competitively to chiral MXenes.

Considering these accounts, from the current perspective, we present a formulated and classified roadmap for designing and constructing new chiral MXenes/MBenes with periodic compositional diversities and surface modification vacancies using different chiral sources. Hence, we proposed the guides and step‐by‐step chemical reactions for inducing stable chirality in each MXene/MBene, describing how the left‐/right‐handed chirality induction can improve their colloidal dispersibility, durability, and functional bio‐properties. We also provided insightful discussions for alternative chirality induction strategies as potential methods to form chiral‐active heterogeneous nanosheets, quantum dots, and mixed‐dimensional heterostructures. Lastly, the basic principles are suggested for the design of experiments, optimization, and bio‐properties predictions of chiral MXenes and MBenes based on the mathematical, machine learning, artificial intelligence (AI)‐based, and chemistry‐aided toxicity prediction methods. This optimization may involve both the synthesis parameters and chirality inductions. Initial risk factor management and design prioritization are also recommended.

## 2 An Overview on the Reported Biocompatibility/Toxicity of MXenes/MBenes with Bio‐Systems

As pointed out in the previous section, the majority of as‐synthesized MXenes and MBenes have shown high biocompatibility with biological systems at controlled doses and conditions. Numerous publications have assessed these biocompatibility properties using in vitro, ex vivo, in vivo, *in‐seedling*, and *in‐planta* models. This extensive literature suggests MXenes/MBenes are relatively biocompatible within the tested durations, suggesting biocompatibility thresholds for that specific model. However, some of these studies reported that increasing the dose or treatment duration could lead to an increased risk of nanotoxicity symptoms emerging. Thus, the field has critically considered this aspect worldwide to enhance the long‐term biocompatibility and safety efficacy of today's nanomaterials, which is crucial for their practical applications. Some of the literature on biocompatibility or potential toxicity of MXenes/MBenes with different types of bio‐systems, cancer cells, and pathogens is adapted and represented in Figures S1 to S4 (Supporting Information).^[^ ^35^, ^36^, ^37^ ^]^ These data highlight the inherent biocompatibility properties of these materials at controlled doses, which have been shown to be dependent on relative composition and form. These analyses emphasize the urgent need for optimization and improvement of long‐term biocompatibility. The anticipated advancements can significantly progress the field and bring its technology closer to practical bio‐applications.

More importantly, as research and production of MXene/MBene nanomaterials grow, so does their inevitable release and exposure to biological systems, the environment, and ecosystems. Thus, a clear understanding and careful evaluation of their optimal working doses and nano‐toxicities are critical for biocompatibility optimizations and minimizing probable adverse effects. Given the expansion of MXenes/MBenes for multiple applications in biomedical and agricultural fields, it is essential to adequately understand their interactions within different physiological systems for safe and effective use in bio‐medicine, advanced therapeutics/biosensing, and agricultural protection. Therefore, assessing and improving the biocompatibility and safety of MXenes/MBenes to their maximum possible levels is a central aim in advancing their application from research to real‐world scenarios.

This central concern regarding the nanotoxicity of MXenes/MBenes involves two key aspects. First, their small size and large surface area result in stronger interactions with biological systems than conventional biomaterials. Second, their unique surface groups and negative charge make them highly reactive with organic and inorganic compounds/materials. These inherent reactivity and bioactive properties are shown to be highly beneficial in making them multifunctional biomaterials. However, if these specifications are not controlled, they may cause unintended biological effects. Indeed, when nanomaterials, such as MXenes/MBenes, contact cells—whether healthy, cancerous, or pathogenic—they can be internalized or adhere to membranes. This can be programmed to smartly activate the targeted bioactivity by choosing an appropriate composition, form, and dose for that application. On the other hand, they may adversely disrupt cellular functions and trigger responses such as apoptosis and pyroptosis. Indeed, their high or uncontrolled doses may cause cytotoxicity in vitro and in vivo can accumulate in organs, altering biological mechanisms. To date, extensive reports have evaluated the short‐ and mid‐term biocompatibility of MXenes/MBenes; however, few studies have addressed their long‐term safety. It further highlights the need for more research to ensure their safety. Chiral engineering of bioactive nanomaterials is a promising strategy to enhance their biocompatibility and bioactivity behaviors. We previously introduced this strategy for MXene. Here, the proposed chirality induction is described in detail for new MXenes/MBenes.

## 3 Vacancies for Chiral Engineering of MXene/MBene‐Based Biomaterials

### 3.1 Amino Acid‐Based Chirality Induction in MXene/MBene Nanosheets and Derived Quantum Dots by EDC/NHS Crosslinking Method

**Figure 1:**
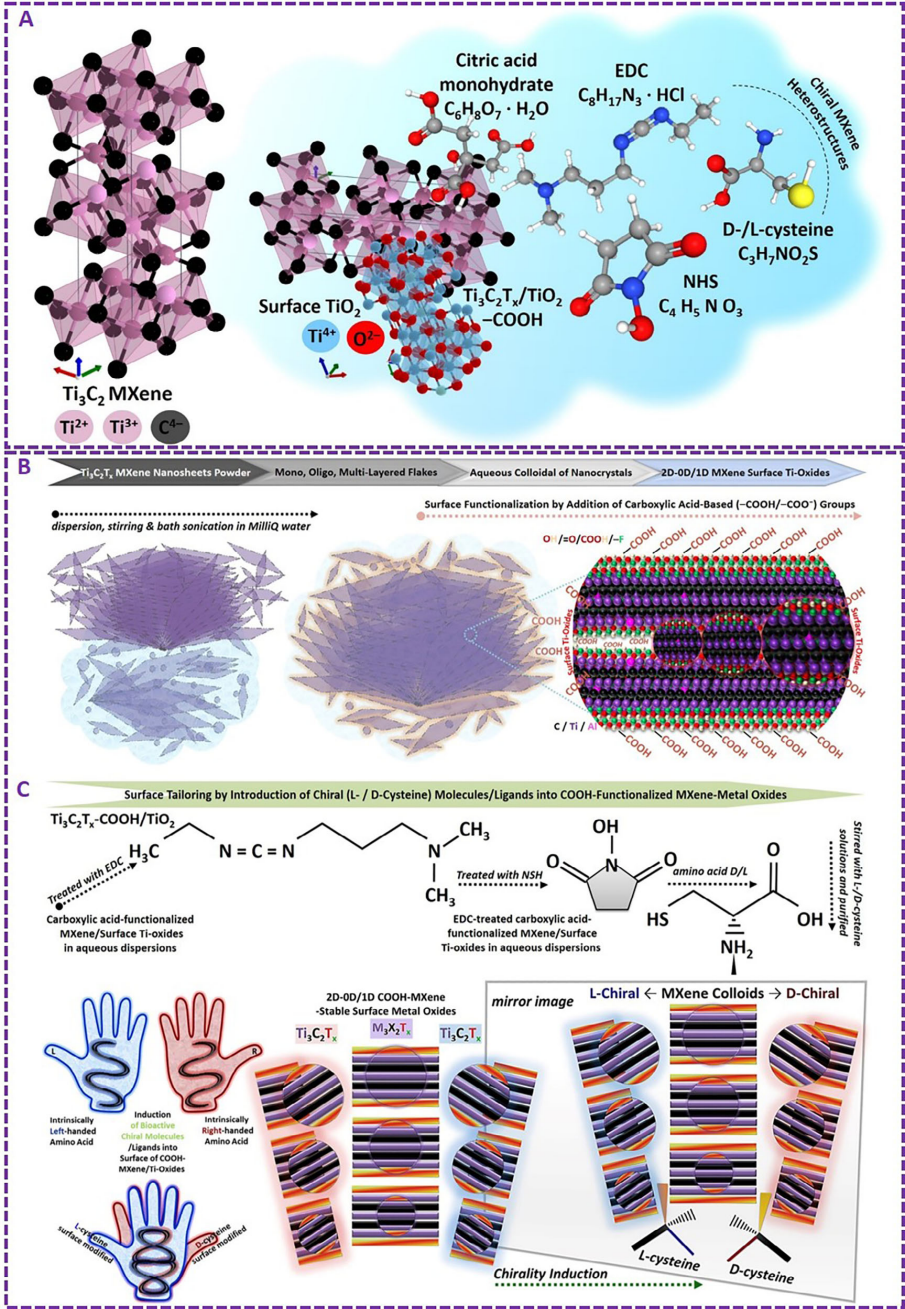
Representation of the schematic surface oxidation and hydrolysis of Ti_3_C_2_T_x_ MXene nanosheets aqueous dispersion, carboxyl‐functionalization, and chirality induction via EDC/NHS crosslinking. A–C) The applied EDC/NHS (C_8_H_17_N_3_ · HCl / C_4_H_5_NO_3_) method could effectively induce chirality into the surface of these MXene nanosheets and their derived quantum dots‐based mixed‐dimensional heterostructures. Chiral engineering has been performed to induce left‐/right‐handed asymmetry in the structure of carboxyl‐functionalized Ti_3_C_2_T_x_ in aqueous dispersions. The used three‐dimensional (3D) crystal structures of Ti_3_C_2_ (Hexagonal mp‐1094034, *P6_3/mmc*, 194) and corresponding surface titanium dioxide (TiO_2_) metal oxide (Tetragonal, mp‐390, *I4_1/amd*, 141) were sourced from the “Materials Project” database open webpage (https://materialsproject.org) with referred to their publications in the original paper of adaption^[^ ^1^ ^]^ (under user Creative Commons Attribution 4.0‐license) The models of citric acid monohydrate, EDC/NHS, and left‐/right‐handed cysteine were obtained/sources from “PubChem open database website” with reference to their publications and citing policy. PMID: 39558165 PMCID: PMC11701573. PubChem, Bethesda (MD): National Library of Medicine (US), National Center for Biotechnology Information; 2004‐. PubChem Compound Summary for CID 92851, d‐cysteine: https://pubchem.ncbi.nlm.nih.gov/compound/D‐cysteine. PubChem, Bethesda (MD), Cysteine; 2025 https://pubchem.ncbi.nlm.nih.gov/compound/Cysteine). Panels are reproduced and merged with permission from our previous work, Copyright, CC Open Access Attribution 4.0 International, Small, Wiley).^[^ ^1^ ^]^

The induction of stable chirality in the surface of MXene/MBene sheets, quantum dots, and derived heterostructures by the EDC/NHS (1‐ethyl‐3‐(3‐dimethylaminopropyl)carbodiimide)/N‐hydroxysuccinimide, C_8_H_17_N_3_ · HCl/C_4_H_5_NO_3_) method has been initially presented and described in our recent work.^[^ ^1^ ^]^ The used EDC/NHS is an established universal two‐step chemical coupling (crosslinking) process, which can be efficiently used to create stable amide‐based bonds between molecules of the treated materials, specifically between their carboxyl‐ and amine‐based surface groups. It involves using EDC to activate carboxyl (e.g., –COOH) terminations followed by the NHS contribution to enhancing the reaction by effectively creating more stable intermediates. The workflow of the induced chirality in MXene using different chiral‐active amino acid ligands is represented in **Figure** **1** (for Ti_3_C_2_T_x_ as the most common representative of the MXene family).

Moreover, the aqueous colloidal dispersions of these chiral‐engineered mixed‐dimensional transition‐metal carbide/oxide heterostructures could be efficiently customized into other material forms, including coated thin films, dried powder flakes, and vacuum‐filtered membranes. They are also compatible with incorporating into different 3D bio‐polymers as bioactive additive agents. This facile method of converting the symmetric two‐dimensional (2D) MXenes to their asymmetric chiral‐active MXene nanosheets and quantum dots in colloidal dispersion systems has been shown to be highly efficient in forming highly stable chemical bindings, which can remain unchanged (or without any significant microstructural changes and oxidative degradation/decomposition) for several months of storage in water‐based media at around four degrees.

**Figure 2:**
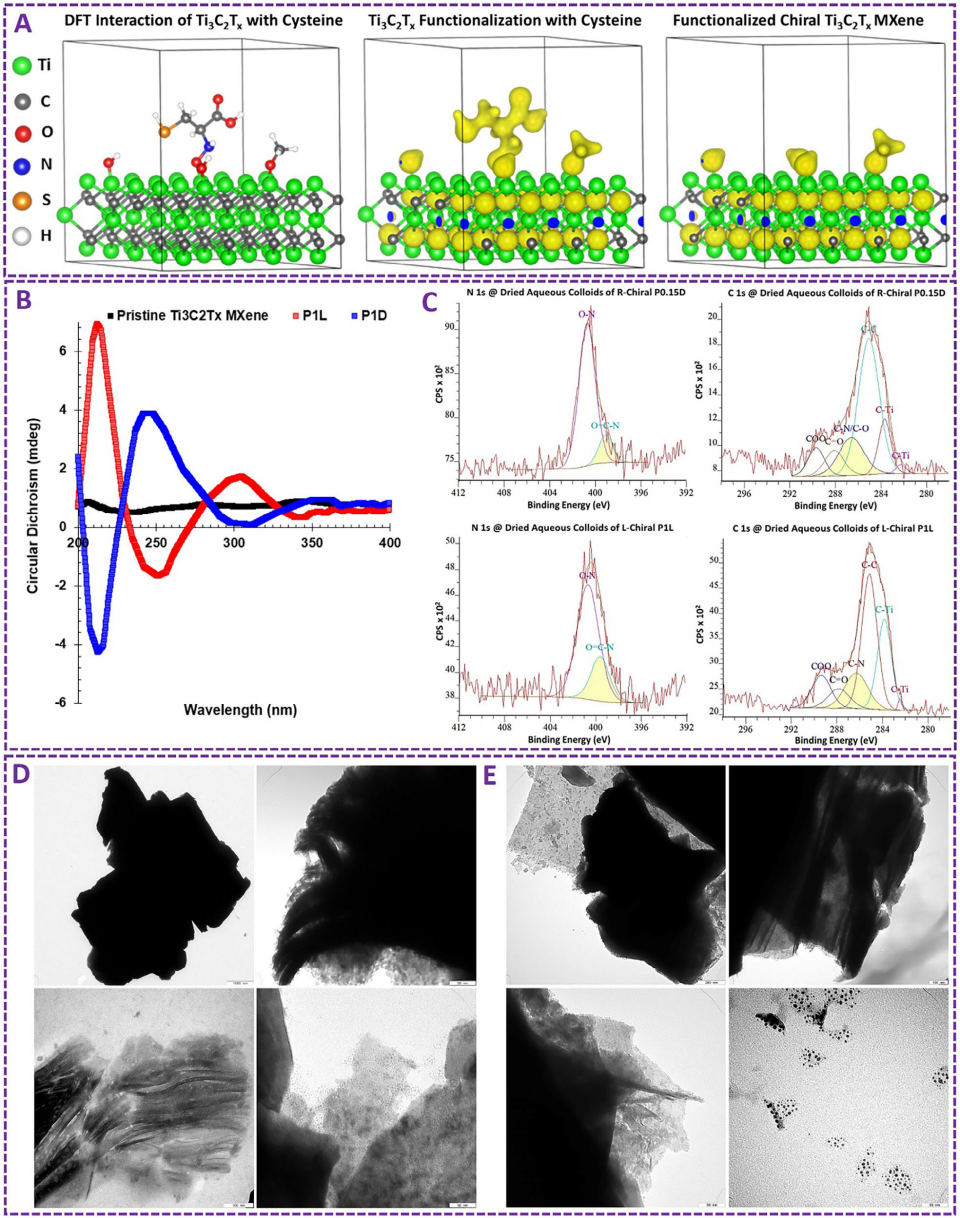
A) The DFT calculations of Ti_3_C_2_T_x_ MXene nanosheets and chiral‐engineered MXene aqueous colloids. It represents the atomic structure of cysteine amino acid molecules adsorbed on the surface of as‐functionalized MXene nanosheets. This DFT data represents the functionalization of Ti_3_C_2_Tx by cysteine ligands. B) Circular dichroism (CD) spectroscopy of functionalized right‐/left‐handed chiral‐engineered MXenes and pristine MXene aqueous dispersions. C) It represents the X‐ray photoelectron spectroscopy (XPS) narrow scan fitting analysis of these chiral‐induced MXenes. D,F) Transmission electron microscopy (TEM) images represent morphological characterization of these chiral MXenes. Their mixed‐low‐dimensional microstructures include Ti_3_C_2_T_x_ nanosheets, zero‐dimensional (0D) MXene quantum dots, and stable surface titanium oxide particles in the form of heterostructured clusters. The panels are reproduced/merged with permission from our previous work, Copyright, CC Open Access Attribution 4.0 International, Small, Wiley.^[^ ^1^ ^]^

Chirality induction in MXenes through the versatile EDC/NHS crosslinking method enables several advantages, including ease of application, relatively fast reaction, and typical laboratory equipment without the necessity of complex devices or expensive technologies. It has been proven effective in introducing both left‐ and right‐handed optically active asymmetric chirality into the structure of MXene end‐products. This surface tailoring and structural manipulation strategy has also shown the capacity and rational specificity for enhancing bio‐properties of MXene types for targeted bio‐applications. These principles have been further validated by density functional theory (DFT) and different physicochemical characterizations of tuned MXenes (see **Figure** **2**). Our data elucidated the efficient interaction and molecular binding at cysteine ligand‐Ti_3_C_2_T_x_/titanium oxide interfaces both theoretically and experimentally.

**Figure 3:**
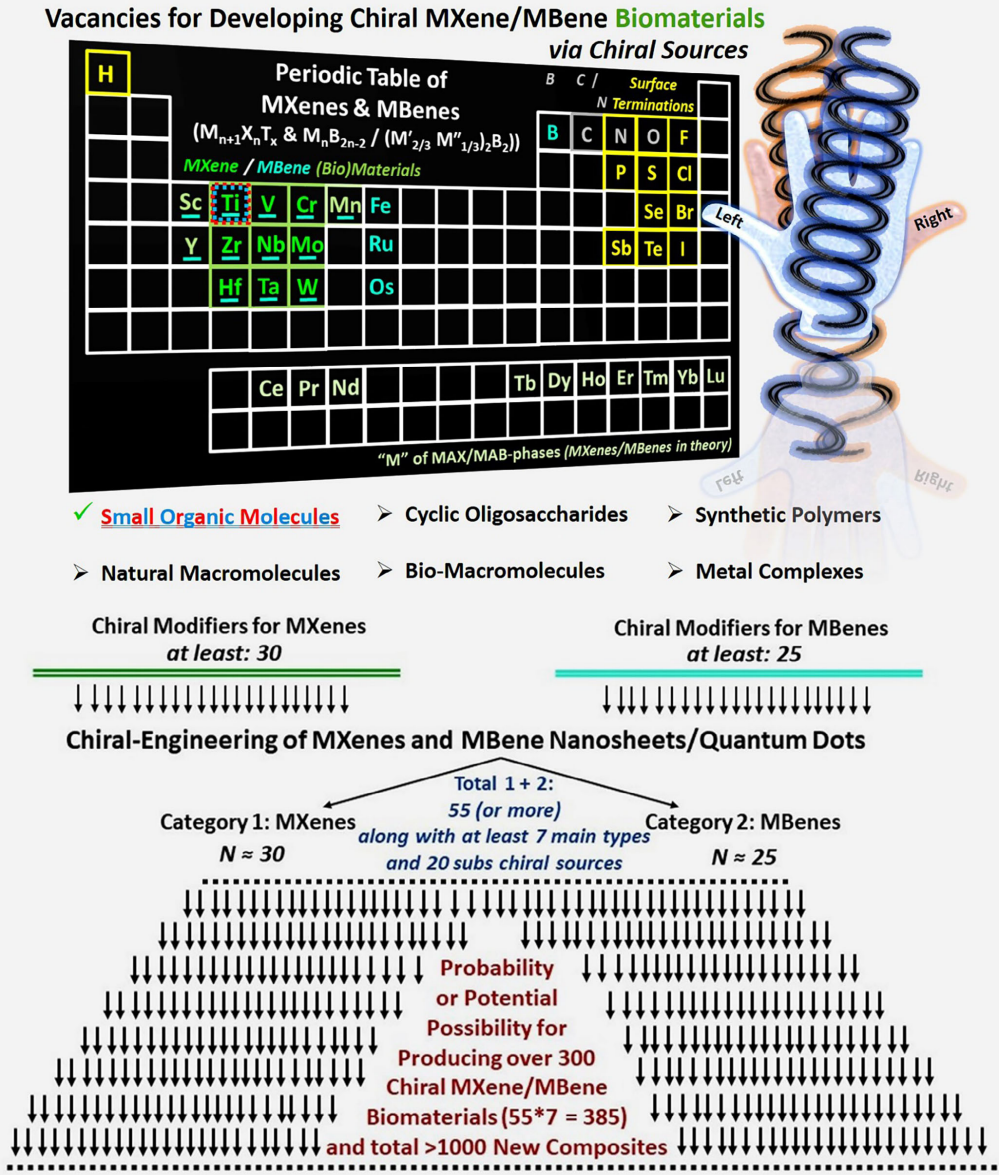
The proposed periodic table of chiral MXenes and MBenes and vacancies for engineering different asymmetric chiral‐engineered biomaterials. This innovative perspective presents the rational for developing hundreds of novel MXenes and MBenes (nanosheets/quantum dots/heterostructures) with different chemical compositions, experimentally or by computation. Considering the reported MXene compositions (at least 30) and MBenes (at least 25), and the availability of various chiral active sources (at least seven main types and over 20 sub‐types), it enables the possibility of constructing numerous chiral MXenes/MBenes with potentially enhanced properties for diverse bio‐applications.

**Figure 4:**
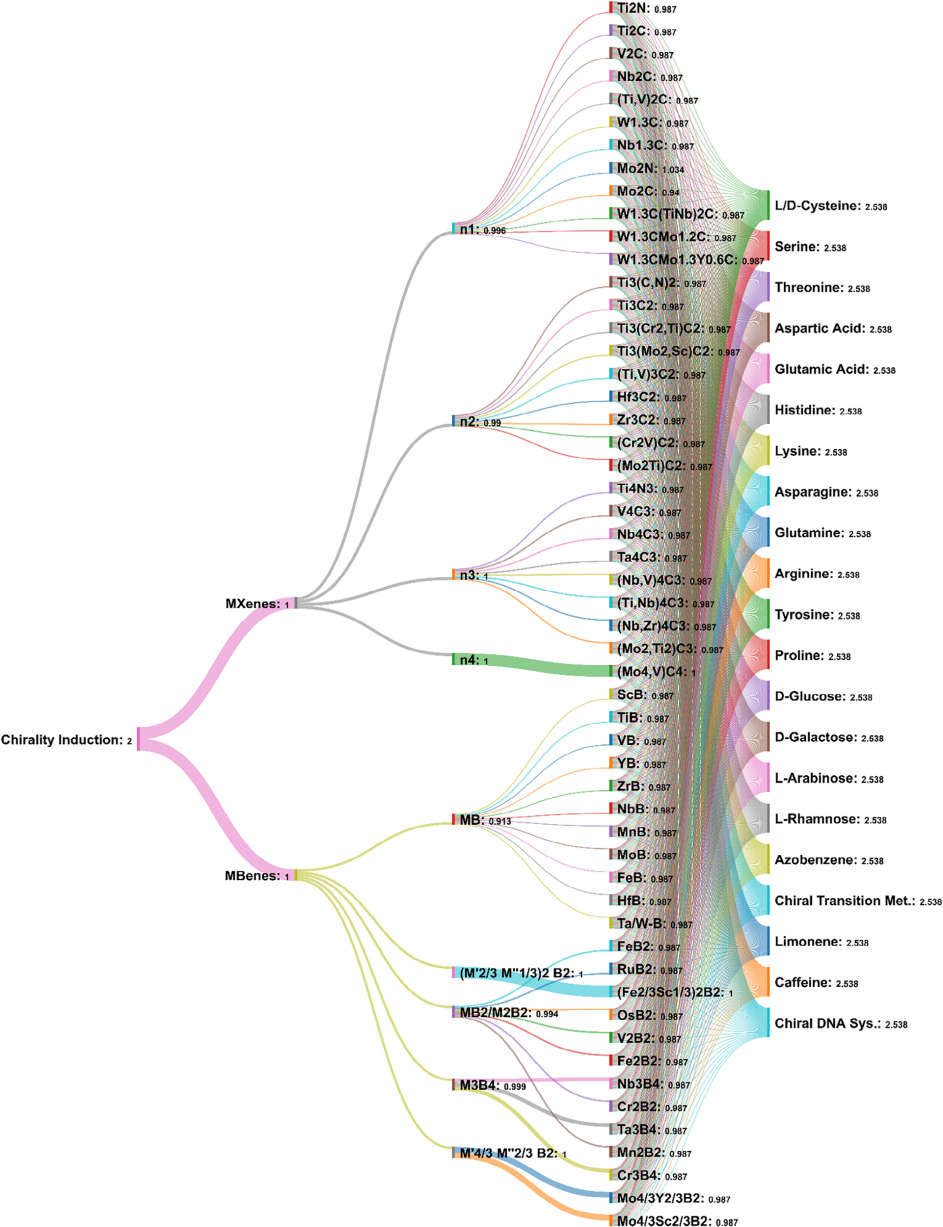
A representative illustration of MXenes and MBenes of different chemical compositions toward developing new chiral nanosheets/quantum dots biomaterials. Generally speaking, so far, it has been reported that at least 30 MXene compositions are feasible to be synthesized. Besides, over 20 (e.g., 25) different chemical formulations of MBenes have been proposed. Further, around seven or more distinct chiral sources are available. Thus, it is anticipated that 30+25*7=385 chiral MXenes/MBenes can be theoretically or experimentally prepared (55*∼20 distinct chiral sources: over 1000 possibilities of chiral‐engineering functionalization, in case of considering each single chiral agent in the proposed tree diagram). This periodic chiral table is extendable by discovering new compositions of MXenes and MBenes, or their combinations. This tree diagram is created by giving equal probability to each sample for fair presentation (total frequency of parent to new nodes, summation of all components’ probability in cluster ≈1.00). We designed this diagram only for visualization on the possibility of developing of hundreds of novel MXenes/MBenes, and their probability values are considered equally. 1199 flows over between 87 nodes are inserted. It was made at the SankeyMATIC Open Diagram web tool created by Steve Bogart (https://sankeymatic.com/).

It is important to note that the applied novel chiral conjugation strategy for MXenes holds great potential capacity to induce chirality into the surface/structure of other akin vacancies of MBenes. **Figures** **3** and **4** display the proposed diagrams for periodic development of several chiral MXene and MBene biomaterials. It is anticipated that the applied method for chiral engineering of MXenes can be also potentially applied to other compositions of this family and be effective in developing other chemical compositions of chiral MXenes. However, the method's feasibility, efficacy, and properties of the obtained chiral biomaterials need careful evaluation for future product development, relative to the model presented for the Ti_3_C_2_T_x_ MXene prototype.

The proposed periodic table and its tree‐like diagram show the chemical diversity capacity of MXene/MBene compositions. Distinct amino acids or other natural/synthetic chiral‐active sources can be potentially used for transferring the stable chirality in these compositions, converting them efficiently from symmetric to asymmetric structures. it may then enable the production of hundreds of chiral‐engineered MXenes/MBenes. In particular, a wide range of amino acid ligands (mostly with l‐ or d‐handed enantiomers) can act as natural or commercial ligands for transferring stable chirality to MXenes and MBenes. The proposed chiral‐engineered products are expected to benefit from high levels of long‐term biocompatibility, enhanced water solubility, and functional bio‐properties compared to their original materials. The method processability and chemical diversity of MXene/MBene formulations (single, hybrid, and/or multiphase “M” subsites) can provide the possibility of developing numerous chiral biomaterials with modified stability and properties.

**Figure 5:**
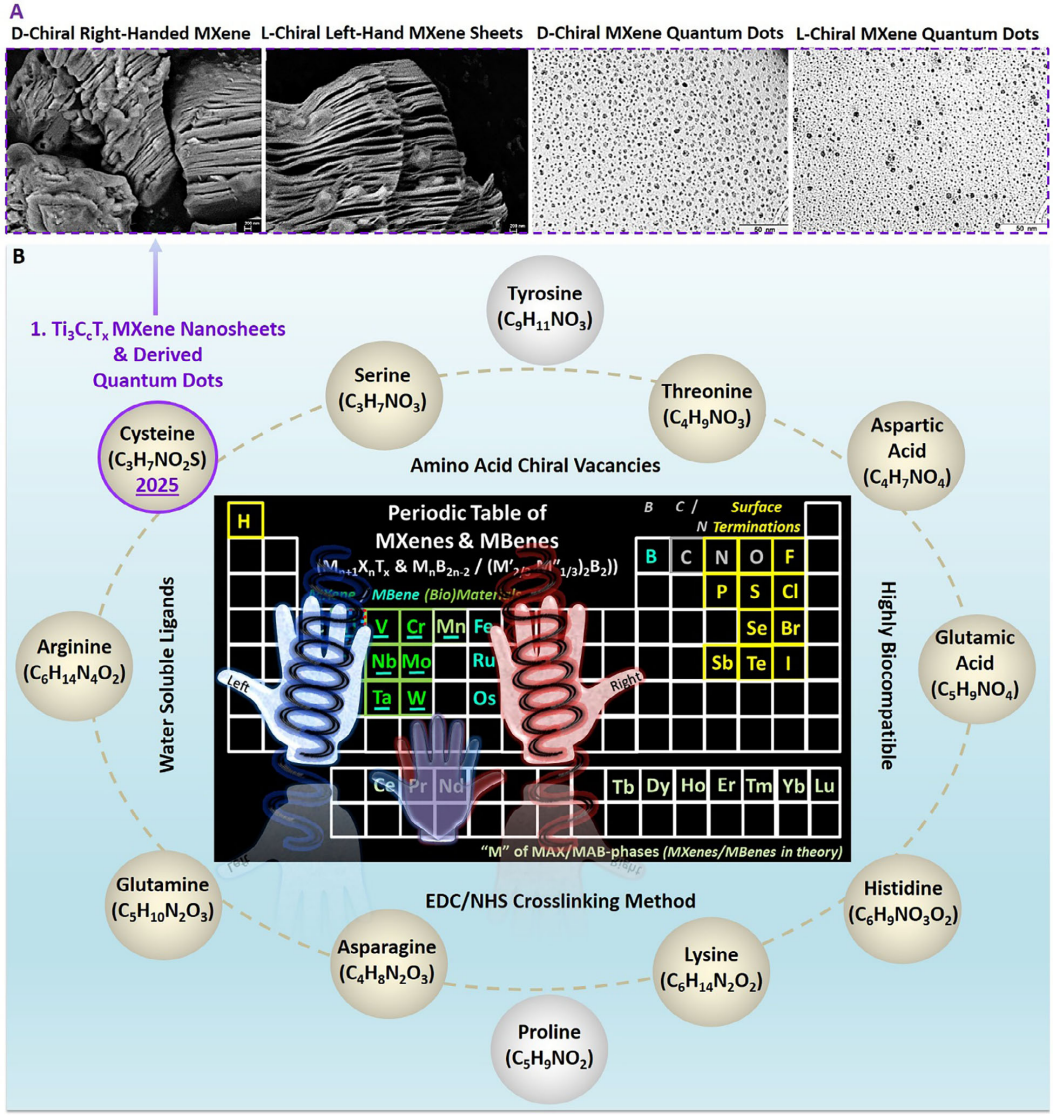
A perspective for designing the periodic table of chiral MXenes and proposed amino acid‐based strategies for inducing chirality. A) The SEM morphology of fabricated d‐/l‐chiral Ti_3_C_2_T_x_ MXene nanosheets and drive quantum dots. B) Vacancies for synthesizing new chiral MXenes and heterostructures. Panel “A” is reproduced/merged with permission from our previous work, copyright CC Open Access Attribution 4.0 International, Small, Wiley).^[^ ^1^ ^]^

It is noteworthy to mention that even though most of the amino acids are relatively water soluble and possess bioactive properties, there have been reported that amongst these possibilities, some of them are more biocompatible, bioactive, stable, and with higher levels of water solubility. These specification superiorities can give a direction for choosing the ideal candidates for inducing chirality in specific MXenes/MBenes. In addition to the promising performance of cysteine (C_3_H_7_NO_2_S)‐based amino acids, this list includes serine (C_3_H_7_NO_3_), threonine (C_4_H_9_NO_3_), aspartic acid (C_4_H_7_NO_4_), glutamic acid (C_5_H_9_NO_4_), histidine (C_6_H_9_N_3_O_2_), lysine (C_6_H_14_N_2_O_2_), asparagine (C_4_H_8_N_2_O_3_), glutamine (C_5_H_10_N_2_O_3_), arginine (C_6_H_14_N_4_O_2_), tyrosine (C_9_H_11_NO_3_), and proline (C_5_H_9_NO_2_) (see **Figure** **5**; Figure S5, Supporting Information). In particular, the reactivity, superiority, and limitations of using each amino acid vacancy are accordingly discussed, and the proposed chemical reactions, purification steps, and detailed structural predictions are provided, paving the way toward their prioritized construction for systematic properties measurements. A robust comparison must evaluate the superiority of each new MXene and MBene with its similar l‐/d‐handed chiral source.

### 3.2 Proposed Reactions of Converting 2D Ti_3_C_2_T_x_ Sheets to Mixed‐Dimensional Chiral MXenes

The initial proof‐of‐concept on the underlying chemical reactions and mechanisms associated with converting Ti_3_C_2_T_x_ MXene nanosheets to mixed‐low‐dimensional chiral heterostructures and the creation of highly stable 2D‐0D/1D MXene‐based biomaterials is elucidated in our recent work. We proposed stoichiometric chemical reactions based on three primary equations and subsequent density functional theory (DFT) calculations, describing the possible reactions between Ti_3_C_2_T_x_ MXene surface‐terminated nanosheets, water molecules, surface titanium oxides, EDC, NHS, and the cysteine ligands. In particular, the proposed first reaction represents the direct interaction of Ti_3_C_2_T_x_ nanosheets with the air‐available or dissolved oxygen molecules in aqueous media. This step includes the dispersion, gradual oxidation, and hydrolysis‐based reactions involving the spontaneous formation of stable titanium oxide‐based phases such as TiO_2_ anatase on the surface of MXene flakes. The reaction is described with reasonable stability of surface interactions in water rather than a permanent covalent‐based bond. The second step covered the activation and crosslinking reactions of EDC/NHS solutions with carboxyl‐terminated MXene surfaces. In particular, the intensity of functional hydroxyl/carboxyl groups of these MXene nanosheets increases and is activated through surface‐modification and post‐functionalization of the EDC/NHS cross‐linker and stabilizer agent by forming NHS ester‐based bonds, contributing to avoiding any further oxidation or hydrolysis reactions of the intermediates. The third and last step includes the chiral functionalization with left‐ or right‐handed cysteine aqueous solutions. The NHS‐based ester and other available functional groups of as‐treated MXene surfaces react with the amine group of these cysteine enantiomers, contributing to form amide‐based bonds, and the NHS groups are subsequently displaced/released as water‐soluble by‐products. As a result, quality chiral MXene products with high material stability, biocompatibility, and bioactivity can be constructed through stable covalent bonds. The by‐product in the solutions washes away eventually from these colloidal flakes, providing pure chiral MXenes with a pH similar to or close to the MilliQ water. Accordingly, we balanced the reaction with new amino acids and proposed novel stoichiometric reactions for inducing the typical amino‐acid‐based cysteine chirality in other chemical compositions of MXenes in a group‐wise periodic fashion, which has not yet been synthesized or ever reported.

#### 3.2.1 Proposed Reactions for Creating Chiral Ti_3_C_2_T_x_ MXene and Ti‐MBene Nanosheets Using Serine

By substituting cysteine with serine in the original reactions, the first and second steps remain unchanged; rather than replacing C_3_H_7_NO_2_S with C_3_H_7_NO_3_ is applicable. Where instead of the thiol group (−SH) of cysteine that will be part of the bond formation, the available hydroxyl group in the structure of serine's subside chains will be involved in the reaction. The MXene/MBene products can still contain titanium‐oxygen‐carbon‐based bonds (Ti−O−C) and covalent carbon‐amine‐based bonds (C−N). However, the serine ligands, rather than cysteine, are anticipated to stably bind with the material surfaces (representative Ti_3_C_2_T_x_ as a common MXene). The serine functionalization reaction of Ti_3_C_2_T_x_ MXene would, therefore, be as shown accordingly. In particular, the first reaction represents a simplified “oxidation/hydrolysis” step, elaborating a partial oxidation or surface hydrolysis of MXene/MBene in aqueous media. In reality, byproducts in this reaction might be more complex than just H_2_. This step is followed by the second reaction, which includes EDC/NHS activation of surface carboxylic acid. Byproducts from EDC (urea derivatives) are omitted for simplicity. The result is an NHS ester anchored on the MXenes/MBene surface. The third reaction is proposed for the coupling of an amino acid (or other amine‐bearing ligands) with the surface NHS ester. For the other introduced amino acids, a similar trend is anticipated for Reactions 1 and 2 in theory. However, the third reaction is balanced based on the specific chemical formula of that particular amino acid with stoichiometric adjustments. Therefore, for the rest of the list, only Reaction 3 is presented for each individual amino acid. The proposed reactions for serine‐functionalized Ti_3_C_2_T_x_ MXene and Ti‐MBenes are as below. A similar trend is anticipated for effective chiral induction in MBenes with serine and other amino acids by EDC/NHS crosslinking.

(1) $$\left(\mathrm{Reaction}\;1\right)\;Ti_{3}C_{2}T_{x}+H_{2}O+O_{2}\to Ti_{3}C_{2}T_{x}/\mathrm{Ti}O_{2}+H_{2}$$

(2) $$\begin{array}{ccc} & & \left(\mathrm{Reaction}\;2\right)\;Ti_{3}C_{2}T_{x}/\mathrm{Ti}O_{2}-\mathrm{COOH}+\mathrm{EDC}+\mathrm{NHS}\\ & & \;\to Ti_{3}C_{2}T_{x}/\mathrm{Ti}O_{2}-C\left(=O\right)-O-C_{4}H_{4}NO_{3} \end{array}$$

(3) $$\begin{array}{ccc} & & \left(\mathrm{Reaction}\;3\right)\;Ti_{3}C_{2}T_{x}/\mathrm{Ti}O_{2}-C\left(=O\right)-O-C_{4}H_{4}NO_{3}\\ & & \;+C_{3}H_{7}NO_{3}\to Ti_{3}C_{2}T_{x}/\mathrm{Ti}O_{2}-C\left(=O\right)\\ & & \;-\mathrm{NH}-C_{3}H_{7}NO_{3}+C_{4}H_{5}NO_{3} \end{array}$$

(4) $$\begin{array}{ccc} & & \left(*R.1\;\mathrm{for}\;\mathrm{MBene}\right)\;\mathrm{TiB}T_{x}+4H_{2}O+1/2O_{2}\\ & & \;\to \mathrm{Ti}O_{2}+H_{3}BO_{3}+2H_{2} \end{array}$$

(5) $$\left(**R.1\right)\;\mathrm{Ti}B_{2}T_{x}+6H_{2}O+1/2O_{2}\to \mathrm{Ti}O_{2}+2H_{3}BO_{3}+3H_{2}$$

(6) $$\left(***R.1\right)\;Ti_{2}B_{2}T_{x}+8H_{2}O+O_{2}\to \mathrm{Ti}O_{2}+2H_{3}BO_{3}+4H_{2}$$

(7) $$\begin{array}{ccc} & & \left(*R.2\;\mathrm{for}\;\mathrm{MBene}\right)\;\mathrm{TiB}T_{x}/\mathrm{Ti}B_{2}T_{x}/Ti_{2}B_{2}T_{x}-\mathrm{Ti}O_{2}\\ & & \;-\mathrm{COOH}+\mathrm{EDC}+\mathrm{NHS}\to \mathrm{TiB}T_{x}/\mathrm{Ti}B_{2}T_{x}/Ti_{2}B_{2}T_{x}-\mathrm{Ti}O_{2}\\ & & \;-C\left(=O\right)-O-C_{4}H_{4}NO_{3} \end{array}$$

(8) $$\begin{array}{ccc} & & \left(*R.3\;\mathrm{for}\;\mathrm{MBene}\right)\;\mathrm{TiB}T_{x}/\mathrm{Ti}B_{2}T_{x}/Ti_{2}B_{2}T_{x}-\mathrm{Ti}O_{2}-C\left(=O\right)\\ & & \;-O-C_{4}H_{4}NO_{3}+C_{3}H_{7}NO_{3}\to \mathrm{TiB}T_{x}/\mathrm{Ti}B_{2}T_{x}/Ti_{2}B_{2}T_{x}\\ & & \;-\mathrm{Ti}O_{2}-C\left(=O\right)-\mathrm{NH}-C_{3}H_{7}NO_{3}+C_{4}H_{5}NO_{3} \end{array}$$

#### 3.2.2 Proposed Reactions for Creating Chiral Ti_3_C_2_T_x_ MXene Nanosheets Using Threonine

The stoichiometry in the threonine functionalization of MXene remains mainly unchanged compared to that proposed for cysteine due to the presence of amine groups in the composition of threonine to react subsequently with the NHS ester. Indeed, unlike cysteine, threonine is enriched with hydroxyl‐based groups in its sub‐side chains. However, these hydroxyl‐based groups are not anticipated theoretically to react and participate in the proposed coupling reactions. The hydroxyl terminals likely interact with the MXene surfaces through non‐covalent hydrogen bonding or other weakened interactions. Furthermore, due to the absence of thiol groups in the chemical structure of threonine, the reaction and binding to the MXene surface are expected to be performed through the amide‐based bonds. Indeed, using threonine instead of cysteine is also chemically feasible in theory. This slight difference might not cause significant differences or interfere with the covalent coupling reactions in the propped reaction relative to cysteine. The proposed threonine functionalization reaction of Ti_3_C_2_T_x_ MXene has been shown accordingly.

(9) $$\begin{array}{ccc} & & \left(\mathrm{Reaction}\;3\right)Ti_{3}C_{2}T_{x}/\mathrm{Ti}O_{2}-C\left(=O\right)-O-C_{4}H_{4}NO_{3}\\ & & \;+C_{4}H_{9}NO_{3}\to Ti_{3}C_{2}T_{x}/\mathrm{Ti}O_{2}-C\left(=O\right)\\ & & \;-\mathrm{NH}-C_{4}H_{9}NO_{3}+C_{4}H_{5}NO_{3} \end{array}$$

#### 3.2.3 Proposed Reactions for Creating Chiral Ti_3_C_2_T_x_ MXene Nanosheets Using Aspartic Acid

The proposed stoichiometry in the functionalization of MXene using aspartic acid is likely feasible from a chemistry point of view. In particular, aspartic acid has amine groups in its backbone structure and additional carboxyl‐based groups in its sub‐side chains (mainly in the β‐position). The unique presence of two carboxyl‐based groups in the chemical structure of aspartic acid is of important note. Indeed, the sidecarboxyl group in its chain is not theoretically feasible to directly participate and react with the NHS ester. These side‐chain groups remain free without any covalent bindings, which may indirectly contribute to interacting with its active surface sub‐sites in non‐covalent interactions without interfering with forming amide bonds in the solution systems. Hence, it might be feasible to react and bind with the MXene surfaces due to the imposed additional negative charges, likely due to the deprotonation of carboxyl‐based compounds in water or through the electrostatic interactions. Both might contribute to enhancing the colloidal dispersibility and/or stability of the functionalized MXene in aqueous media. Subsequently, the primary stable reactions likely occur through indirect coupling of the available amine group in aspartic acid with the NHS ester‐based groups that apparently formed on the surface of Ti_3_C_2_T_x_ and related metal surface oxide particles. As mentioned, aspartic acid has two carboxyl groups, but only its primary amine participates in EDC/NHS coupling. The reaction has shown accordingly.

(10) $$\begin{array}{ccc} & & \left(\mathrm{Reaction}\;3\right)\;Ti_{3}C_{2}T_{x}/\mathrm{Ti}O_{2}-C\left(=O\right)-O-C_{4}H_{4}NO_{3}\\ & & \;+C_{4}H_{7}NO_{4}\to Ti_{3}C_{2}T_{x}/\mathrm{Ti}O_{2}-C\left(=O\right)\\ & & \;-\mathrm{NH}-C_{4}H_{7}NO_{4}+C_{4}H_{5}NO_{3} \end{array}$$

#### 3.2.4 Proposed Reactions for Creating Chiral Ti_3_C_2_T_x_ MXene Nanosheets Using Glutamic Acid

The glutamic acid functionalization of Ti_3_C_2_T_x_ MXene would be described accordingly. Glutamic acid has two carboxyl‐based groups in its chemical structure, one in its backbone, which would partly contribute to forming amide‐based bonds. The second group may be attributed to its side chains. The structure of this amino acid possesses amine groups in its backbone, which can readily react with the activated carboxyl‐based sites available on the structure or formed on the surface of MXenes. These side‐chain groups theoretically may not directly participate in the reaction with the NHS‐ester groups. However, they may positively influence/modify the surface properties of colloidal systems by impacting additional surface negative charges. The primary carboxyl‐based groups of glutamic acid might be activated by EDC/NHS reagents through the reaction with its amine groups, forming the stable amide bond. Additionally, its side‐chain carboxyl‐based groups might indirectly be involved in the crosslinking process (apart from and not expected from EDC reaction activation). The side‐chain groups may also contribute to altering the surface charge of the colloids to involve in possible electrostatic interactions in the pH of water, enhancing the stability and dispersibility of final products.

(11) $$\begin{array}{ccc} & & \left(\mathrm{Reaction}\;3\right)\;Ti_{3}C_{2}T_{x}/\mathrm{Ti}O_{2}-C\left(=O\right)-O-C_{4}H_{4}NO_{3}\\ & & \;+C_{5}H_{9}NO_{4}\to Ti_{3}C_{2}T_{x}/\mathrm{Ti}O_{2}-C\left(=O\right)\\ & & \;-\mathrm{NH}-C_{5}H_{9}NO_{4}+C_{4}H_{5}NO_{3} \end{array}$$

#### 3.2.5 Proposed Reactions for Creating Chiral Ti_3_C_2_T_x_ MXene Nanosheets Using Histidine

The chiral functionalization of Ti_3_C_2_T_x_ MXene with histidine amino acid ligands has been described accordingly. First, the amine group available in the structure of histidine will likely react with the NHS‐ester groups, which have been formed on the MXene surfaces through amide‐based bonds. Notably, the imidazole rings in the structure of histidine‐based amino acids are not able to theoretically participate in direct coupling reactions in aqueous colloidal systems. However, its specifications enable surface properties modification due to its ability to interact with metal ions. Histidine has an imidazole ring that normally does not undergo direct EDC/NHS coupling. Thus, a feasible trend for chiral functionalization of the MXene with histidine is anticipated as below:

(12) $$\begin{array}{ccc} & & \left(\mathrm{Reaction}\;3\right)\;Ti_{3}C_{2}T_{x}/\mathrm{Ti}O_{2}-C\left(=O\right)-O-C_{4}H_{4}NO_{3}\\ & & \;+C_{6}H_{9}N_{3}O_{2}\to Ti_{3}C_{2}T_{x}/\mathrm{Ti}O_{2}-C\left(=O\right)\\ & & \;-\mathrm{NH}-C_{6}H_{9}N_{3}O_{2}+C_{4}H_{5}NO_{3} \end{array}$$

#### 3.2.6 Proposed Reactions for Creating Chiral Ti_3_C_2_T_x_ MXene Nanosheets Using Lysine

Chiral functionalization with lysine ligands would be challenging. Likewise, other amino acids, lysine‐based amino acids, may be involved in the final binding reaction through their active amine groups in the side chain. These bonds may react with the NHS‐ester‐based groups formed on the surface of Ti_3_C_2_T_x_ MXene and corresponding metal oxides such as TiO_2_ through stable amide bonds. This might be the most stable and primary reaction in this aqueous system. Notably, due to the available long aliphatic groups in the side chain structure of lysine, inducing a higher level of hydrophobic properties of the final MXene products is anticipated, which may not be ideal for the MXene hydrophilic character. However, it might enable higher capability for drug delivery applications, where a combination of hydrophilic and hydrophobic surface properties is beneficial. The structural groups of lysine (aliphatic side chains and amine groups) may also impose a positive charge to the surface of typical MXenes of the solution, but can provide more balanced properties. This might affect the MXene's interactions with other charged molecules/materials/compounds. The lysine functionalization reaction of Ti_3_C_2_T_x_ MXene has been shown accordingly.

(13) $$\begin{array}{ccc} & & \left(\mathrm{Reaction}\;3\right)\;Ti_{3}C_{2}T_{x}/\mathrm{Ti}O_{2}-C\left(=O\right)-O-C_{4}H_{4}NO_{3}\\ & & \;+C_{6}H_{14}N_{2}O_{2}\to Ti_{3}C_{2}T_{x}/\mathrm{Ti}O_{2}-C\left(=O\right)\\ & & \;-\mathrm{NH}-C_{6}H_{14}N_{2}O_{2}+C_{4}H_{5}NO_{3} \end{array}$$

#### 3.2.7 Proposed Reactions for Creating Chiral Ti_3_C_2_T_x_ MXene Nanosheets Using Asparagine

The asparagine functionalization of Ti_3_C_2_T_x_ MXene has been described accordingly. In the final step, the amine groups available in the backbone of asparagine‐based amino acids will likely react with the NHS‐ester groups formed on the MXene surfaces, forming stable amide bonds. Notably, the amide side chain of asparagine may not participate in the direct crosslinking reactions with other components in this system, but it might remain stable and interact with the side chains of the functionalized molecule available in aqueous MXene‐EDC/NHS solutions. It is important to note that the polar functional groups in asparagine can potentially contribute to further enhancing the hydrophilicity, dispersibility, and biocompatibility of the functionalized chiral MXenes, where high levels of hydrophilicity are key for specific applications.

(14) $$\begin{array}{ccc} & & \left(\mathrm{Reaction}\;3\right)Ti_{3}C_{2}T_{x}/\mathrm{Ti}O_{2}-C\left(=O\right)-O-C_{4}H_{4}NO_{3}\\ & & \;+C_{4}H_{8}N_{2}O_{3}\to Ti_{3}C_{2}T_{x}/\mathrm{Ti}O_{2}-C\left(=O\right)\\ & & \;-\mathrm{NH}-C_{4}H_{8}N_{2}O_{3}+C_{4}H_{5}NO_{3} \end{array}$$

#### 3.2.8 Proposed Reactions for Creating Chiral Ti_3_C_2_T_x_ MXene Nanosheets Using Glutamine

The glutamine functionalization of Ti_3_C_2_T_x_ MXene would be mainly similar to asparagine. In particular, the amine group available in the backbone of glutamine‐based amino acids can react with the NHS‐ester groups formed on the surface of MXene carbides/oxides by forming stable amide bonds. Besides, the amide‐based groups (─CONH_2_) available in the side chain of glutamine likely remain in these aqueous media for further surface properties modifications. The glutamine functionalization reaction of Ti_3_C_2_T_x_ MXene has been shown accordingly.

(15) $$\begin{array}{ccc} & & \left(\mathrm{Reaction}\;3\right)\;Ti_{3}C_{2}T_{x}/\mathrm{Ti}O_{2}-C\left(=O\right)-O-C_{4}H_{4}NO_{3}\\ & & \;+C_{5}H_{10}N_{2}O_{3}\to Ti_{3}C_{2}T_{x}/\mathrm{Ti}O_{2}-C\left(=O\right)\\ & & \;-\mathrm{NH}-C_{5}H_{10}N_{2}O_{3}+C_{4}H_{5}NO_{3} \end{array}$$

#### 3.2.9 Proposed Reactions for Creating Chiral Ti_3_C_2_T_x_ MXene Nanosheets Using Arginine

The chiral functionalization of Ti_3_C_2_T_x_ MXene with arginine would be somewhat similar to glutamine and asparagine. In a similar process, the amine groups available in the backbone of arginine likely react with NHS‐ester groups to amide bonds on the MXene surfaces. Notably, the guanidinium‐based group in the side chain of arginine (─C(NH_2_)_2_), even though not directly participate in the bonding/crosslinking reactions, can remain active to contribute to the compositional structures of final functionalized chiral MXene products. As of particular note, the −C(NH_2_)_2_ groups are highly basic, and their presence in the colloidal systems may affect increased surface positive charges at pHs slightly higher than pure water. In case of interaction with organic molecules or salt‐based culture media, which have a higher pHs than MilliQ water, this alternation may contribute to positively influence the direct interaction of MXenes with bio‐systems and related macromolecules, such as DNA, RNA, proteins, and metabolites, which are often negatively charged. This interaction may further improve the capability of MXene materials to be selectively taken up by specific cells and tissues, improving their long‐term biocompatibility properties. This aspect may also be suitable for applications where electrostatic interactions are beneficial, together with promising biocompatibility and bioactivity of arginine‐modified MXenes for specific bio‐applications such as drug delivery, biological sensing, immune modulation, and tissue engineering. The arginine functionalization reaction of Ti_3_C_2_T_x_ has been shown accordingly.

(16) $$\begin{array}{ccc} & & \left(\mathrm{Reaction}\;3\right)\;Ti_{3}C_{2}T_{x}/\mathrm{Ti}O_{2}-C\left(=O\right)-O-C_{4}H_{4}NO_{3}\\ & & \;+C_{6}H_{14}N_{4}O_{2}\to Ti_{3}C_{2}T_{x}/\mathrm{Ti}O_{2}-C\left(=O\right)\\ & & \;-\mathrm{NH}-C_{6}H_{14}N_{4}O_{2}+C_{4}H_{5}NO_{3} \end{array}$$

#### 3.2.10 Proposed Reactions for Creating Chiral Ti_3_C_2_T_x_ MXene Nanosheets Using Tyrosine

Chiral functionalization with tyrosine ligands would be partially challenging. In particular, tyrosine naturally has hydroxyl groups on its aromatic ring and has been reported to possess moderate water‐solubility compared to the aforementioned amino acid ligands. In a relatively similar reaction, the amine group available in the backbone of tyrosine amino acid can react with the NHS‐ester on the surface of MXenes, forming stable amide bonds. The phenolic hydroxyl group existing in the aromatic ring of this amino acid may not allow it to be incorporated directly into the EDC/NHS crosslinking reaction. However, due to its bioactive properties, it may play a role in subsequent modification and positively influence the electrostatic properties and bio‐related interactions of the functionalized chiral MXenes with tyrosine. Notably, due to the presence of phenolic groups in its chemical structure, it can readily interact with other molecules and form hydrogen bonds, making the containing material more interactive with biological systems, where hydrophobicity is important, such as direct interactions with enzymes, receptors, or other related bio‐macromolecules. In addition, its aromatic ring may contribute to creating π–π interactions with other aromatic structures/compounds, providing exciting avenues of studies for evaluating the efficacy of these chiral MXenes targeted drug delivery, biological tracking, and/or immune modulation. Indeed, the enrichment of tyrosine with phenolic hydroxyl and amine groups can potentially make it relatively more hydrophilic, where the hydrophilicity of MXenes is an assessment for bio‐applications. Besides, the hydrogen bonding and electrostatic interaction properties of the phenolic group of this amino acid could influence the material stability of MXene end‐products. The tyrosine functionalization of Ti_3_C_2_T_x_ MXene has been shown accordingly.

(17) $$\begin{array}{ccc} & & \left(\mathrm{Reaction}\;3\right)\;Ti_{3}C_{2}T_{x}/\mathrm{Ti}O_{2}-C\left(=O\right)-O-C_{4}H_{4}NO_{3}\\ & & \;+C_{9}H_{11}NO_{3}\to Ti_{3}C_{2}T_{x}/\mathrm{Ti}O_{2}-C\left(=O\right)\\ & & \;-\mathrm{NH}-C_{9}H_{11}NO_{3}+C_{4}H_{5}NO_{3} \end{array}$$

#### 3.2.11 Proposed Reactions for Creating Chiral Ti_3_C_2_T_x_ MXene Nanosheets Using Proline

The structure and behavior of proline in the chiral functionalization of MXenes are anticipated to be, to some extent, similar to tyrosine, except that proline has pyrrolidine rings in its chemical structure (C_5_H_9_NO_2_). l‐ and d‐handed enantiomers are technically and commercially available for all these amino acids, supporting their potential for constructing chiral‐engineered MXenes.

### 3.3 Proposed Reactions for Other Chemical Compositions of MXenes

- Zirconium‐carbide‐based chiral MXenes:

Next, we proposed the feasibility of our model to be applied and adapted to each amino acid for other chemical compositions of MXenes. In particular, it is anticipated that the proposed reaction for chiral engineering of Ti_3_C_2_T_x_ MXene can be adapted for zirconium carbide‐based MXenes (e.g., Zr_3_C_2_T_x_) and other available MXene compositions by simply replacing the specific amino acid in their reaction procedure and adjusting/balancing to that specific MXene's chemistry. For instance, in the case of zirconium and cysteine amino acid ligands, the reactions have been proposed accordingly based on their location in the periodic table of MXenes.

The fabrication of zirconium and hafnium carbide/nitride‐based MXenes is expected to be similar to other MXene compositions, such as Ti_3_C_2_T_x_. However, due to their chemical differences from titanium or other common transition metals, specific considerations may be needed for both synthesis and surface modifications. In particular, their MAX‐phases have relatively higher lattice energies and stronger M–A bonds compared to their titanium counterpart, making them more tolerant against etching and exfoliation.^[^ ^38^, ^39^ ^]^ Therefore, to effectively remove the “A” layers from their MAX phases, a higher concentration of etchant or longer treatment duration is often required. For surface modification and particularly chirality induction, these considerations may need to be applied with higher chiral concentrations, or a more elongated chirality induction time may be required. Moreover, the higher atomic size and electronegativity of zirconium and hafnium may influence the interaction or coordinative interactions with amino acid ligands (side chains), resulting in different binding energies or surface reactivity. On the other hand, due to their high mechanical, electrophysical, and optical properties, they might be further beneficial for specific bio‐applications that require more resistant and electroactive materials.

(18) $$\left(\mathrm{Reaction}\;1\right)Zr_{3}C_{2}T_{x}+H_{2}O+O_{2}\to Zr_{3}C_{2}T_{x}/\mathrm{Zr}O_{2}+H_{2.}$$

(19) $$\begin{array}{ccc} & & \left(\mathrm{Reaction}\;2\right)\;Zr_{3}C_{2}T_{x}/\mathrm{Zr}O_{2}-\mathrm{COOH}+\mathrm{EDC}+\mathrm{NHS}\\ & & \;\to Zr_{3}C_{2}T_{x}/\mathrm{Zr}O_{2}-C\left(=O\right)-O-C_{4}H_{4}NO_{3}. \end{array}$$

(20) $$\begin{array}{ccc} & & \left(\mathrm{Reaction}\;3\right)Zr_{3}C_{2}T_{x}/\mathrm{Zr}O_{2}-C\left(=O\right)-O-C_{4}H_{4}NO_{3}\\ & & \;+C_{4}H_{5}NO_{3}\to Zr_{3}C_{2}T_{x}/\mathrm{Zr}O_{2}-C\left(=O\right)\\ & & \;-\mathrm{NH}-C_{4}H_{4}NO_{3}+C_{3}H_{7}NO_{3}. \end{array}$$

- Hafnium‐carbide‐based chiral MXenes:
(21) $$\begin{array}{ccc} & & \left(\mathrm{Reaction}\;1\right)\;Hf_{3}C_{2}T_{x}/Hf_{4}C_{3}T_{x}+H_{2}O+O_{2}\\ & & \;\to Hf_{3}C_{2}T_{x}/Hf_{4}C_{3}T_{x}-\mathrm{Hf}O_{2}+H_{2.} \end{array}$$
(22) $$\begin{array}{ccc} & & \left(\mathrm{Reaction}\;2\right)\;Hf_{3}C_{2}T_{x}/Hf_{4}C_{3}T_{x}-\mathrm{Hf}O_{2}\\ & & \;-\mathrm{COOH}+\mathrm{EDC}+\mathrm{NHS}\to Hf_{3}C_{2}T_{x}/Hf_{4}C_{3}T_{x}\\ & & \;-\mathrm{Hf}O_{2}-C\left(=O\right)-O-C_{4}H_{4}NO_{3.} \end{array}$$
(23) $$\begin{array}{ccc} & & \left(\mathrm{Reaction}\;3\right)\;Hf_{3}C_{2}T_{x}/Hf_{4}C_{3}T_{x}-\mathrm{Hf}O_{2}-C\left(=O\right)\\ & & \;-O-C_{4}H_{4}NO_{3}+C_{4}H_{5}NO_{3}\to Hf_{3}C_{2}T_{x}/Hf_{4}C_{3}T_{x}\\ & & \;-\mathrm{Hf}O_{2}-C\left(=O\right)-\mathrm{NH}-C_{4}H_{4}NO_{3}+C_{3}H_{7}NO_{3}. \end{array}$$
- Vanadium‐carbide‐based chiral MXenes:
(24) $$\begin{array}{ccc} & & \left(\mathrm{Reaction}\;1\right)V_{3}C_{2}T_{x}/V_{4}C_{3}T_{x}+H_{2}O+O_{2}\\ & & \;\to V_{3}C_{2}T_{x}/V_{4}C_{3}T_{x}-VO_{2}/V_{2}O_{5}+H_{2}. \end{array}$$
(25) $$\begin{array}{ccc} & & \left(\mathrm{Reaction}\;2\right)V_{3}C_{2}T_{x}/V_{4}C_{3}T_{x}-VO_{2}/V_{2}O_{5}\\ & & \;-\mathrm{COOH}+\mathrm{EDC}+\mathrm{NHS}\to V_{3}C_{2}T_{x}/V_{4}C_{3}T_{x}\\ & & \;-VO_{2}/V_{2}O_{5}-C\left(=O\right)-O-C_{4}H_{4}NO_{3}. \end{array}$$
(26) $$\begin{array}{ccc} & & \left(\mathrm{Reaction}\;3\right)V_{3}C_{2}T_{x}/V_{4}C_{3}T_{x}-VO_{2}/V_{2}O_{5}-C\left(=O\right)\\ & & \;-O-C_{4}H_{4}NO_{3}+C_{4}H_{5}NO_{3}\to V_{3}C_{2}T_{x}/V_{4}C_{3}T_{x}\\ & & \;-VO_{2}/V_{2}O_{5}-C\left(=O\right)-\mathrm{NH}-C_{4}H_{4}NO_{3}+C_{3}H_{7}NO_{3} \end{array}$$
- Niobium‐carbide‐based chiral MXenes:
(27) $$\begin{array}{ccc} & & \left(\mathrm{Reaction}\;1\right)\;Nb_{2}CT_{x}/Nb_{3}C_{2}T_{x}/Nb_{4}C_{3}T_{x}+H_{2}O+O_{2}\\ & & \;\to Nb_{2}CT_{x}/Nb_{3}C_{2}T_{x}/Nb_{4}C_{3}T_{x}-\mathrm{NbO}/\mathrm{Nb}O_{2}/Nb_{2}O_{5}+H_{2}.\;\;\; \end{array}$$
(28) $$\begin{array}{ccc} & & \left(\mathrm{Reaction}\;2\right)Nb_{2}CT_{x}/Nb_{3}C_{2}T_{x}/Nb_{4}C_{3}T_{x}-\mathrm{NbO}/\mathrm{Nb}O_{2}/Nb_{2}O_{5}\\ & & \;-\mathrm{COOH}+\mathrm{EDC}+\mathrm{NHS}\to Nb_{2}CT_{x}/Nb_{3}C_{2}T_{x}/Nb_{4}C_{3}T_{x}\\ & & \;-\mathrm{NbO}/\mathrm{Nb}O_{2}/Nb_{2}O_{5}-C\left(=O\right)-O-C_{4}H_{4}NO_{3.} \end{array}$$
(29) $$\begin{array}{ccc} & & \left(\mathrm{Reaction}\;3\right)Nb_{2}CT_{x}/Nb_{3}C_{2}T_{x}/Nb_{4}C_{3}T_{x}-\mathrm{NbO}/\mathrm{Nb}O_{2}/Nb_{2}O_{5}\\ & & \;-C\left(=O\right)-O-C_{4}H_{4}NO_{3}+C_{4}H_{5}NO_{3}\\ & & \;\to Nb_{2}CT_{x}/Nb_{3}C_{2}T_{x}/Nb_{4}C_{3}T_{x}-\mathrm{NbO}/\mathrm{Nb}O_{2}/Nb_{2}O_{5}\\ & & \;-C\left(=O\right)-\mathrm{NH}-C_{4}H_{4}NO_{3}+C_{3}H_{7}NO_{3}. \end{array}$$
- Tantalum‐carbide‐based chiral MXenes:
(30) $$\begin{array}{ccc} & & \left(\mathrm{Reaction}\;1\right)\;Ta_{4}C_{3}T_{x}/Ta_{2}CT_{x}+H_{2}O+O_{2}\\ & & \;\to Ta_{4}C_{3}T_{x}/Ta_{2}CT_{x}-\mathrm{TaO}/\mathrm{Ta}O_{2}/Ta_{2}O_{5}+H_{2}. \end{array}$$
(31) $$\begin{array}{ccc} & & \left(\mathrm{Reaction}\;2\right)\;Ta_{4}C_{3}T_{x}/Ta_{2}CT_{x}-\mathrm{TaO}/\mathrm{Ta}O_{2}/Ta_{2}O_{5}\\ & & \;-\mathrm{COOH}+\mathrm{EDC}+\mathrm{NHS}\to Ta_{4}C_{3}T_{x}/Ta_{2}CT_{x}\\ & & \;-\mathrm{TaO}/\mathrm{Ta}O_{2}/Ta_{2}O_{5}-C\left(=O\right)-O-C_{4}H_{4}NO_{3} \end{array}$$
(32) $$\begin{array}{ccc} & & \left(\mathrm{Reaction}\;3\right)\;Ta_{4}C_{3}T_{x}/Ta_{2}CT_{x}-\mathrm{TaO}/\mathrm{Ta}O_{2}/Ta_{2}O_{5}\\ & & \;-C\left(=O\right)-O-C_{4}H_{4}NO_{3}+C_{4}H_{5}NO_{3}\\ & & \;\to Ta_{4}C_{3}T_{x}/Ta_{2}CT_{x}-\mathrm{TaO}/\mathrm{Ta}O_{2}/Ta_{2}O_{5}-C\left(=O\right)\\ & & \;-\mathrm{NH}-C_{4}H_{4}NO_{3}+C_{3}H_{7}NO_{3.} \end{array}$$
- Chromium‐carbide‐based chiral MXenes:
(33) $$\begin{array}{ccc} & & \left(\mathrm{Reaction}\;1\right)\;\;Cr_{2}CT_{x}/Cr_{4}C_{3}T_{x}+H_{2}O+O_{2}\\ & & \;\to Cr_{2}CT_{x}/Cr_{4}C_{3}T_{x}-\mathrm{Cr}O_{2}/Cr_{2}O_{3}+H_{2}. \end{array}$$
(34) $$\begin{array}{ccc} & & \left(\mathrm{Reaction}\;2\right)Cr_{2}CT_{x}/Cr_{4}C_{3}T_{x}-\mathrm{Cr}O_{2}/Cr_{2}O_{3}\\ & & \;-\mathrm{COOH}+\mathrm{EDC}+\mathrm{NHS}\to Cr_{2}CT_{x}/Cr_{4}C_{3}T_{x}\\ & & \;-\mathrm{Cr}O_{2}/Cr_{2}O_{3}-C\left(=O\right)-O-C_{4}H_{4}NO_{3}. \end{array}$$
(35) $$\begin{array}{ccc} & & \left(\mathrm{Reaction}\;3\right)Cr_{2}CT_{x}/Cr_{4}C_{3}T_{x}-\mathrm{Cr}O_{2}/Cr_{2}O_{3}-C\left(=O\right)\\ & & \;-O-C_{4}H_{4}NO_{3}+C_{4}H_{5}NO_{3}\to Cr_{2}CT_{x}/Cr_{4}C_{3}T_{x}\\ & & \;-\mathrm{Cr}O_{2}/Cr_{2}O_{3}-C\left(=O\right)-\mathrm{NH}-C_{4}H_{4}NO_{3}+C_{3}H_{7}NO_{3.} \end{array}$$
- Molybdenum‐carbide‐based chiral MXenes:
(36) $$\begin{array}{ccc} & & \left(\mathrm{Reaction}\;1\right)\;Mo_{2}CT_{x}/Mo_{3}C_{2}T_{x}/Mo_{4}C_{3}T_{x}+H_{2}O+O_{2}\\ & & \;\to Mo_{2}CT_{x}/Mo_{3}C_{2}T_{x}/Mo_{4}C_{3}T_{x}-\mathrm{MoO}/\mathrm{Mo}O_{3}+H_{2}. \end{array}$$
(37) $$\begin{array}{ccc} & & \mathrm{Reaction}\;2)\;\;Mo_{2}CT_{x}/Mo_{3}C_{2}T_{x}/Mo_{4}C_{3}T_{x}-\mathrm{MoO}/\mathrm{Mo}O_{3}\\ & & \;-\mathrm{COOH}+\mathrm{EDC}+\mathrm{NHS}\to Mo_{2}CT_{x}/Mo_{3}C_{2}T_{x}/Mo_{4}C_{3}T_{x}\\ & & \;-\mathrm{MoO}/\mathrm{Mo}O_{3}-C\left(=O\right)-O-C_{4}H_{4}NO_{3.} \end{array}$$
(38) $$\begin{array}{ccc} & & \left(\mathrm{Reaction}\;3\right)\;Mo_{2}CT_{x}/Mo_{3}C_{2}T_{x}/Mo_{4}C_{3}T_{x}-\mathrm{MoO}/\mathrm{Mo}O_{3}\\ & & \;-C\left(=O\right)-O-C_{4}H_{4}NO_{3}+C_{4}H_{5}NO_{3}\\ & & \;\to Mo_{2}CT_{x}/Mo_{3}C_{2}T_{x}/Mo_{4}C_{3}T_{x}-\mathrm{MoO}/\mathrm{Mo}O_{3}\\ & & \;-C\left(=O\right)-\mathrm{NH}-C_{4}H_{4}NO_{3}+C_{3}H_{7}NO_{3}. \end{array}$$
- Tungsten/manganese/cerium‐carbide‐based chiral MXenes:

Even though there have been few reports on the synthesis of these MXenes with a limited frequency, a similar trend is anticipated for the chiral functionalization of MXenes by amino acids.

### 3.4 Proposed Reactions/Possibilities for Functionalizing MXene with Other Chiral Sources

#### 3.4.1 Chiral Monosaccharides Sugars (e.g., d‐Glucose, d‐Galactose. l‐Arabinose, l‐Rhamnose)

These sugars are highly active chiral due to the natural presence of asymmetric carbon atoms in their sugar rings. They have proven the high levels of biocompatibility, as they can naturally occur in various biological systems. Additionally, they are highly water soluble due to the enrichment with the hydroxyl‐based group, making them ideal candidates for chiral functionalization of MXenes. A similar trend of chiral functionalization with cysteine‐based amino acids is anticipated with these sugar‐based molecules using the EDC/NHS method. The chiral polysaccharides such as chiral‐active chitosan derivatives and dextran, and specific nano‐carbon materials like chiral fullerenes (C60) or other chiral‐active related structures might also consider for study for chiral functionalization of MXenes. The proposed reactions have been accordingly shown for individual monosaccharides, including d‐glucose/galactose and l‐arabinose/rhamnose for the representative MXene prototypes (Ti_3_C_2_T_x_) with potential expansion for MBenes as well.

- d‐glucose/galactose (C_6_H_12_O_6_) functionalization:
(39) $$\left(\mathrm{Reaction}\;1\right)\;\;Ti_{3}C_{2}T_{x}+H_{2}O+O_{2}\to Ti_{3}C_{2}T_{x}/\mathrm{Ti}O_{2}+H_{2}.$$
(40) $$\begin{array}{ccc} & & \left(\mathrm{Reaction}\;2\right)Ti_{3}C_{2}T_{x}/\mathrm{Ti}O_{2}-\mathrm{COOH}+\mathrm{EDC}+\mathrm{NHS}\\ & & \;\to Ti_{3}C_{2}T_{x}/\mathrm{Ti}O_{2}-C\left(=O\right)-O-C_{4}H_{4}NO_{3.} \end{array}$$
(41) $$\begin{array}{ccc} & & \left(\mathrm{Reaction}\;3\right)\;Ti_{3}C_{2}T_{x}/\mathrm{Ti}O_{2}-C\left(=O\right)-O-C_{4}H_{4}NO_{3}\\ & & \;+C_{6}H_{12}O_{6}\to Ti_{3}C_{2}T_{x}/\mathrm{Ti}O_{2}-C\left(=O\right)\\ & & \;-\mathrm{NH}-C_{6}H_{12}O_{6}+C_{4}H_{5}NO_{3.} \end{array}$$
- l‐arabinose (C_5_H_10_O_5_) functionalization:
(42) $$\begin{array}{ccc} & & \left(\mathrm{Reaction}\;3\right)Ti_{3}C_{2}T_{x}/\mathrm{Ti}O_{2}-C\left(=O\right)-O-C_{4}H_{4}NO_{3}\\ & & \;+C_{5}H_{10}O_{5}\to Ti_{3}C_{2}T_{x}/\mathrm{Ti}O_{2}-C\left(=O\right)\\ & & \;-\mathrm{NH}-C_{5}H_{10}O_{5}+C_{4}H_{5}NO_{3} \end{array}$$
- l‐rhamnose (C_6_H_12_O_5_) functionalization:
(43) $$\begin{array}{ccc} & & \left(\mathrm{Reaction}\;3\right)\;Ti_{3}C_{2}T_{x}/\mathrm{Ti}O_{2}-C\left(=O\right)-O-C_{4}H_{4}NO_{3}\\ & & \;+C_{6}H_{12}O_{5}\to Ti_{3}C_{2}T_{x}/\mathrm{Ti}O_{2}-C\left(=O\right)\\ & & \;-\mathrm{NH}-C_{6}H_{12}O_{5}+C_{4}H_{5}NO_{3.} \end{array}$$

#### 3.4.2 Chiral Aromatic Compounds (e.g., Azobenzene (C_6_H_5_N_2_C_6_H_5_) or Thiol Derivatives)

- ***Azobenzene (C_6_H_5_N_2_C_6_H_5_) functionalization***:

Azobenzene derivatives are naturally chiral and exist in l‐ or d‐enantiomers. Likewise, the thiol groups of l‐/d‐handed cysteine amino acids, azobenzene‐based derivatives might also be capable of inducing/introducing chirality into the surface of MXenes nanomaterials. Azobenzene‐based derivatives have been reported for reasonable biocompatibility in controlled doses and certain contexts and are soluble in aqueous media; they are more likely applicable in optical and optoelectronic applications in biomedical fields. Functionalization with chiral‐active azobenzene‐based compounds can be performed by forming bonds with the NHS‐ester groups on the surface of MXene with stable binding with the amine group of chiral azobenzene. The functionalization of Ti_3_C_2_T_x_ MXene‐based dispersion with azobenzene has been proposed as below:

(44) $$\begin{array}{ccc} & & \left(\mathrm{Reaction}\;3\right)\;Ti_{3}C_{2}T_{x}/\mathrm{Ti}O_{2}-C\left(=O\right)-O-C_{4}H_{4}NO_{3}\\ & & \;+C_{12}H_{10}N_{2}/C_{12}H_{9}N_{3}\to Ti_{3}C_{2}T_{x}/\mathrm{Ti}O_{2}-C\left(=O\right)\\ & & \;-\mathrm{NH}-C_{12}H_{9}N_{3}+C_{4}H_{5}NO_{3}. \end{array}$$

#### 3.4.3 Chiral Active Transition Metals/Complexes

This vacancy covers the chiral functionalization of MXenes with complex systems such as cooper (Cu^2+^) or iron (Fe^3+^) complexes and in combination with amino acid ligands. Another chiral source in this category refers to active metal–organic frameworks (MOFs)‐based compounds. Despite their potential, these strategies may face technical challenges and limitations, including chemical complexity, cytotoxicity at higher doses, cost consideration, and the difficulties of controlling parameters for robust re‐productions. Case‐specific studies must be conducted to evaluate their capability for chiral engineering of MXene biomaterials.

#### 3.4.4 Chiral‐Active Natural/Edible Products

- ***Limonene (citrus terpene, C_10_H_16_) functionalization***:

The application of natural products is highlighted as beneficial from different aspects, including biocompatibility, production cost, and sustainability. Thus, confirming and validating their capability to functionalize MXene is of high research and development interest. Indeed, they are naturally occurring and biocompatible chiral compounds that have asymmetric carbon centers. Limonene and caffeine are typical representatives in this category, which are edible and commonly used in many biological industries and food products. The proposed reactions for functionalization of Ti_3_C_2_T_x_ MXene with these chiral caffeine ligands has been presented accordingly.

(45) $$\begin{array}{ccc} & & \left(\mathrm{Reaction}\;3\right)Ti_{3}C_{2}T_{x}/\mathrm{Ti}O_{2}-C\left(=O\right)-O-C_{4}H_{4}NO_{3}+C_{10}H_{16}\\ & & \;\to Ti_{3}C_{2}T_{x}/\mathrm{Ti}O_{2}-C\left(=O\right)-\mathrm{NH}-C_{10}H_{16}+C_{4}H_{5}NO_{3}. \end{array}$$

- ***Caffeine (alkaloid, C_8_H_10_N_4_O_2_) functionalization***:

The caffeine‐based chiral active compounds are water‐soluble, and this property of limonene can be improved through rational functionalization. Designing and developing chiral MXenes based on these compounds is promising for multiple targeted bio‐applications. Below are the proposed reactions for these chiral caffeine ligands on model Ti_3_C_2_T_x_ MXene.

(46) $$\begin{array}{ccc} & & \left(\mathrm{Reaction}\;3\right)\;Ti_{3}C_{2}T_{x}/\mathrm{Ti}O_{2}-C\left(=O\right)-O-C_{4}H_{4}NO_{3}\\ & & \;+C_{8}H_{10}N_{4}O_{2}\to Ti_{3}C_{2}T_{x}/\mathrm{Ti}O_{2}-C\left(=O\right)\\ & & \;-\mathrm{NH}-C_{8}H_{10}N_{4}O_{2}+C_{4}H_{5}NO_{3}. \end{array}$$

### 3.5 Natural/Synthetic Chiral DNA Systems for Functionalizing MXenes

Besides the mono/polysaccharides and protein structure, some other bio‐macromolecules also have the potential to be considered for developing chiral MXene biomaterials. For instance, DNA can be adopted as a chiral selector and immobilized in MXene‐based substrates for specific enantioselectively electrochemical recognition applications. Nonetheless, the studies using DNA are still in their infancy, with a limited number of publications thus far. Owing to their unique biochemical structures and properties, they may be considered the next‐generation distinctive functions when combined with bioactive nanomaterials like MXenes to induce multiple bioactivities in living systems. With DNA as representative biomolecules, the double helix compounds or DNA‐templated nano‐hybrid structures may allow DNA‐based chiral MXenes to further exhibit unique properties for biological, biomedical, and environmental applications. In addition, the DNA‐based bio‐constructs can make more contributions than just serving as chiral sources and have the capacity to be rationally conjugated with diverse MXene compositions to not only synthesize novel materials but also to address specific clinical, healthcare, and agricultural problems through merging MXene nanomaterials with biotechnology and medicine. To date, no particular study reported DNA‐mediated fabrication of MXene biomaterials. Therefore, designing new MXenes based on applying synthetic templates or natural biomolecules is highly beneficial in the bio‐related research field. For instance, chiral MXene‐supramolecular hydrogels/3D scaffolds may offer enhanced properties relative to pristine hydrogel for tissue regeneration, implant coating, stem cell therapies, drug delivery, bio‐stimulation, and immunoengineering. Further, these hybrid chiral supramolecular designs may provide effective methods for the absorption and precise release of anticancer or immunomodulatory chiral drugs, which have the potential for the combination of photothermal therapy and chemotherapy in advanced therapeutics. The area of disease diagnosis is another potential for chiral MXene biomaterials to perform as next‐generation MXene biosensors.

Chiral‐engineered MXenes, once specifically functionalized with amino acids, gain additional physicochemical attributes that can influence their interactions and bioactivity with DNA‐based structures. In particular, DNA interactions with MXenes can arise through electrostatic binding, intercalation, or cleavage‐like mechanisms through the latter, which typically require reactive sites. MXenes are rich in transition metals and partially oxidized surfaces, meaning they can sometimes chelate or bind to phosphate groups on the DNA backbone, altering the DNA conformation or hindering replication. When functionalized with amino acids or other chiral‐active compounds, these natural–hybrid or synthetic materials may carry additional cationic or zwitterionic domains (depending on the amino‐acid side chains and pH), potentially strengthening their association with negatively charged DNA. Further, under appropriate conditions (e.g., light activation or the presence of specific cofactors), the MXenes’ surface can catalyze oxidative damage to DNA bases. If cysteine or similarly reactive side chains are present on the MXene surface, they can modulate the local redox environment, possibly promoting or inhibiting DNA strand breaks depending on the reaction conditions. The chiral aspect of the functionalized MXene might preferentially bind to DNA grooves or superstructures in a stereospecific fashion, even though the extent of such stereoselective DNA binding is still largely a subject of ongoing research. In summary, DNA interactions occur through electrostatic attraction, metal‐assisted cleavage, or alterations of the local redox environment. By carefully selecting and attaching specific chiral‐active amino acid‐based ligands‐ particularly those with ionizable or redox‐active side chains‐ the surface properties of the base MXenes can be potentially tuned for enhanced bio‐functionalities as multifaceted platforms in bio‐related applications.

### 3.6 Key Considerations for Functionalizing Nitride‐Based MXenes with Chiral Sources

It is reasonable that the proposed strategies would be applicable to construct chiral active carbonatite or nitrite‐based MXenes. In particular, the surface oxidation and hydrolysis would form titanium nitride (e.g., Ti_4_N_3_T_x_) on the surface of these MXene nanosheets instead of typical TiO_2_, and the formation of Ti_3_N_2_/Ti_3_N_2_O_x_ is anticipated in the aqueous media as the result of hydrolysis and oxidation of the dispersed flakes. Surface functionalization by carboxyl and hydroxyl‐based groups is also anticipated to be reactive and could undergo activation with the EDC/NHS cross‐linker agent. Thus, the same reactions are anticipated except that in the functionalization process, for instance, Ti_4_N_3_T_x_ may readily form titanium nitride oxide Ti_4_N_3_O_x_ instead of typical TiO_2_. These changes are expected to not significantly affect or interfere with the core steps of chiral functionalization with amino acids or other chiral‐active sources. However, different phase, microstructural, and stability properties are anticipated in nitride‐based MXenes relative to typical carbide‐based compositions. Another potential possibility for chiral‐engineering of titanium‐based nitride MXene would Ti_2_NT_x_. The stability and functional properties of new chiral nitrite MXenes with these approaches need to be carefully evaluated for each possible composition and properly compared with other chiral MXenes (e.g., chiral Ti_3_C_2_T_x_). We have adapted the proposed chiral‐engineering reactions as an example for nitrate‐based MXenes accordingly.

(47) $$\begin{array}{c} \left(\mathrm{Reaction}\;1\right)\;\;Ti_{4}N_{3}T_{x}+H_{2}O+O_{2}\to Ti_{4}N_{3}O_{x}/\mathrm{Ti}O_{2}+H_{2}. \end{array}$$

(48) $$\begin{array}{ccc} & & \left(\mathrm{Reaction}\;2\right)\;Ti_{4}C_{3}T_{x}/Ti_{4}N_{3}O_{x}-\mathrm{COOH}+\mathrm{EDC}+\mathrm{NHS}\\ & & \;\to Ti_{4}N_{3}T_{x}/Ti_{4}N_{3}O_{x}-C\left(=O\right)-\mathrm{NH}-C_{3}H_{7}NO_{3}. \end{array}$$

(49) $$\begin{array}{ccc} & & \left(\mathrm{Reaction}\;3\right)\;Ti_{3}N_{2}T_{x}/\mathrm{Ti}4N_{3}O_{x}-\mathrm{COOH}+\mathrm{EDC}+\mathrm{NHS}\\ & & \;\to Ti_{4}N_{2}T_{x}/\mathrm{Ti}O_{2}-C\left(=O\right)-\mathrm{NH}-C_{4}H_{4}NO_{3}+C_{3}H_{7}NO_{3}.\; \end{array}$$

### 3.7 Prospective Vacancies for Chiral Engineering of Different Compositions of MBenes (Experimental Evidence or in Theory)

Recently, several theoretical or experimental syntheses and bio/applications of MBenes have been reported.^[^ ^31^, ^32^, ^33^, ^34^ ^]^ MBenes possess striking similarities in structure with MXenes. However, their unique features, such as higher mechanical and stability properties, enable their specific functions for different fields, including bio‐applications. To date, compared to MXenes, a lower frequency of MBenes has been reported, while growing fast in both synthesis and applications. From the chemistry perspective, possible MBenes formulations can be potently subjected to introduced chiral engineering strategies (see Figure 4; Figure S5, Supporting Information). Thus, synthesis and modification of MBenes, including MBs (e.g., ScB, TiB, VB, YB, ZrB, NbB, MnB, MoB, FeB, HfB, TaB, WB), MB_2_ (i.e., FeB_2_, RuB_2_, OsB_2_), M_2_B_2_ (i.e., V_2_B_2_, Fe_2_B_2_, Cr_2_B_2_, Mn_2_B_2_), and M_3_B_4_ (i.e., Nb_3_B_4_, Ta_3_B_4_, Cr_3_B_4_), and (M′_2/3_M″_1/3_)_2_B_2_ MBenes (Fe_2/3_Sc_1/3_)_2_B_2,_ and M′_4/3_M″_2/3_B_2_ phases (e.g., Mo_4/3_Y_2/3_B_2_, Mo_4/3_Sc_2/3_B_2_), where “M′” covers titanium, chromium, manganese, iron, molybdenum, tungsten, and “M″” mostly covers the inclusion of elements like scandium, yttrium, zirconium, niobium, and hafnium would be probable. The efficiency of each MBenes needs to be carefully evaluated and compared with chiral MXenes of the same category for that bio‐function.

### 3.8 Bio‐Applications of MXenes/MBenes and Prospects for Enhancing Their Bio‐Properties

**Figure 6:**
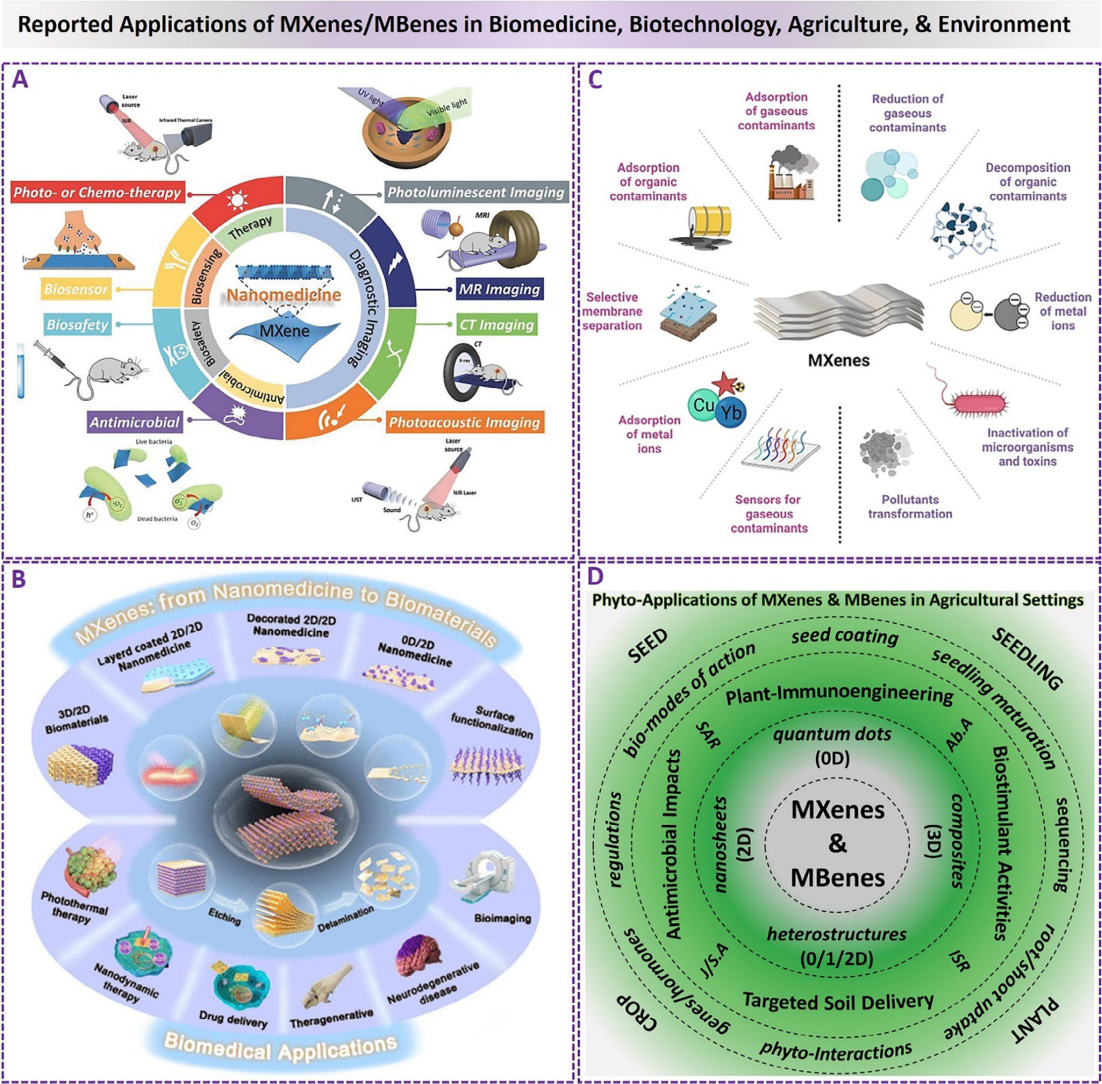
A–D) Representations of the reported applications of MXenes and MBenes in biology, biomedicine, biomedical engineering, bioelectronics, agricultural biostimulation/protection, and the environment. The panels “A–C” are‐produced/merged with permission from graphical abstracts of publications, Open Access/Non‐Open Access Journals, Copyrights form Wiley, ACS, and Springer Nature.^[^ ^42^, ^53^, ^56^ ^]^

To date, significant progress has been made in the bioactivity properties of MXenes/MBenes for theranostic and cancer therapy,^[^ ^40^, ^41^, ^42^, ^43^ ^]^ antimicrobial coatings,^[^ ^44^, ^45^, ^46^, ^47^, ^48^ ^]^ immunosuppression,^[^ ^49^, ^50^, ^51^, ^52^ ^]^ tissue engineering,^[^ ^53^, ^54^, ^55^, ^56^ ^]^ cell and drug delivery,^[^ ^54^, ^57^ ^]^ and bioelectronics and wearable sensors.^[^ ^60^, ^61^, ^62^, ^63^, ^64^ ^]^ As represented in **Figure** **6**, these universal investigations and obtained progress have significantly expanded the scope and boundary of bioactive MXenes/MBenes for future bio‐applications and therapeutic inputs. These studies have reported extensive in vitro, ex vivo, in vivo, and *in planta* models, highlighting the multifunctionality of MXene/MBene‐based biomaterials. Accordingly, the most significant advances and proposed mechanism‐based findings on direct/indirect interactions of MXenes/MBenes with biological systems. Their substantial bioactivities and known underlying mechanisms are pointed out and described accordingly. Furthermore, Figures S6 to S8 (Supporting Information) represent extensive data on diverse bio‐applications of these biomaterials, where the adapted results have been tabulated to compare the properties of each composition.^[^ ^35^, ^36^, ^65^, ^66^ ^]^

#### 3.8.1 MXenes/MBenes and Their Bioactivities for Cellular/Tissue Uptake/Internalization

Over the recent years, there have been several reports that specific MXenes/MBenes at certain doses have the intrinsic capability to be selectively taken up by different types of cell and tissue clusters in diverse living biological systems.^[^ ^67^, ^68^, ^69^ ^]^ According to these reports and their proposed mechanisms, MXenes/MBenes, due to their shape and surface properties can enter cells primarily through clathrin‐mediated endocytosis or other possible mechanisms. This uptake mechanism is a well‐understood biological process, where the cell membranes invaginate and form a vesicle around the biocompatible and bioactive substance/materials like MXenes. Indeed, the large‐area surface, sharp edges, and high hydrophilicity nature of MXenes can physically breach the cell membrane in a smart/gentle way, leading to their entry. Indeed, the behavior of MXenes with pathogens like bacteria and viruses is different from normal cells in terms of membrane disruption/damage or cellular localization for specific bioactivity functions.

In addition, surface modifications or post‐functionalization of MXenes/MBenes with highly biocompatible natural compounds, synthetic substances, or stable bioactive oxides to act as a thin layer around the MXene/MBene nanosheets or quantum dots, modifying their biocompatibilities to a higher level. MXene/MBenes, especially when surface‐modified with bioactive polymeric such as polyethylene glycol (PEG), have been reported to form as forming a bio‐film‐like protein corona, which can be readily and spontaneously taken up by selective cells through endocytosis.^[^ ^67^ ^]^ This is one of the primary cellular processes where bioactive substances are transported into the cells by invading their cell membranes without any specific enhanced uptake techniques. The clathrin‐mediated endocytosis mechanism involves clathrin, a protein that helps form the vesicle. In certain and appropriate cell‐nano interactions, the cultured MXenes/MBenes in a colloidal dispersion system can potentially adsorb a large amount of cell/blood proteins to form a protein corona, which positively influences their biocompatible interactions and cellular uptake and localization in the cytoplasmic area and in/around the nucleus. This proposed endosome‐scape or other mechanisms for MXenes/MBenes internalization into specific cells has also reported to be reversible, and the uptaken particles can be either biodegrade or exit from the cell components, allowing the material to be extracted from cells/tissues, surrounding areas, and or organs through bio‐clearance processes.

#### 3.8.2 MXenes/MBenes and Their Antioxidant/Reactive Oxygen Spices Scavenging Activities

Reactive oxygen species (ROS) are unstable molecules that, at high‐intensity exposures and interactions, can cause significant cellular damage, apoptosis, and subsequent inflammation under extreme conditions.^[^ ^70^, ^71^, ^72^ ^]^ Bioactive nanomaterials have been reported to possess the capacity to modulate ROS generation/production in different living bio‐systems. As for the particular case of MXenes, it has been reported widely that they can readily and effectively smartly regulate ROS levels. In case of cellular/tissue damage, specific MXene biomaterials can act as an active nano‐agent for ROS scavenging by reacting with the ROS molecules (e.g., hydroxyl radicals, superoxide anions, and hydrogen peroxide) through their surface functional groups. By inducing this reaction and its underlying electron donation mechanism, bioactive MXenes act as redox catalyst agents and alleviate or minimize the ROS levels, reducing the activity/intensity of these harmful ROS species and preventing oxidative damage to cellular structures, including DNA structures, proteins, lipids, etc. Certain ROS molecules may induce adverse biological responses such as cell membrane peroxidation, genetic instability, and mitochondrial dysfunction. Indeed, MXenes are reported to reduce these excessive ROS effects, which may contribute to mitigating or preventing oxidative stress‐related diseases such as cardiovascular disorders neurodegeneration, and fast aging.

Specific MXenes are also reported to offer antimicrobial and/or anticancer properties through the generation of ROS to pathogenic organisms or cancer cells,^[^ ^73^, ^74^, ^75^ ^]^ leading to decreasing or preventing their proliferation and cell death. Besides, it has been reported in multiple recent studies, including our previous works, that surface‐modified MXene nanosheets and derived quantum dots have the intrinsic ability to interact positively with living plants and as a prompt response at early stages of interaction to efficiently enhance their alertness and resistance, and stress resilience by inducing plant to produce more ROS eliciting responses. This elicitation can significantly improve the defense response and underlying mechanisms in plants. Further, plants treated with MXene can be primed for better protection against more elongated stresses and adaptability to future attacks by invasive organisms. This dual switchable ROS production/scavenging is highly beneficial.

#### 3.8.3 MXenes and Anti‐Inflammatory/Immunosuppressive Mechanisms via Enhanced Macrophage Polarization, Cytokine Regulation, and T‐Regulatory Cells

MXenes, under certain conditions and concentrations, have been shown to possess the intrinsic capability for modulating macrophage polarization by influencing different pro‐inflammatory inactivation and anti‐inflammatory activation cytokine pathways.^[^ ^76^, ^77^, ^78^ ^]^ In particular, classical activation (M1), which is a response to a stimulant such as lipopolysaccharides, macrophages are typically polarized toward the M1‐phenotype, subsequently causing the production of various pro‐inflammatory cytokines such as tumor necrosis factor‐alpha (TNF‐α), interleukin‐6 (IL‐6), and interleukin‐1 beta (IL‐1β), as well as excessive ROS generation, which together can promote the inflammation progression. Besides, through alternative activation (M2), MXenes have shown the potential to enhance the M2‐macrophage phenotype, which can subsequently lead to the activation/enhanced production of anti‐inflammatory cytokines, such as transforming growth factor‐beta (TGF‐β) and IL‐10. Moreover, specific compositions of MXenes have also been shown to modulate T‐regulatory cells and their underlying functions.^[^ ^77^, ^78^ ^]^ These mechanisms can reduce inflammation and eventually support tissue repair/regeneration. This mechanism of action has been proposed through molecular signaling pathways, including nuclear factor‐kappa B (NF‐kB) and the Janus kinase–signal transducer and activator of transcription (JAK/STAT)‐related pathways. In particular, bioactive MXenes likely reduce the NF‐kB‐based signaling, contributing to inhibiting this pathway, activating M1 macrophages, and reducing the secretion of pro‐inflammatory cytokines.^[^ ^76^ ^]^ Further, through enhancing the JAK‐STAT‐related signaling, specific MXenes potentially contribute to this pathway, shifting to M2 macrophage activation, leading to further anti‐inflammatory responses for inflammation suppression and tissue remodeling. The regulation of M1 macrophage phenotypes in these MXene‐assisted processes has been proposed through distinct physical, molecular, and immunosuppressive pathways, such as ROS modulation, mitogen‐activated protein kinase (MAPK) signaling, and cytokine release.^[^ ^79^ ^]^ MXenes interact with these immunomodulatory pathways, enhancing tissue regeneration and wound healing, and reducing chronic inflammation with the effective ability to improve cancer therapies.

#### 3.8.4 MXene and Angiogenesis Impact on Blood Vessel Regeneration and Improved Antigen Presentation

Through biocompatible and bioactivity interactions with endothelial cells, certain MXenes have also been reported to promote endothelial cell migration, tube formation, and proliferation, which is an important factor for angiogenesis and preventing transplant rejections.^[^ ^78^, ^80^, ^81^, ^82^ ^]^ In particular, vascular endothelial growth factor (VEGF) and its specific receptors, including VEGFR‐1 and VEGFR‐2, have a key role in the proliferation process of endothelial cells for blood vessel formation. MXenes of specific compositions and concentrations are taken up by antigen‐presenting endothelial cells in a biocompatible manner and enhance their proliferation and associated biological mechanisms, influencing surface receptor expression and cell adhesion. Together with their bioactive interaction with endothelial cells, antigen presentation mitigation, and immunosuppressive properties, these MXenes have proven effective in reducing inflammation, tissue infiltration, and damage, mitigating the rejection of transplanted cardiac allografts. This multifunctional bioactivity of MXenes is highly beneficial in their biomedical applications, such as in tissue engineering, regeneration, and transplantation.

#### 3.8.5 MXenes/MBenes and Their Antimicrobial and Anticancer Bioactivities

The antibacterial activities of MXene have been reported to be mostly through physical disruption of bacterial membranes. Indeed, upon the interaction of bioactive MXenes with bacteria, their unique negatively charged and functional surface properties and sharp edges (especially MXene nanosheets) can cause effective mechanical damage to the membranes/cytoplasmic components of the bacteria. MXenes have been proposed to penetrate into the bacterial cell wall, leading to membrane disruption, leakage of intracellular contents, and eventually cell death and presentation of their spreading/proliferation activities. Furthermore, MXenes/MBenes have shown potential for photothermal therapy (PTT) by absorbing near‐infrared (NIR) lights and converting them into heat as a localized temperature that can kill bacteria via thermal damage, and along with generating ROS, causing oxidative damage to the bacteria and cancer cells/tumor‐environments for improved photodynamic therapy (PDT) and advanced combinational chemotherapeutics.^[^ ^40^, ^41^, ^42^, ^43^, ^44^, ^45^, ^46^, ^47^ ^]^

#### 3.8.6 MXenes and Their Applications for Targeted Drug/Gene/Cell Delivery

As efficient nano‐carrier agents, MXenes have also been shown potential for encapsulating therapeutic agents such as small interfering RNA, DNA, and drugs within their layered or particle structures (surface binding, core–shell system, surface coating, or conjugation). The modified MXenes, due to their enhanced surface properties, can then be more efficiently internalized into cells through endocytosis and endosomal escape mechanisms under certain conditions and pHs, facilitating the release of their therapeutic bio‐payload into the targeted cytoplasmic or tissue areas. When modified MXenes are loaded with chemotherapeutic agents, the external chiral layers (e.g., amino acid shells) may stabilize the loaded drugs and facilitate a pH‐/enzyme‐responsive release inside tumors more effectively.^[^ ^43^, ^57^, ^58^, ^59^, ^83^ ^]^ This consequently alters their uptake for enhanced delivery.

#### 3.8.7 Key Considerations for Improving the Bio‐Properties and Applications of MXenes/MBenes

Despite all of these advances and knowledge progress obtained in multifunctional properties and bio‐related applications of MXenes and MBenes, their practices in real‐world approaches are still in the early stages and require detailed mechanistic clarities, understanding their exact modes of action, and long‐term biocompatibility and safety efficacy aspects. These studies are both time‐ and cost‐consuming approaches, and rationale strategies for improving their material stability, biocompatibility, and bioactivity properties to higher levels at the forefront of the field. Given that chirality is a pivotal natural property of many biological systems, and also as we elucidated on enhanced long‐term biocompatibility behavior of chiral MXene in different living plant models, it is highly anticipated that these chiral MXenes and other potential new chiral MXenes offer higher levels of biocompatibility and bioactivity compared to original MXenes. This rationale highlights the importance of chiral MXenes in advancing multiple biological/immunological/electrochemical mechanisms such as biostimulation, cellular uptake, gene expression, immunomodulation, tissue regeneration, ionic charge/transfer, and surface interactions. Chiral MXenes have a vast potential to provide further improvements in the bio‐applications of MXenes, bringing this nanotechnology closer to practical applications in the clinic, translational medicine, agriculture, and the environment. Also, the rationally‐synthesized MBenes, which are indeed the boron analogs of MXenes, may undergo a similar or competitive trend of bioactivity properties, which needs to be investigated through mechanistic studies. In the following sections, we have further discussed these possibilities for each application, highlighting the significant capacity of chiral engineering of MXenes/MBenes.

### 3.9 Application of Design of Experiments (DOE) and Mathematical/Machine Learning/AI‐Based Methods in Predicting/Optimizing Chiral MXenes/MBenes’ Properties

Beyond optimizing the synthesis parameters of MXene/MBene synthesis, particularly of those bottom–up methods, including etchant concentrations, etching time, stirring rate, and temperature, and hydrothermal settings, as well as post‐functionalization processes, including the concentration of additives or intercalants and physicochemical delamination, the preparation design of chiral‐engineered MXenes/MBenes can also be predicted, optimized, and/or maximized using mathematical, machine learning, and AI‐based methods. This rationale can be considered in three different categories. The first one covers applying standard design of experiments (DOE) and Plackett–Burman Design and/or Taguchi optimization to design experimental setups and reduce the trials based on suggested algorithm to minimum possible experiments when three or more parameters are indeed effective (e.g., CMF1‐8: five individual parameters of 2 variable for each, L3*3: Taguchi's L9 orthogonal array (9 selections over 27 possible experiments), L4*4: L16 trials over 64 possibilities). This pre‐filtering can give directions toward setting initial experiments and reasonably reducing the variable possibilities (e.g., 27 to 9 or 64 to 16) in a standard manner.

Accordingly, the obtained outputs based on the DOE as‐selected experiments the inputs can be formulated through mathematical calculations (e.g., signal‐to‐noise Ratio) to find the maximum outputs at that level and further reduce the experiments for validation or re‐experimentation with the new suggested parameters. This step itself can effectively optimize the preparation processes. However, the application of machine learning and AI‐based algorithms, such as adaptive neuro‐fuzzy inference systems, artificial neural network (ANN), support vector machines (SVMs), gene expression programming (GEP), and multi‐objective particle swarm optimization (MO‐PSO), can also be implemented for further optimization of the synthesis parameters or maximizing the outputs beyond the basic DEO and mathematical approaches. It is noteworthy that implementing these strategies alongside DFT‐based calculations can give detailed optimization and predictions of the physicochemical and biological properties of chiral MXenes/MBenes. Moreover, by appropriate testing–training systems within the performed computations, novel properties of developed chiral MXenes and not‐yet‐constructed MXene/MBene‐based biomaterials can be systemically predicted and investigated. Lastly, due to the importance of short‐, mid‐, and long‐term biocompatibility of MXenes/MBene nanomaterials for their practical applications and also the nature of these research practices, which is both time‐consuming and costly, such robust modeling is highly recommended for further developments in bio‐related and therapeutics domains of nanotechnology. Below, some of the effective parameters for chiral optimizations of MXenes/MBenes are listed:

- Concentration of MXenes/MBenes in their initial colloidal dispersions and crosslinking agents
- Molarity of citric acid or other compounds for carboxyl‐functionalization of MXenes/MBenes
- Duration and type of the applied sonication treatment (probe/bath‐ultrasonic)
- Left‐/right‐structure and the type of chiral ligands (e.g., l‐/d‐handed amino acids)
- Concentration/effectiveness of the used amino acid or other natural/synthetic chiral sources
- Time, stirring speed, and temperature of the applied functionalization/hybridization treatments

### 3.10 Proposed Predictions for Biocompatibility of Chiral MXenes/MBenes with Biosystems

As discussed, except for the left‐ or right‐handed chiral Ti_3_C_2_T_x_ MXenes in our previous work, no other chiral MXenes/MBenes have been reported. We observed a high potential of these chiral MXenes in terms of long‐term biocompatibility with different plant‐based models.1 In the current section, we strived to provide reasonable recommendations for prioritizing the synthesis of chiral MXenes/MBenes based on their initial compatibility assessments with different biological systems. It is important to mention here that beyond the biocompatibility enchantment of MXenes or MBenes, improving their stability and bioactivity properties is also a key aim of chiral engineering. Thus, a reasonable prioritizing for the construction of new chiral MXenes/MBenes must be considered multifactorial and based on the previous reports of biocompatibility and biofunction of that particular composition/form for the targeted applications.

Based on the available literature on the biocompatibility/toxicity of original MXenes/MBenes, we can estimate in vitro, in vivo, and *in‐planta* biocompatibility profiles of chiral MXenes/MBenes. This estimation relies on two theoretical concepts. First, tailoring their microstructures with chiral‐active substances does not impose toxicity effects. Rather, the biocompatibility of products is expected to be enhanced by chiral engineering due to the addition of bioactive molecules to the surface of chiral MXenes/MBenes. However, these claims need to be further validated. Due to the lack of sufficient literature to comment on the biocompatibility of chiral MXenes/MBenes, we predicted an initial toxicity of the proposed materials based on their nearest chemical compositions.

For nano‐toxicity prediction using predictor tools, such as the Pro‐Tox‐3.0 chemical predictor, a standard chemical formula or the material's SMILES number (simplified molecular input line entry system) is required. These numbers are available in the PubChem Online Database for different types of materials. However, few limited structures have been reported so far for MXenes/MBenes (only niobium carbide and molybdenum carbide MXenes). In this perspective, using Pro‐Tox‐3.0, we predicted the biocompatibility/toxicity of the available MXenes/MBenes and their most similar compositions.^[^ ^84^ ^]^ As shown in Figures S9 to S49 (Supporting Information), the radar plots predicted an initial chemistry‐dependent biocompatibility or toxicity prediction evaluation of these materials and representative chiral‐active substances. These predictions suggest that they are not generally categorized as toxic compounds (at least in short‐term interactions or moderate‐period exposures and defined dose thresholds). However, the precautionary considerations of these materials have been predicted for a limited number of bio‐applications. This encompasses potential adverse impacts on the blood–brain barrier (BBB), neurotoxicity, and eco‐toxicity. It should be noted that several publications have experimentally shown a high biocompatibility of MXenes/MBenes with neuron cells and specific ecological systems, such as research‐scale soil media. Thereby, the predicted toxicity may not be sufficient for robust evaluation, and further experimental studies are required to fairly comment on these predictions. However, the useful information provided by these predictions, alongside the previously reported bioactivity evaluations of MXenes/MBenes, can be used as an initial guide for prioritizing the synthesis and optimization of their chiral‐modified forms. Obviously, the properties of each new chiral‐modified material need to be carefully compared with its original composition for robust validation of enhanced properties.

#### 3.10.1 The Proposed Hypothesis for Prioritizing the Bio‐Applications of Chiral MXenes/MBenes

The next aim of this perspective is to rationally hypothesize on the bioactivity properties of chiral‐engineered MXenes/MBenes. By comparing the functional efficiency of original materials for diverse bio‐applications and acknowledging their potential to address longstanding challenges in these fields, it is highly expected that chirality plays a significant role. As experimental and computational evidence, we refer to the multifunctional bioactivities of chiral‐modified Ti_3_C_2_T_x_ MXenes for plant protection and biostimulation.^[^ ^1^ ^]^ Thereby, we speculated that by inducing stable chirality in the structure of MXenes/MBenes, their bioactivity can be enhanced for antimicrobial and cancer therapies, drug delivery, immune modulation, tissue engineering, and agricultural uses.

Given the importance of these aspects and long‐term biocompatibility requirements of synthetic nanomaterials for practical uses, developing rational strategies for maximizing bio‐properties of fascinating MXenes/MBenes is among the current focuses of the field. Chirality induction has held great promise, and chiral MXenes/MBenes have the potential to significantly reduce concerns regarding their nano‐toxicity and probable long‐term adverse immunological effects. The ultimate aim is to prioritize the construction of highly biocompatible and bioactive chiral MXene/MBene compositions. Based on these considerations, we strived to speculate on some of these aspects in **Table** **1**. In particular, we provided recommendations for prioritizing the most preferred compositions of MXenes/MBenes based on their expected surface chemistry, mechanical and electroconductivity properties, and anticipated bioactivities for specific bio‐related applications.

**Table 1:** The proposed preferability and speculated priority levels of chiral MXenes/MBenes. This table has been envisioned to reflect our recommendation for prioritizing the development of new chiral‐modified MXenes/MBenes for diverse bio‐related applications.

Chiral‐active MXenes / MBenes	Drug delivery/release	Antimicrobial bioactivities	Cancer therapy/diagnose	Immune modulation/bio‐sensing	Tissue engineering	Plant bio‐stimulation
Titanium carbide‐based MXenes	High priority	Moderate priority	Moderate priority	High priority	High priority	High priority
Zirconium/Hf carbide‐based MXenes	High priority	Moderate priority	Moderate priority	Moderate to low priority	High priority	Low priority
Niobium carbide‐based MXenes	High priority	Moderate priority	High priority	Moderate to high priority	High priority	High priority
Tantalum carbide‐based MXenes	Moderate priority	Low to moderate priority	High priority	Moderate to high priority	Moderate priority	Moderate to high priority
Molybdenum carbide‐based MXenes	Moderate priority	High priority	High priority	Moderate to high priority	High priority	Moderate to high priority
Vanadium carbide‐based MXenes	Moderate priority	High priority	Moderate priority	High priority	Moderate priority	High priority
Chromium carbide‐based MXenes	Low priority	Moderate priority	Low priority	Low priority	Low priority	Low priority
Titanium nitride‐based MXenes	Moderate priority	Low priority	Low priority	Moderate priority	Moderate priority	Low priority
Molybdenum carbide/nitride‐based MXenes	Moderate priority	Low priority	Moderate priority	Moderate priority	Low priority	Low priority
Boron carbide‐based MBenes	Moderate priority	High priority	Moderate priority	Low priority	Low priority	High priority
Boron nitride‐based MBenes	High priority	High priority	Moderate priority	Moderate priority	High priority	Low priority
Carbonitride Boron MBenes	High priority	Moderate to high priority	Moderate priority	Low to moderate	Low priority	Moderate priority

In particular, due to the biological characteristics of chiral‐active compounds, several biological or immunological properties of the original MXenes/MBenes are assumed to be improved. This theoretical concept relies on the potential influence of chiral‐active substances on biological systems. For instance, chiral surfaces have a high tendency to bind to stereospecific cell receptors, which might subsequently lead to enhanced delivery and bio‐release properties. Additionally, chiral‐active materials may more efficiently insert into the chiral lipid bilayers of pathogens and other microorganisms.

Chiral‐active surfaces may also better selectively bind to different immune cells and their associated receptors for enhanced immunomodulation. The chiral engineered materials may also offer enhanced bioactivity properties to mimic natural extracellular matrix, guiding stem cell fate for promoting the efficacy of cell therapies and tissue regeneration. Further, chiral‐modified biomaterials may benefit from the relatively higher regulation of ion channel activities and neurotransmitter binding, which can contribute to increasing neuron tissue engineering, maturation, as well as the efficiency of bioelectronics and stimulators. Chiral‐active nanomaterials likely better recognize/quantify enantiomers in specific biosystems, suggesting their biosensing potential. With respect to agricultural plant protection, in addition to the fascinating already reported bio‐properties of MXenes/MBenes, the induction of chirality may increase their capacity to mimic phytopathogen motifs, more effectively activating defense pathways and stress resistance. It might also better interact with the plant's cellular compounds, enzymes, and hormones, increasing the expression of defense‐related genes and other signaling pathways. It is expected that chiral MXenes/MBenes may potentially offer enhanced biocompatibility and bioactivity compared to their non‐chiral compositions, which need to be carefully evaluated through mechanistic studies.

#### 3.10.2 Hypothesizes on the Role of Chirality on Biological/Immunological Properties of MXenes/MBenes

Fundamentally, the induction of chirality into bioactive nanomaterials enables the design of next‐generation materials. Chiral‐engineered biomaterials can interact efficiently with biological systems, using a stereochemical language the same as, or akin to, that used by life's molecular machinery. Such nano‐enabled artificial bio‐communications create new recognition, surface‐binding, signaling, and functional mechanisms in biosystems. Transforming these properties into synthetic nanomaterials can significantly improve their biological applications. In addition to the inherent active surface areas and unique physicochemical properties of low‐dimensional biomaterials, the induced chirality enhances their stability, cellular interactions, and bioactivity in a relative manner. More importantly, it improves their short‐ and long‐term biocompatibility, which is crucial for their future translations in real‐world bio‐applications.

Despite the scientific merits behind the proposed hypotheses, detailed computational and experimental validations are required for each chiral MXene/MBene. This validation can robustly comment on their enhanced biocompatibility and bioactivity properties compared to their parent and other bioactive similar nanomaterials. The speculations on how the chirality induction may effectively improve the bio‐functionality of MXenes/MBenes are accordingly described for each application:

#### 3.10.3 Proposed Pathogenic Interactions and Antimicrobial Actions of Chiral‐Modified MXenes/MBenes

It is known that the cell wall components of bacteria and surface proteins of viruses are inherently chiral or contain chiral‐active molecules. In particular, peptidoglycan with d‐amino acids and/or chiral teichoic acids naturally exists in the structure of bacteria. In the case of viruses, viral proteins, particularly those that are involved in the capsid, can possess chiral structures. Additionally, several components of fungi spontaneously exhibit chirality, including sugars (e.g., glucose, mannose) within their cell walls and cytoplasm areas, as well as specific amino acids and lipids that can form chirality in their glycerol backbone or within fatty acid chains. Upon interaction/exposure with pathogenic microorganisms, given chiral nanomaterials are able to further disrupt their survival, growth, and proliferation activities with enantioselectivity. For instance, l‐chiral MXenes/MBenes may more efficiently insert into the pathogens’ membranes, increasing permeability and causing cell rupture (lysis) in bacteria and cell damage (disintegration or breakdown) in the other microbe types. In addition, the induced chiral surfaces can potentially inhibit the assembly of biofilms by stereo‐selectively binding to the proteins’ matrix or polysaccharides. Thus, through reduced resistance mechanisms, the stereoselective process makes it more challenging for bacteria to adapt and increase their populations, contributing to enhancing the antimicrobial impact of bioactive nanomaterials.

#### 3.10.4 Proposed Immune Modulation/Suppression Mechanisms of Chiral‐Modified MXenes/MBenes

Considering the inherent immunomodulatory properties of MXenes/MBenes, the induction of chirality might further impact the regulation of immune cells and associated responses. In particular, the immune receptors such as T‐cell receptors, Toll‐like receptors, and antibodies contain chiral proteins with specific stereochemistry. Chiral‐active nanomaterials, regardless of whether their chirality is innate or induced, can fundamentally interact better with these receptors through different affinities. For instance, l‐handed chiral nanomaterials can potently trigger higher cytokine production compared to d‐handed achiral analogs. In addition, pathogen‐associated molecular patterns (PAMPs) are often chiral or contain chiral compounds, such as d‐handed amino acids available in the cell walls of bacteria are efficiently recognized as “non‐self” by innate immunity.^[^ ^85^ ^]^

#### 3.10.5 Proposed Anticancer Properties and Metabolomic Programing of Chiral‐Active MXenes/MBenes

With respect to the anticancer properties of MXenes/MBenes, the induction of chirality may positively impact by regulation of cancer cells and subsequent cell death mechanisms and tumor ablation. In particular, since tumor cell surface proteins and their extracellular matrix components are chiral or contain chiral biomolecules. Treatment of cancer cells with chiral MXenes/MBenes can increase the enantioselective binding to overexpressed receptors, resulting in higher tumor uptake for both direct effect and targeted drug/light delivery. Furthermore, specific chiral‐engineered materials may promote the production of oxidative stress in cancer cells, which may result in further leading to apoptosis and cancer cell death.

Moreover, the chirality properties of nanomaterials can directly influence their biological effects and transport in underlying physiological conditions. One of these bioactivity properties is cellular metabolic reprogramming, which is indirectly correlated with the immune system and immunological pathways. For instance, it was reported that as‐synthesized chiral gold‐based particles exhibit superior capability of activating macrophages compared to non‐chiral gold particles, facilitating the upregulation of the expression level of creatine kinase muscle‐type (CKM).^[^ ^86^ ^]^ Furthermore, this macrophage activation could be mediated through the activation of the NF‐κB and NLRP3 inflammasome pathways. Moreover, it is reported elsewhere that chiral‐active nanozymes can be used as highly efficient localized reactive oxygen species generators, which is beneficial in central therapy to reprogram tumor‐associated macrophages (TAMs) by harnessing their chirality‐dependent interactions with biosystems.^[^ ^87^ ^]^ As it is known, extracellular free‐radical ROS, rather than the intracellular type, can play a non‐substitutable role in regulating tumor‐suppressing (M1) and polarization of tumor‐associated macrophages (TAMs). Considering this strategy may enhance the efficacy of macrophage‐based immunotherapeutic approaches. One of these potential nanozymes is the heterostructures of molybdenum disulfide (MoS_2_) and cobalt disulfide (CoS_2_). They reported that the induction of chirality into the structure of these nanozymes could effectively modulate the TAMs polarization and reverse tumor immunosuppression mechanisms. In addition, their results suggested a chiral left or right‐handed‐dependent bioactivity of these materials for cancer immunotherapy, opening up new avenues of research for applications of chiral‐modified nanozymes in immunomodulation and nano‐therapeutic applications of chiral‐engineered MXenes.

#### 3.10.6 Proposed Cell Therapy and Tissue Engineering Bioactivity of Chiral‐Modified MXenes/MBenes

Considering the bioactivity of MXenes/MBenes for cell therapies and tissue engineering, the chirality induction may also positively impact enhancing cellular uptake, survival, differentiation, and maturation of cells and subsequent regeneration mechanisms in translational medicine. In particular, as cell adhesion molecules, cell receptors, proteins, and their extracellular matrix components are chiral or contain chiral biomolecules. Treatment of cells with chiral‐modified MXenes/MBenes can effectively promote selective cell adhesion and spreading, cell survival, and in situ targeted differentiations. Furthermore, specific chiral‐engineered materials are able to bind to specific growth factor receptors, promoting proliferation and tissue growth for regenerative medicine and disease treatment toward more efficient personalized and donor‐based therapeutic strategies.

#### 3.10.7 Proposed Biosensing, Tracking, and Diagnostic Applications of Chiral‐Modified MXenes/MBenes

MXenes/MBenes are known for their inherent optical surface absorption properties and transparency, which can benefit their applications for cell tracking, bioimaging, and related diagnostics. Chirality induction may positively impact. In particular, chiral active nanomaterials (especially, single‐layered colloidal dispersions or quantum dots) have the capability to efficiently recognize and bind specific enantiomers of biomolecules, including amino acid ligands, sugars, and drugs, through stereospecific interactions. Since these materials exhibit circular dichroism and optical rotation, they are able to produce measurable and trackable signals, especially when interacting with specific enantiomers of interest for bio‐applications.

#### 3.10.8 Proposed Plant Immunoengineering/Biostimulation Impacts of Chiral‐Modified MXenes/MBenes

**Figure 7:**
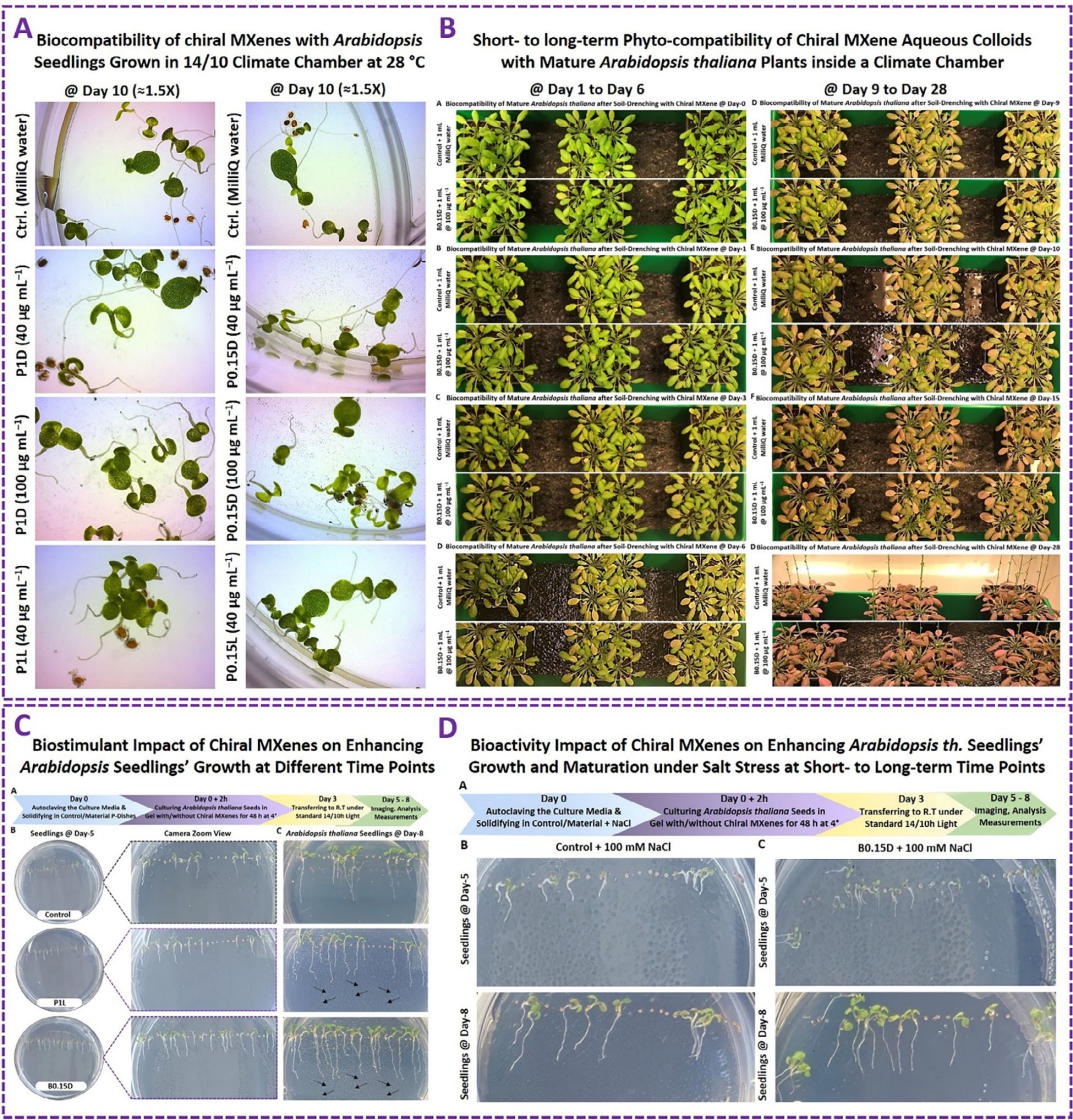
A) Phyto‐compatibility assessment of chiral MXene colloids with *Arabidopsis thaliana* seedlings germination, growth, and maturation. Optical microscopic images (1.5× zoom) depicted the development of chiral MXene‐treated (40 and 100 µg mL^−1)^ and control groups (same amounts of MilliQ water) at day 10 post‐culture in Murashige‐Skoog (MS) media (*n* = 10 to 20 per sample). These observations suggested high biocompatibility of these chiral MXene aqueous colloidal dispersions without any significant microscope‐visible toxicity effects on germination, growth, and root/shoot maturation. B) *In‐planta* short‐ to long‐term biocompatibility of Ti_3_C_2_T_x_‐based chiral MXene aqueous colloids. Phyto‐compatibility assessment of these MXenes at 100 µg mL^−1^ with mature *Arabidopsis thaliana* plants and their qualitative impact on overall growth at different time points (day 0 to day 28) post‐soil‐drenching applied on the plants’ entire leaf/shoot parts (*n*= 3 and each pots represented five individual plants). C) Representation of the experiment timeline and plant biostimulation impact of chiral MXene aqueous colloids at different time points. *Arabidopsis thaliana* seeds were line‐cultured in solidified media with and without the presence of chiral MXenes at 88 µg mL^−1^. The camera images depict the seed germination and root length analysis of these seeds in chiral MXenes‐treated and control groups at different time points, showing a remarkable enhancement in the growth and maturation of these seedlings at day‐8 post‐treatment (*n* = at least 25 per sample). D) Representation of the experiment timeline and biostimulation activity of chiral MXene aqueous colloids under salt stress conditions at different time points. *Arabidopsis thaliana* seeds were line‐cultured in solidified sucrose‐supplemented MS culture media, including 100 mm of water‐dissolved sodium chloride (NaCl) solution with/without chiral MXenes at 88 µg mL^−1^. The camera images depict the seed germination and root length analysis of these seeds in chiral MXenes‐treated and control groups at different time points, showing a remarkable enhancement in the growth and maturation of these seedlings post‐treatment (n = at least 25 per sample). The ImageJ was used to measure the seeds’ roots. Panels are reproduced and merged with permission from our previous work, Copyright, CC Open Access Attribution 4.0 International, Wiley).^[^ ^1^ ^]^

Given that chirality is a pivotal property of plants, contributing to their growth, development, shape, and metabolisms, it is expected that new chiral MXenes/MBenes offer enhanced phyto‐compatibility and activities. According to our data, chiral‐modified MXenes could efficiently promote seed germination, sprouting, seedling maturation, and plant growth/resistance under different abiotic stress conditions.^[^ ^1^ ^]^ **Figure** **7** represents some of these findings. This is a highly important field in nano‐agriculture and has the capacity to push the boundaries of modern farming once the safety of these nanomaterials has been approved.

#### 3.10.9 Key Considerations for Prioritizing the Synthesis of Chiral‐Active MXene/MBene Biomaterials

Taking these accounts into consideration and based on the proposed hypotheses, prioritizing the synthesis and choosing the most preferred chiral sources are key aspects for developing highly efficient chiral MXenes/MBenes.^[^ ^44^, ^88^, ^89^, ^90^, ^91^, ^92^, ^93^, ^94^, ^95^, ^96^, ^97^, ^98^, ^99^, ^100^, ^101^, ^102^, ^103^, ^104^, ^105^, ^106^, ^107^, ^108^ ^]^ Some of these factor‐dependent considerations include:

- Biocompatibility or cytotoxicity: How do the chiral‐engineered MXenes/MBenes interact with different biological systems? This includes the cellular interactions, uptake, surface attachments, viability, tissue integration, and potential immune response activation.
- Surface chemistry and chiral functionalization: Which chiral sources and surface terminations are most suitable? How do these variations influence cell interactions, biomolecule adsorption, and the bio‐applications of the final products?
- Electrical conductivity and mechanical properties: How do the physical and electrical features of chiral MXenes/MBenes—whether synthesized compositions or theoretical phases—affect their multifunctional roles in bioelectronics and tissue engineering?
- Diverse antimicrobial activities: How competitive are new chiral‐active MXenes/MBenes in exhibiting potential against different types of bacteria, viruses, fungi, and phytopathogens? What are the probable harmful effects of chiral MXenes‐MBenes to environmental media (either from production and applications)? And ultimately, how are they comparable in combating infections and treating diseases in biological settings?
- Anticancer and immunomodulation impacts: Which of these MXenes/MBenes can offer more smart and multi‐functional bioactivity features for treating cancer and modulating immune responses (either immune stimulation and immunosuppression activities)?
- Material's stability and processability: Does chiral‐engineering enhance the reactivity and functionality of MXenes/MBenes in bio‐systems, while maintaining their characteristic properties? Which chiral MXenes/MBenes (compositions or forms) outperform in each bio‐application? How are their tuned surfaces of end‐products comparable in terms of intercalation, doping, loading, or release capacity?

#### 3.10.10 Cost Considerations and Economic Aspects for Price Reduction and Large‐Scale Production of MXenes/MBenes

The cost consideration and economic aspects of large‐scale production of MXenes/MBenes, and their proposed chiral engineering, are not the focus of this basic science research perspective. However, in this section, we attempted to briefly discuss some of these points. In this regard, multiple points are important and need to be considered. The first note refers to the significantly newer generation of these 2D materials, compared to the previous nanomaterials. The emerging field of MXenes/MBenes has been rapidly progressing and is expected to accomplish lower‐price and synthesis optimization achievements in the near future. The second point is about the possibility of using recycled precursors for manufacturing the MAX phase materials, which can not only reduce the products’ cost, but is also beneficial in improving climate changes and the environment. Another aspect is the multifunctionality of MXene/MBene biomaterials, as well as their lower working doses compared to other similar nanomaterial types. These highlight the economic satisfaction of MXenes/MBenes, once these nanotechnologies are proven safe for bio‐related applications.

In particular, two recent studies by Zaed et al. have explored the cost of MXene(s) by covering the detailed evaluation of each stage of their synthesis processes.^[^ ^109^, ^110^ ^]^ They elaborated on the cost of each factor involved in the approximate production of MAX‐phase ceramics and their conversion into MXene nanosheets. Their publication reported the cost comparison of each step, highlighting the costliest steps toward further improvement and price reduction. It is mentioned that 90% of the costs are spent on the precursor (34%), thermal treatment (30%), and etching process (26%). They also proposed that the actual costs for human effort expenses and step‐characterizations are 3% and 7%, respectively. The other steps, including ball milling, mixing, washing, drying, grinding, and filtering, are not considered notable costs (see Figure S50 for the adapted data and notes, Supporting Information). According to their analysis and stage evaluations, the overall cost of producing one gram of MXene nanosheets (e.g., Ti_3_C_2_T_x_) is determined by incorporating the prices associated with all the preparation and characterization stages to be ≈20 US$. It is recommended that by optimizing the experimental steps and reducing the main costs, the final prices could be reduced to further satisfy the commercialization aspects. The biocompatibility and safety of the products are still the key requirements for their real‐world applications, especially in the bio‐sectors.

## 4 Conclusion and Outlook

In conclusion, this perspective presented an innovative and insightful perspective as a follow‐up report with our recent work on the first demonstration of fabricating chiral MXene nanosheets, derived quantum dots, and mixed‐dimensional heterostructures. We described most of the available strategies for inducing chirality from different natural and synthetic sources, paving the way for designing and constructing new chiral MXenes and MBenes for diverse bio‐related applications. The proposed reactions and mechanisms are required to be robustly validated by experimental works, computer modeling and calculations, and properties predictions for each MXene/MBene.

This perspective establishes new paradigms for developing hundreds of novel chiral‐active MXenes/MBenes for diverse bio‐applications, where original MXenes/MBenes have demonstrated potential to address longstanding challenges in these fields. The applications of DFT calculations, precise machine learning, and AI‐based approaches are considered future work for modeling, predicting, and optimizing the synthesis parameters and bio‐properties of chiral MXenes/MBenes. It is expected that the induction of chirality using different sources for different compositions/forms of these nanomaterials may face some challenges. If the induction of bioactive chiral molecules and their stable attachments to MXenes/MBenes are not successful, several management risk strategies can be applied. These considerations are not limited to optimizing the chiral dose or replacing it with alternative sources and induction methods. The future direction of chiral engineering of MXenes/MBenes is establishing a new era with numerous prospective research and development opportunities for designing, developing, modifying, improving, and programming next‐generation multifunctional biomaterials.

## Conflict of Interest

The authors declare no conflict of interest.

## Author Contributions

This study was conceptualized, designed, drafted, and reviewed by A.R. and A.A. The authors have approved its submission for journal publication.

## Supporting information



Supporting Information
