# Supplementary material for: Guides for Developing Hundreds of Novel Chiral MXenes and MBenes Nanosheets/Quantum Dots for Next‐Generation Chiral Engineered Biomaterials Applications
Source: Adv Healthc Mater. 2025 Sep 4;14(29):e02422. doi: 10.1002/adhm.202502422 (PMC12616604; doi:10.1002/adhm.202502422)
Supplement: Supplementary file 1 — Supporting Information [file ADHM-14-0-s001.docx]

**Electronic Supplementary Information**

**Guides for Developing Hundreds of Novel Chiral MXenes and MBenes Nanosheets/Quantum Dots for Next-Generation Chiral Engineered Biomaterials Applications**

Alireza Rafieerad^a,b,c,*^, Ahmad Amiri^e,d^

*^a^ Institute for Molecular Biosciences, Johann Wolfgang Goethe Universität, 60438 Frankfurt am Main, Germany*

*^b^ Institute for Biology and Biotechnology of Plants, University of Münster, Schlossplatz 8, 48143 Münster, Germany*

*^c^ Regenerative Medicine Program, Institute of Cardiovascular Sciences, St. Boniface Hospital Research Centre, Department of Physiology and Pathophysiology, Rady Faculty of Health Sciences, University of Manitoba, Winnipeg, Canada*

*^d^ Department of Mechanical Engineering, The University of Tulsa, OK 74104, United States*

*^e^ Russell School of Chemical Engineering, University of Tulsa, Tulsa, OK 74104, United States*

***Content:***

**Supplementary Figures S1** to **S50** (52 pages).

***Correspondence:**

Alireza Rafieerad, MSc, PhD

^1^ Institute of Molecular Biosciences, Faculty of Biological Sciences, Goethe University, 60438, Frankfurt am Main, Germany

^2^ Institute for Biology and Biotechnology of Plants, Münster University, Schlossplatz 8, Germany

^3^ Advanced Biomaterials, Nano-Immune Engineering, and Regenerative Nano-Medicine Program

Canada-Italy Tissue Engineering Laboratory (CITEL), ICS, Saint Boniface Albrechtsen Research, Rady Faculty of Health Sciences, University of Manitoba, Winnipeg, R2H 2A6, Manitoba, Canada

Contact E-mail: [alireza.rafieerad.formal@gmail.com](mailto:alireza.rafieerad.formal@gmail.com)

**Supplementary Figures S1** to **S4:**

Representation of the summary of *in-vitro and in-vivo* biocompatibility/toxicity of MXenes.

**Supplementary Figures S5**:

Representative illustration of MXenes/MBenes of different chemical compositions towards developing chiral nanosheets/quantum dots biomaterials using distinct chiral-active source.

**Supplementary Figures S6** to **S8:**

Representation of the summary of *in-vitro and in-vivo* bio-applications of MXenes.

**Supplementary Figures S9 to S49:**

Representation of the predicted biocompatibility/toxicity of MXenes and similar chemical stricture with diverse biological and environmental systems.

**Supplementary Figures S50:**

Representative illustration of recently-reported cost consideration of MXenes towards their price reduction and large-scale production.


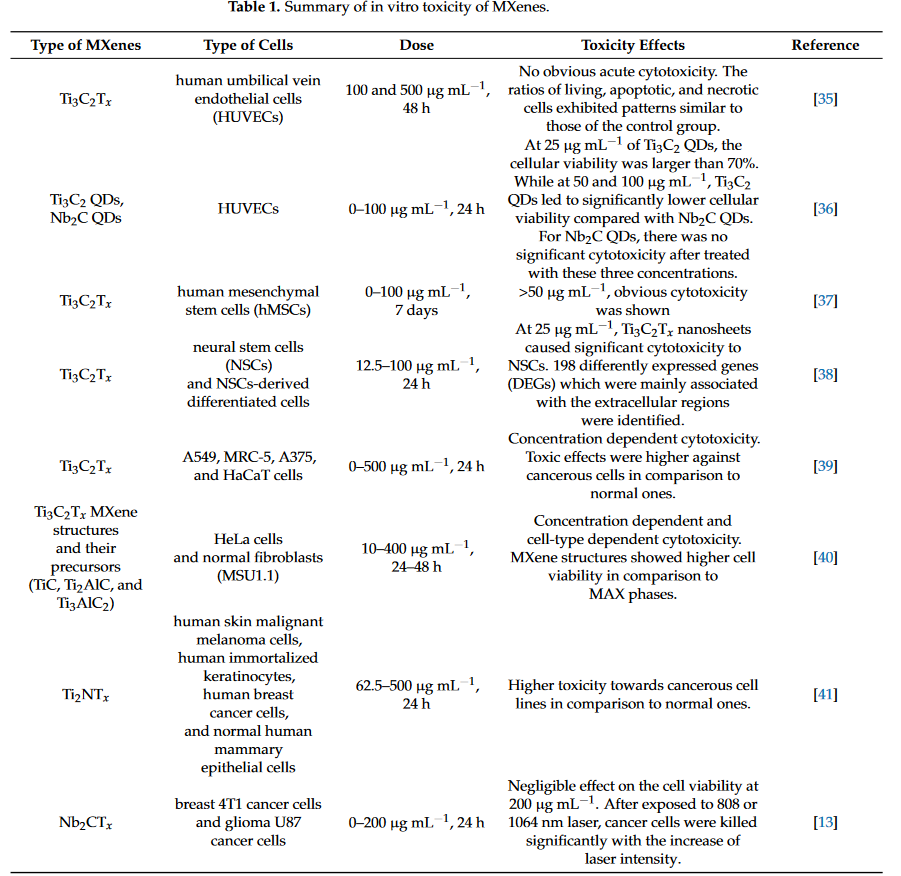


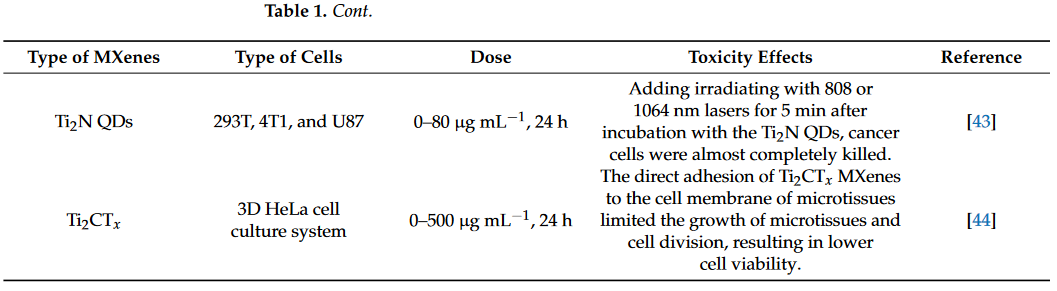


**Supplementary Figure S1:** The summary of *in-vitro* biocompatibility/toxicity of MXenes. This information is adapted from Table 1 in Wu et al. (2022) with permission from *open access Creative Common CC BY license Copyright, MDPI Publisher*.^https://doi.org/10.3390/nano12050828^


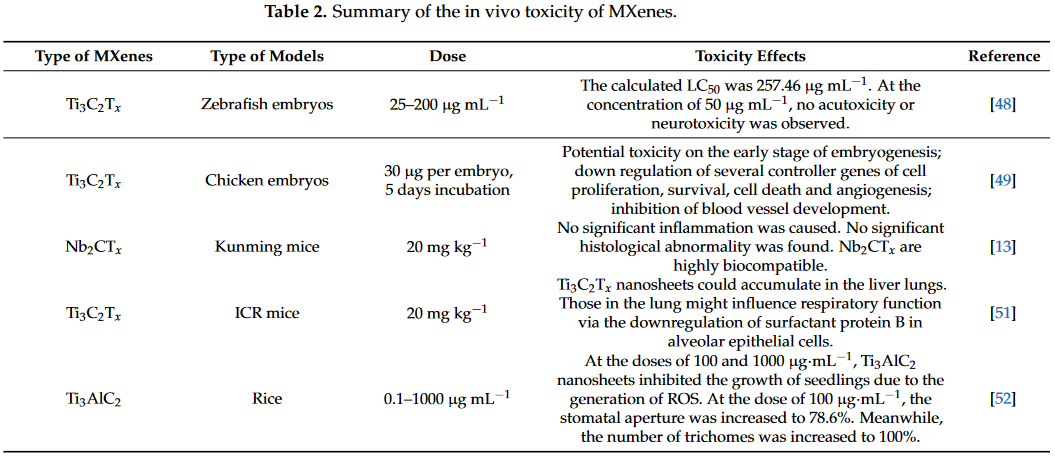


**Supplementary Figure S2:** The summary of *in-vivo* biocompatibility/toxicity of MXenes. This information is adapted from Table 2 in Wu et al. (2022) with permission from *open access Creative Common CC BY license Copyright, MDPI Publisher*.^https://doi.org/10.3390/nano12050828^


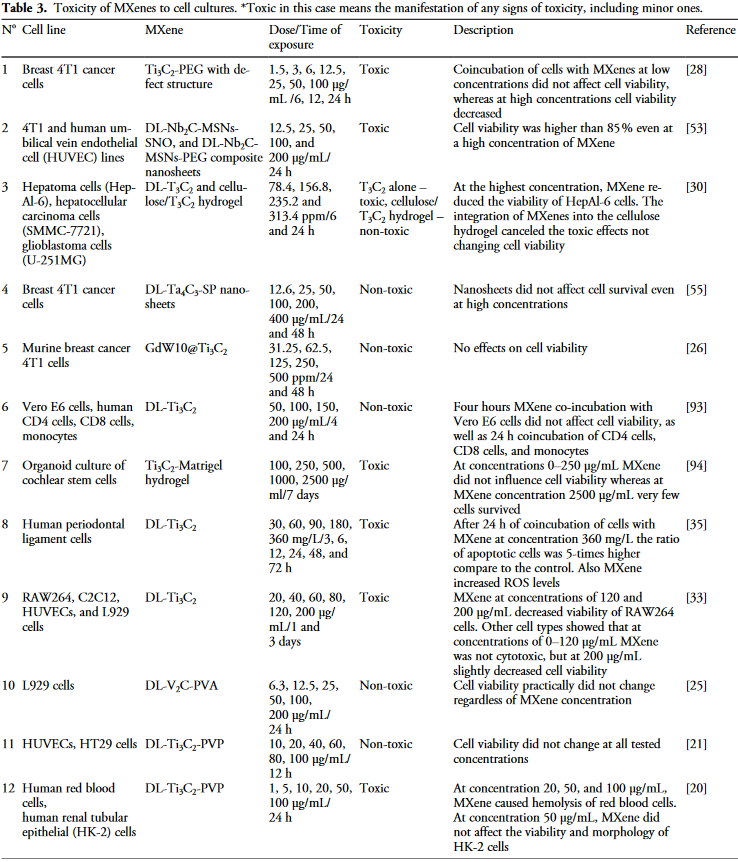


**Supplementary Figure S3:** The summary of biocompatibility/toxicity of MXenes to cell cultures. This information is adapted from Table 3 in Dmytriv et al. (2024) with permission from*, The Chemical Records, Wiley Publisher*.^https://doi.org/10.1002/tcr.202300338^


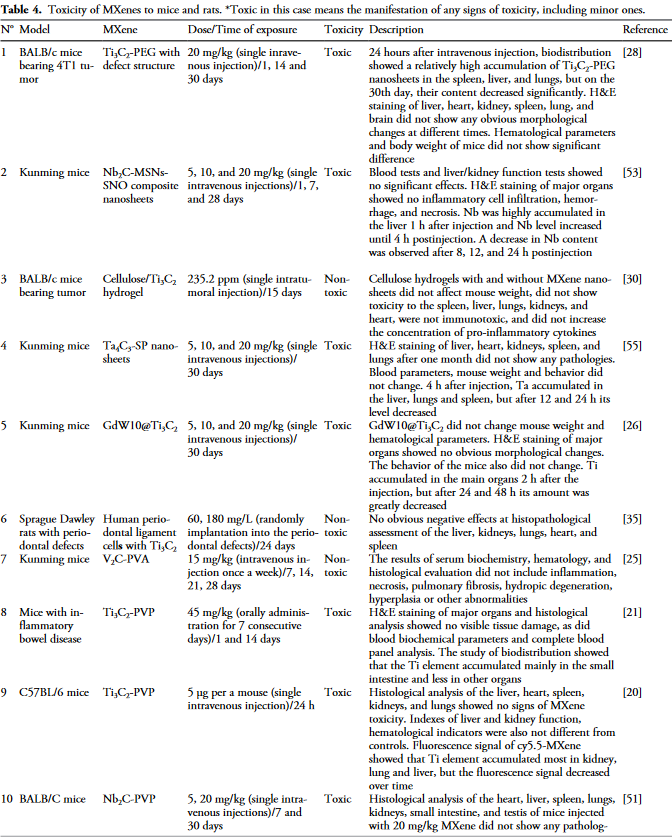


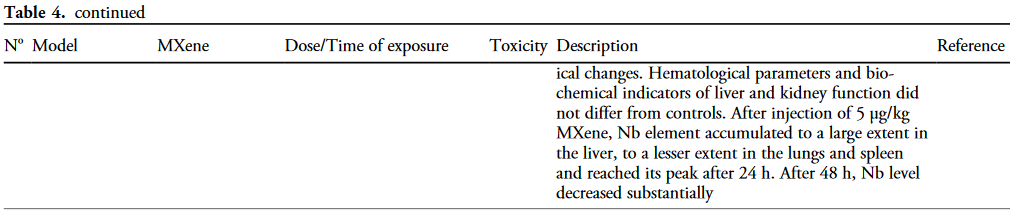


**Supplementary Figure S4:** The summary of biocompatibility/toxicity of MXenes to cell cultures. This information is adapted from Table 4 in Dmytriv et al. (2024) with permission from*, The Chemical Records, Wiley Publisher*.^https://doi.org/10.1002/tcr.202300338^


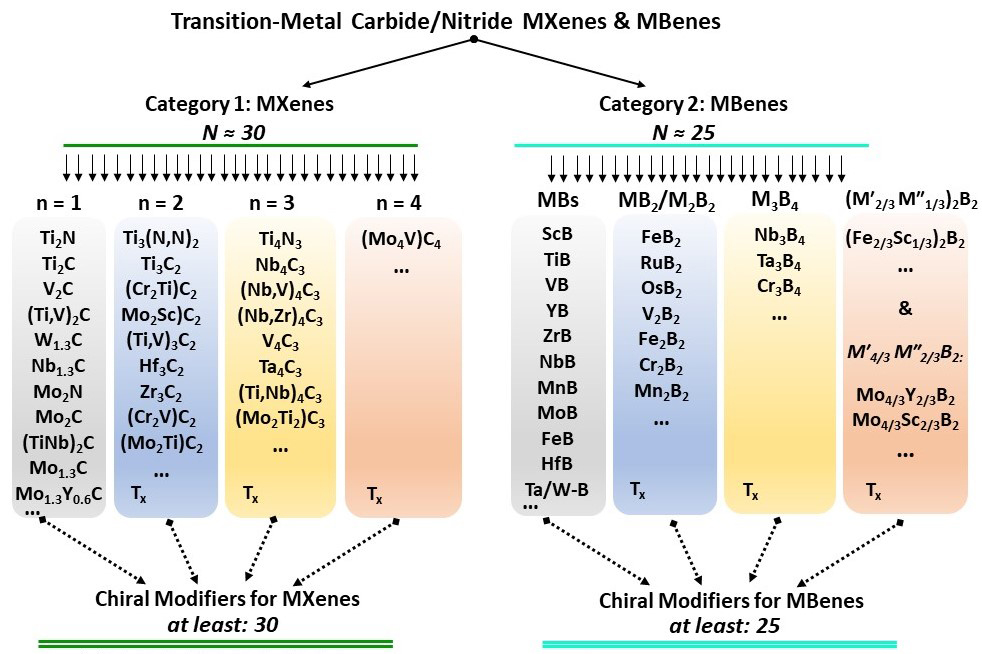


**Supplementary Figure S5:** A representative illustration of MXenes and MBenes of different chemical compositions towards developing chiral nanosheets/quantum dots biomaterials using distinct chiral-active sources.


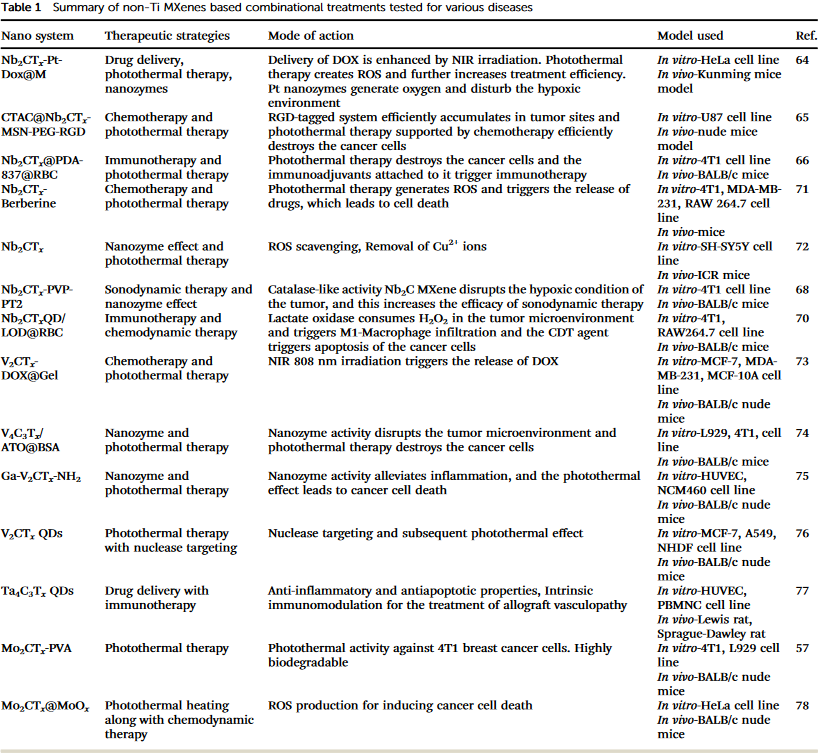


**Supplementary Figure S6:** The summary of recent research on non-MXene-based nanomaterials for treatment of various diseases. This information is adapted from Table 1 in Gayathri et al. (2024) with permission from*, open access copyright license, Journal of Materials Chemistry B, RSC Publisher*.^https://doi.org/10.1039/D4TB01904K^


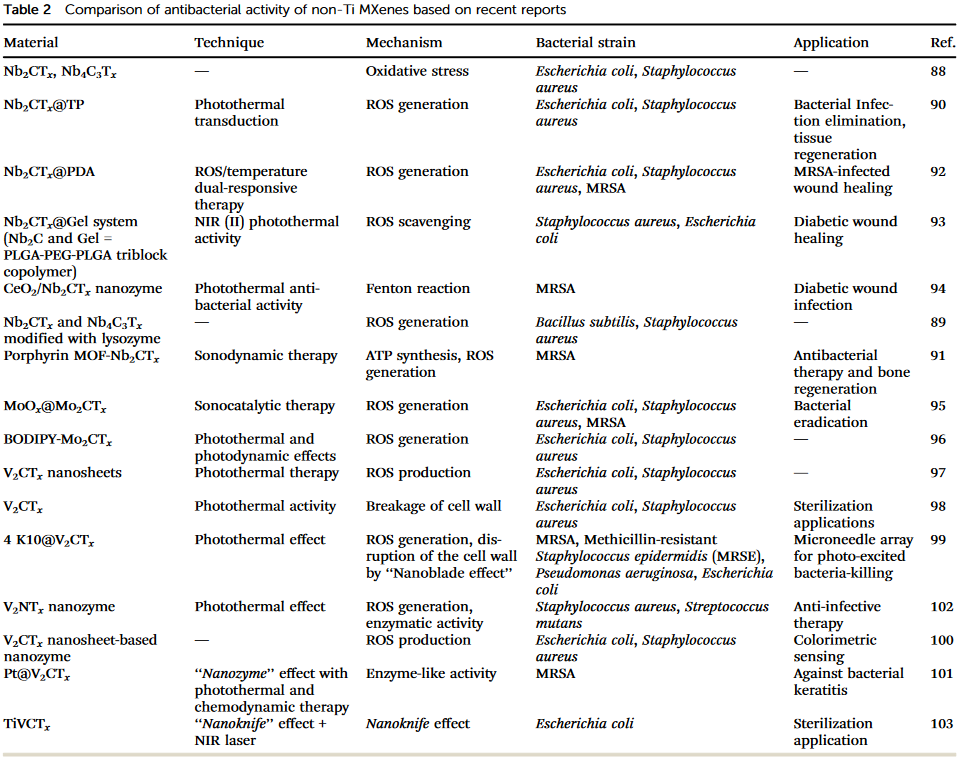


**Supplementary Figure S7:** The summary of recent research on non-MXene-based nanomaterials for antibacterial applications. This information is adapted from Table 2 in Gayathri et al. (2024) with permission from*, open access copyright license, Journal of Materials Chemistry B, RSC Publisher*.^https://doi.org/10.1039/D4TB01904K^


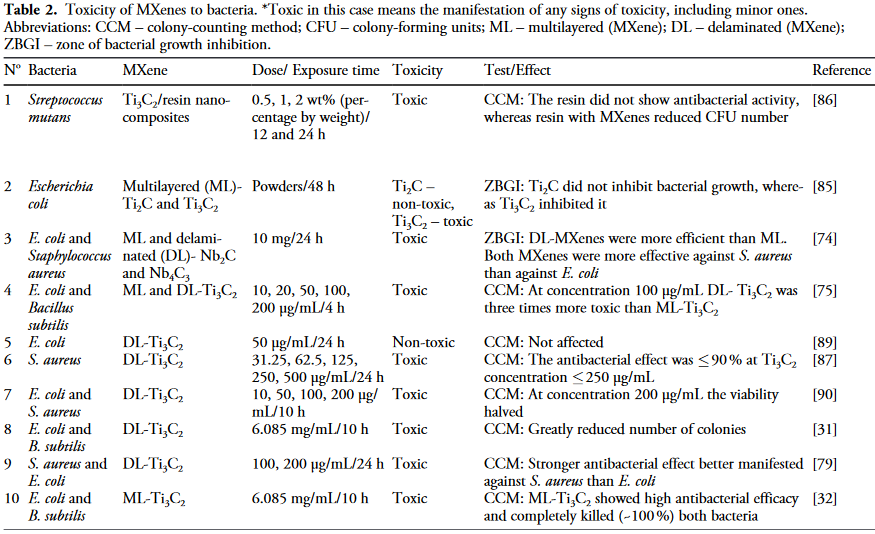


**Supplementary Figure S8:** The summary of recent research on non-MXene-based nanomaterials for treatment of antibacterial activities. This information is adapted from Table 2 in Dmytriv et al. (2024) with permission from*, The Chemical Records, Wiley Publisher*.^https://doi.org/10.1002/tcr.202300338^


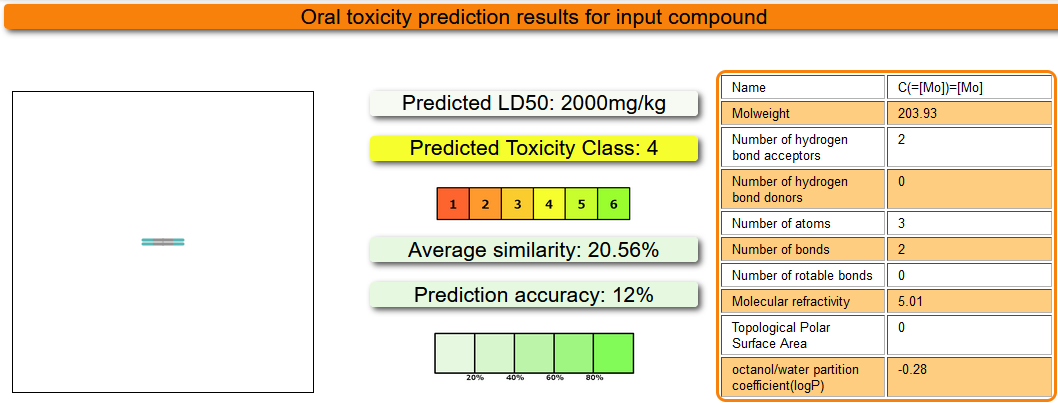


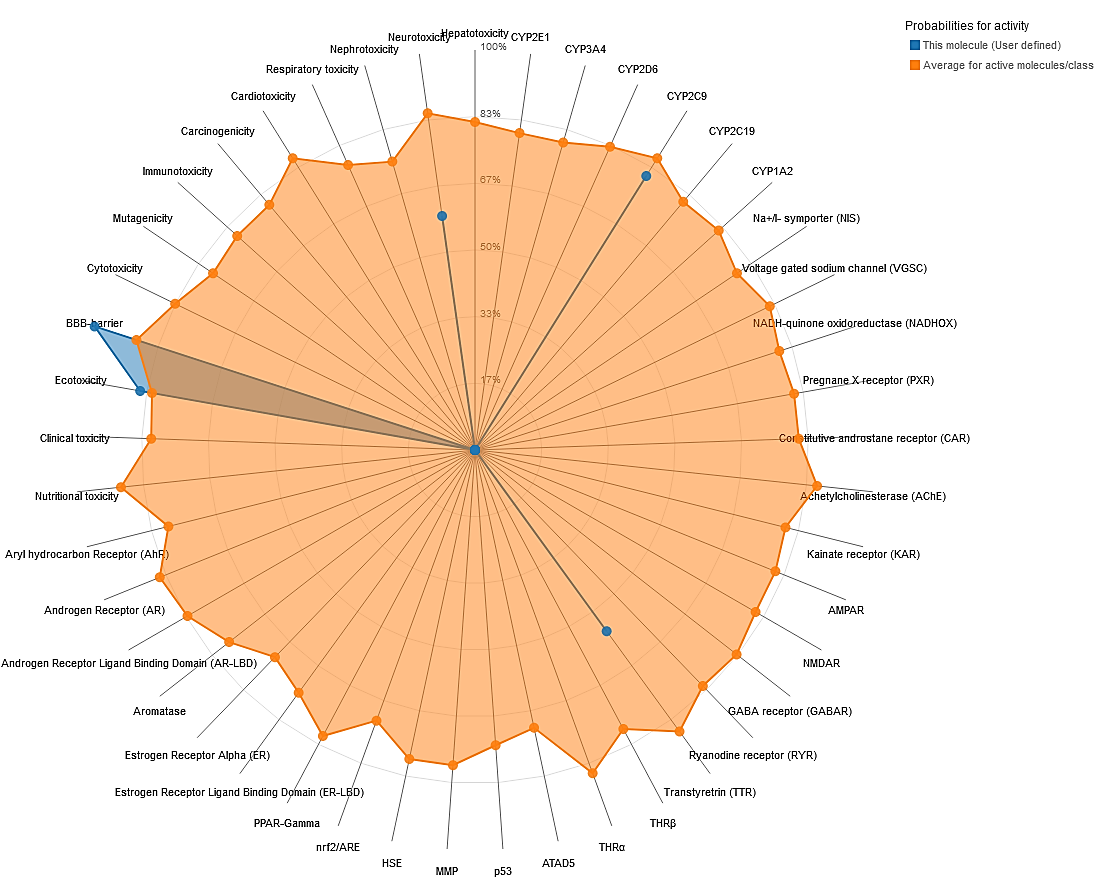


**Supplementary Figure S9:** The biocompatibility/toxicity evaluations of molybdenum carbide-based MXenes (Mo_2_CT_x_ MXene film, quantum dots, monolayer solution, organ-shaped multilayer, and nitrogen-doped quantum dots (Molecular formula: CMo_2_, MW: 203.9 g/mol, SMILES: C(=[Mo])=[Mo], PubChem: 10987348). <https://pubchem.ncbi.nlm.nih.gov/compound/10987348>. The toxicity radar chart is intended to illustrate the confidence of positive toxicity predictions with different bio-systems (quick results) of this chemical composition compared to the average of its class. Priyanka Banerjee, Emanuel Kemmler, Mathias Dunkel, Robert Preissner, ProTox 3.0: a webserver for the prediction of toxicity of chemicals, *Nucleic Acids Research*, Volume 52, Issue W1, 5 J 2024, Pages W513–W520 <https://tox.charite.de/>.<https://doi.org/10.1093/nar/gkae303>


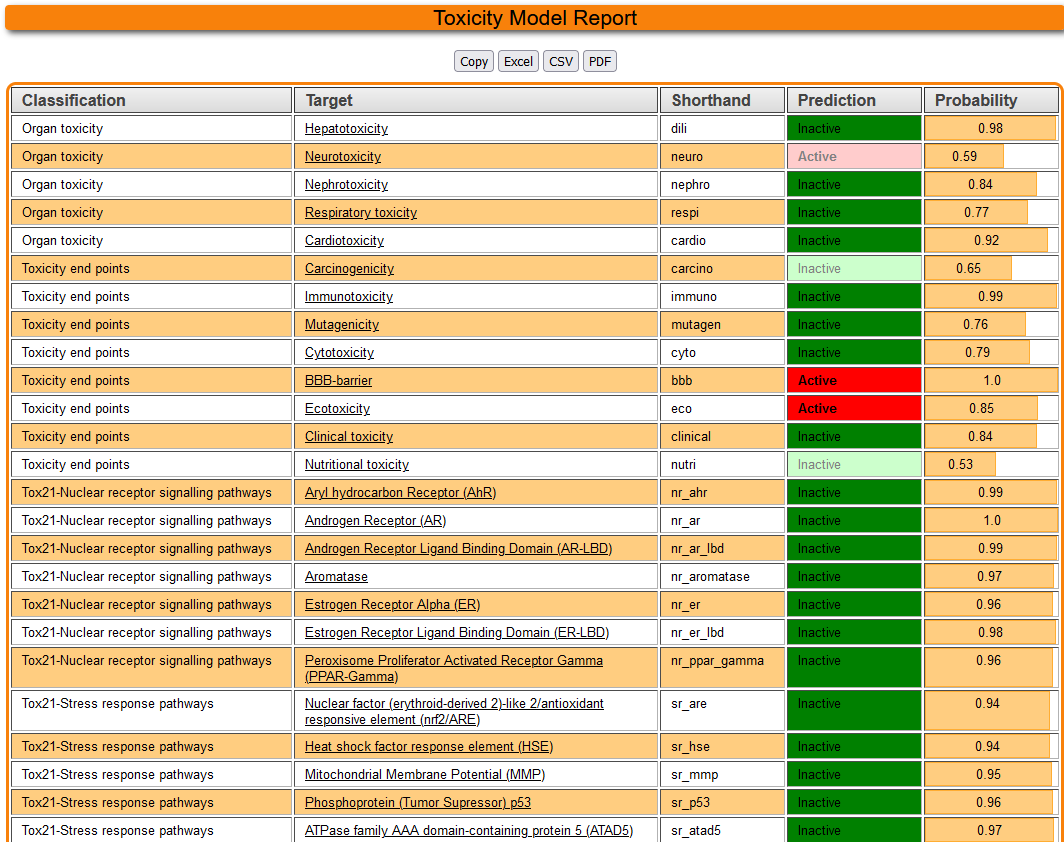


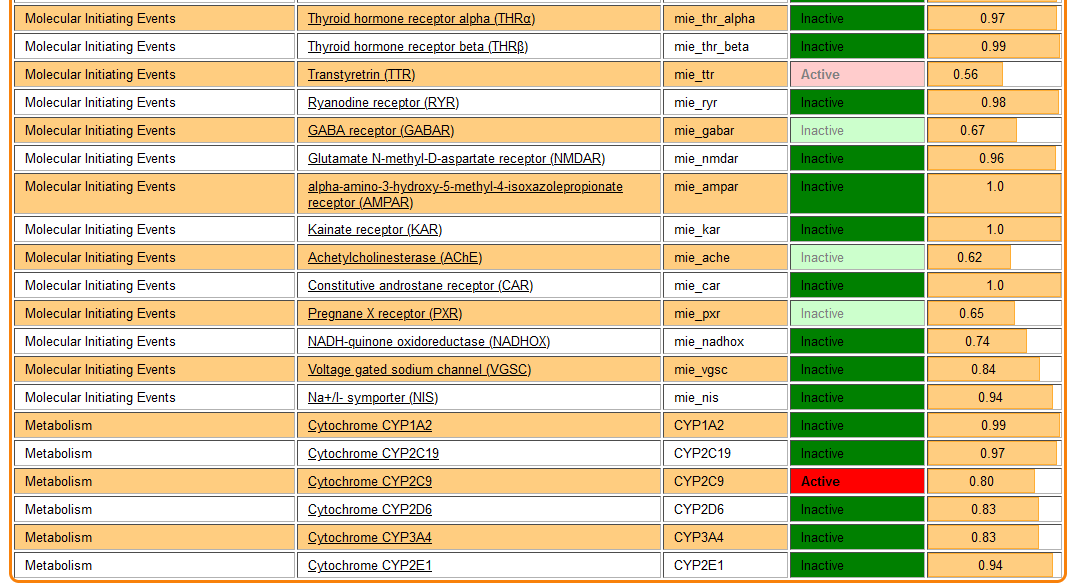


**Supplementary Figure S10:** The toxicity evaluations (screenshot) for this material to illustrate the confidence of biocompatibility/toxicity predictions with bio-systems. Priyanka Banerjee, Kemmler et al, ProTox 3.0: a webserver for the prediction of toxicity of chemicals, *Nucleic Acids Research*, Volume 52, I W1, 2024, P W513–W520 <https://tox.charite.de/>.<https://doi.org/10.1093/nar/gkae303>


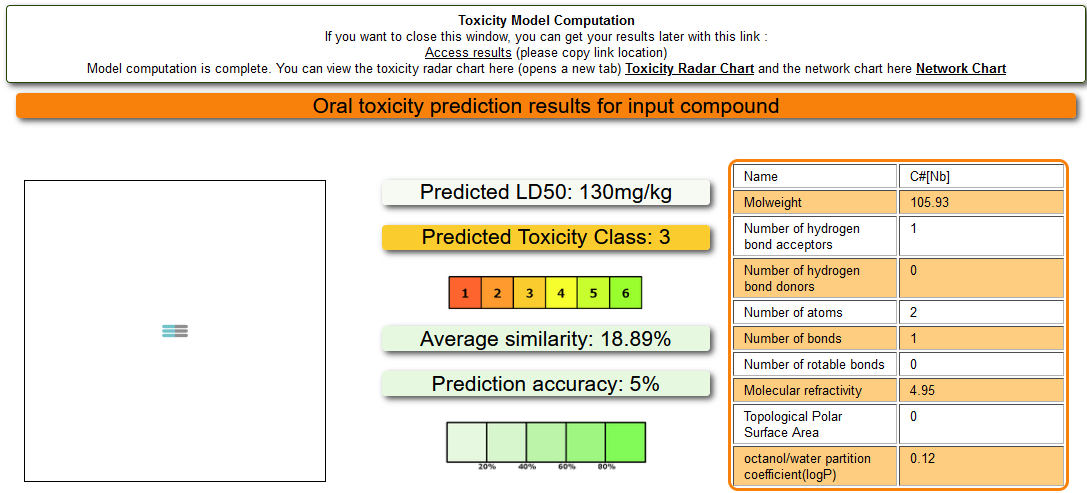


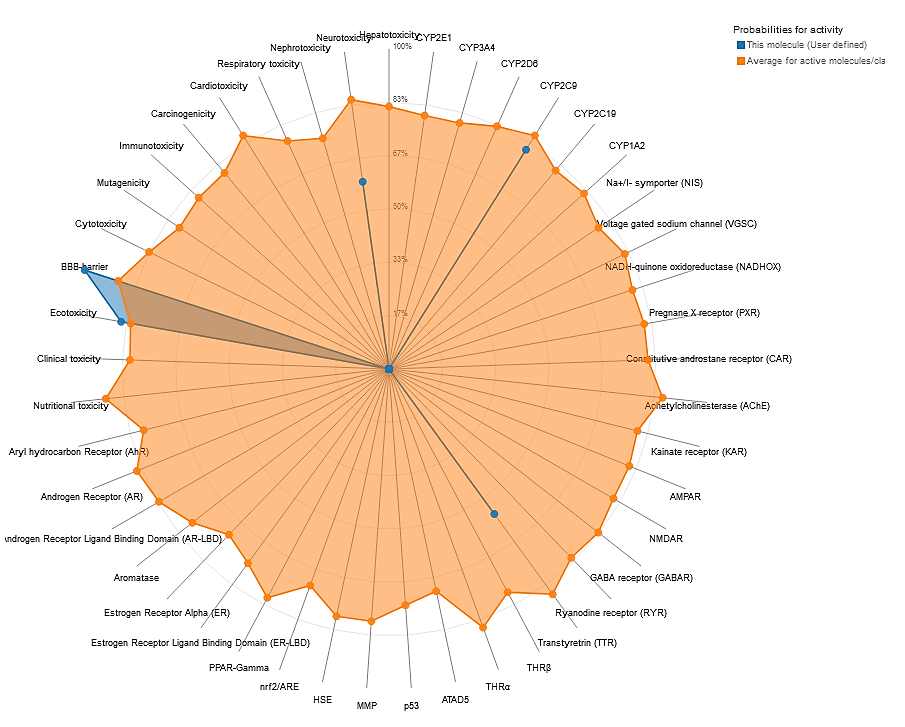


**Supplementary Figure S11:** The biocompatibility/toxicity evaluations of niobium carbide-based MXenes (Nb_2_CT_x_ MXene film, quantum dots, nanowire, powder, platinum-doped Nb_2_C, organ-shaped multilayered, nitriding Nb_2_C, porous foam, ITO/PET/silicon substrate Nb_2_C MXene, single/few layered powder, manganese intercalation Nb_2_C, monolayer Nb_2_C DMSO/Ethanol solution, functionalized monolayer solution, molten salt-etched fluorine-free Nb-MXenes, single layered Nb2C in-situ loaded with gold/silver nanoparticles (Molecular formula: CHNb/NbC, Molecular Weight: 105.925 g/mol, SMILES: C#[Nb], PubChem ID: 4060793) <https://pubchem.ncbi.nlm.nih.gov/compound/4060793>. Software by: Priyanka Banerjee, Emanuel Kemmler, Mathias Dunkel, Robert Preissner, ProTox 3.0: a webserver for the prediction of toxicity of chemicals, *Nucleic Acids Research*, Volume 52, Issue W1, 5 J 2024, Pages W513–W520 <https://tox.charite.de/>.<https://doi.org/10.1093/nar/gkae303>


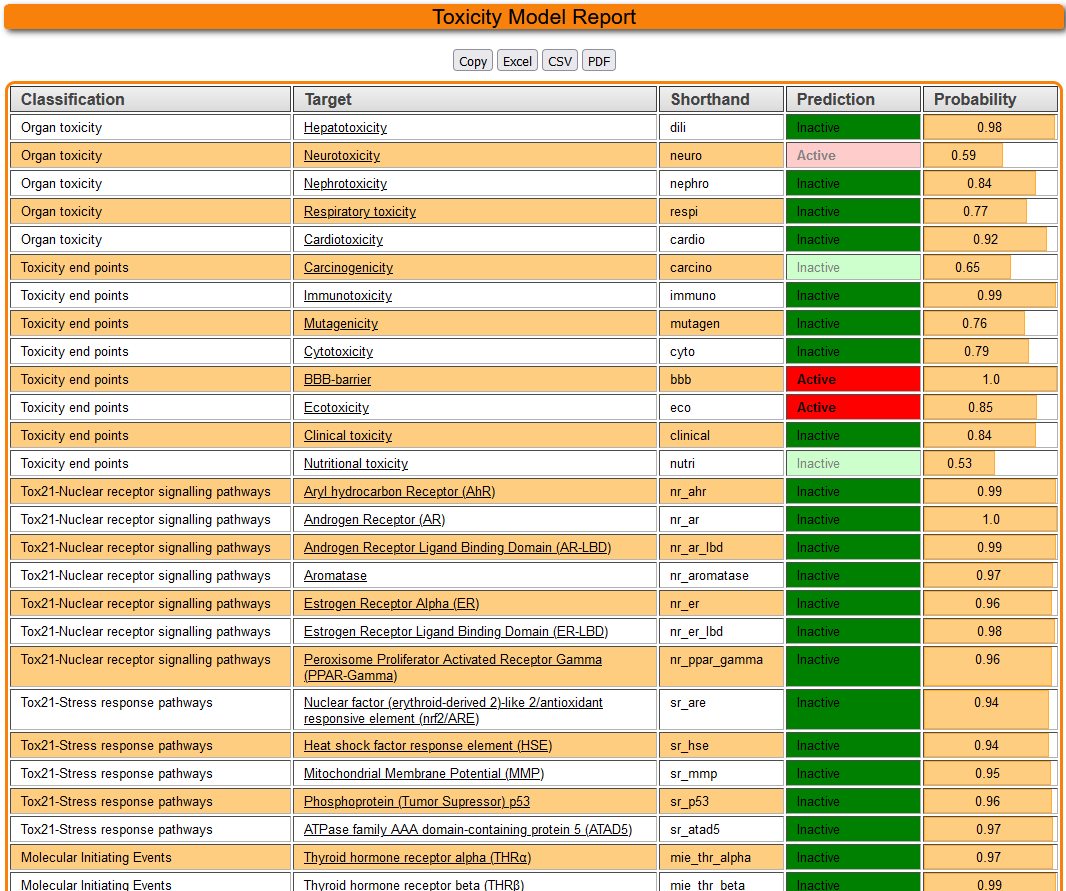


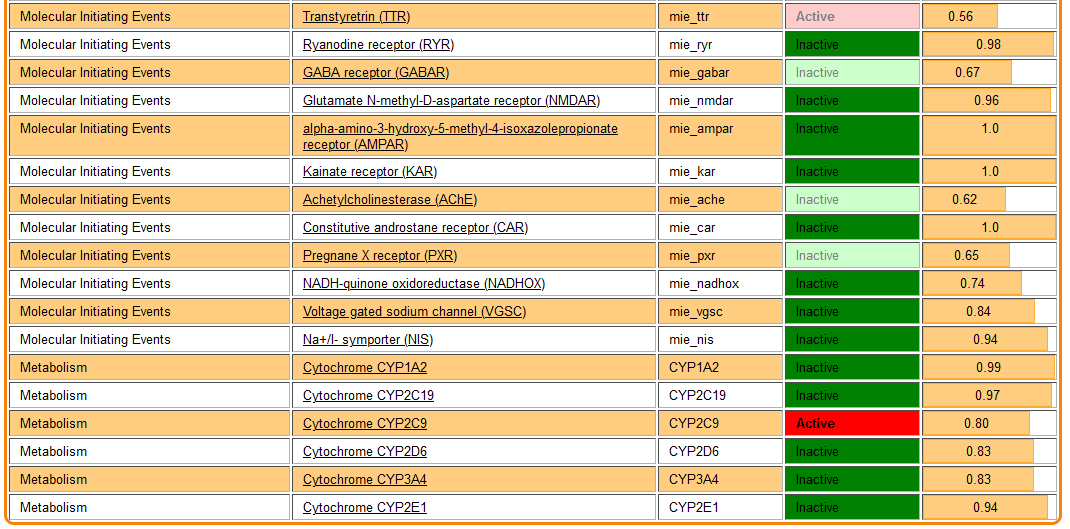


**Supplementary Figure S12:** The toxicity evaluations (screenshot) for this material to illustrate the confidence of biocompatibility/toxicity predictions with bio-systems. Priyanka Banerjee, Kemmler et al, ProTox 3.0: a webserver for the prediction of toxicity of chemicals, *Nucleic Acids Research*, Volume 52, I W1, 2024, P W513–W520 <https://tox.charite.de/>.<https://doi.org/10.1093/nar/gkae303>


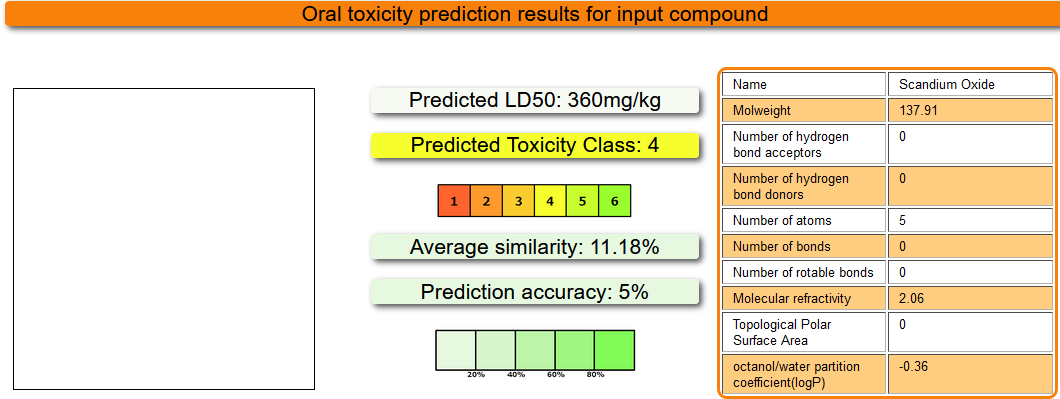


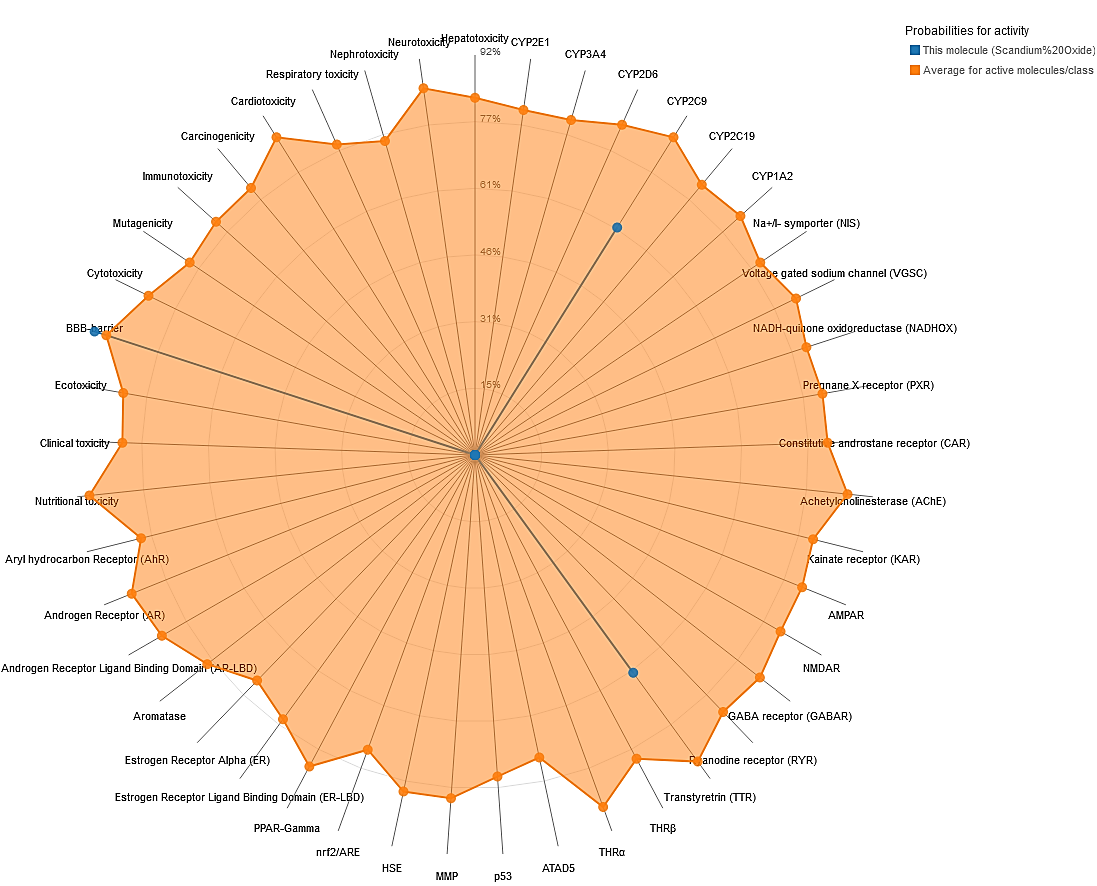


**Supplementary Figure S13:** The predicted biocompatibility/toxicity evaluation of scandium oxide (screenshot). Software by: Priyanka Banerjee, Emanuel Kemmler, Mathias Dunkel, Robert Preissner, ProTox 3.0: a webserver for the prediction of toxicity of chemicals, *Nucleic Acids Research*, Vol 52, I. W1, 5 July 2024, W513–W520 <https://tox.charite.de/>.<https://doi.org/10.1093/nar/gkae303>


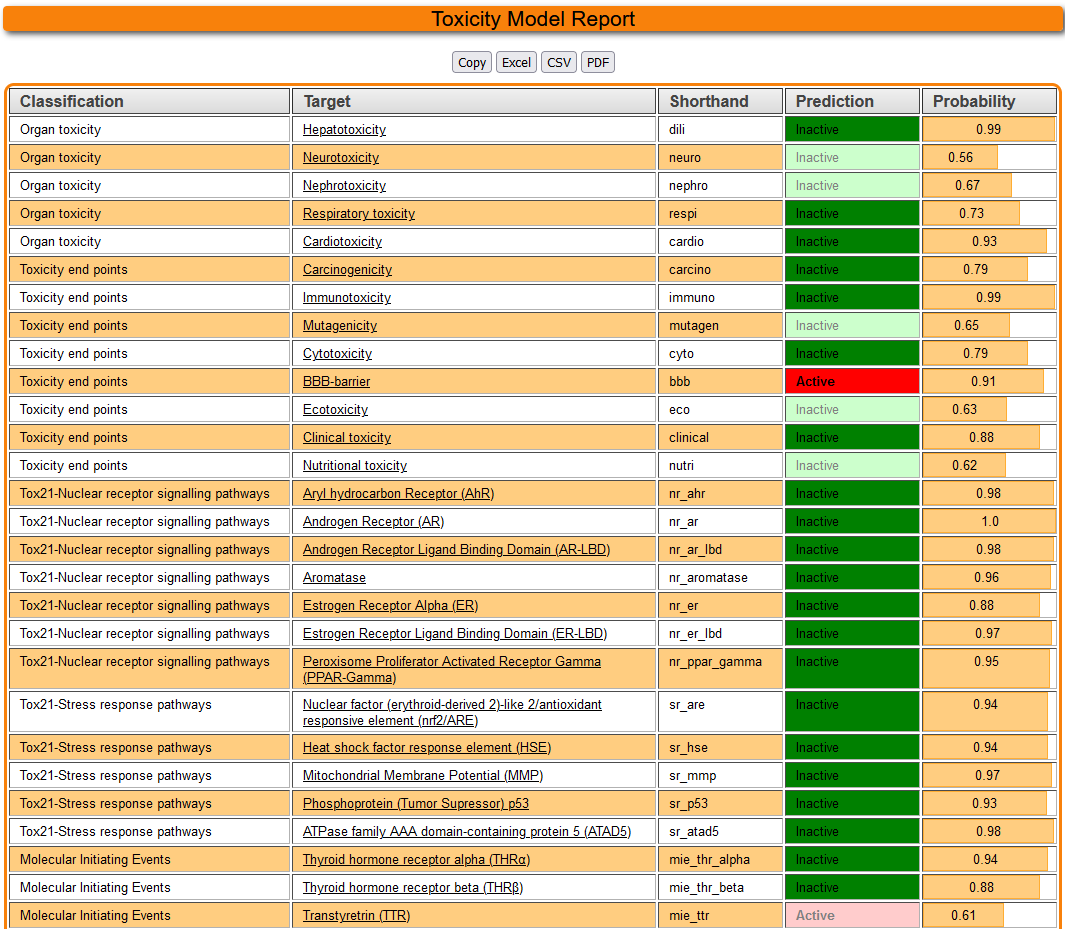


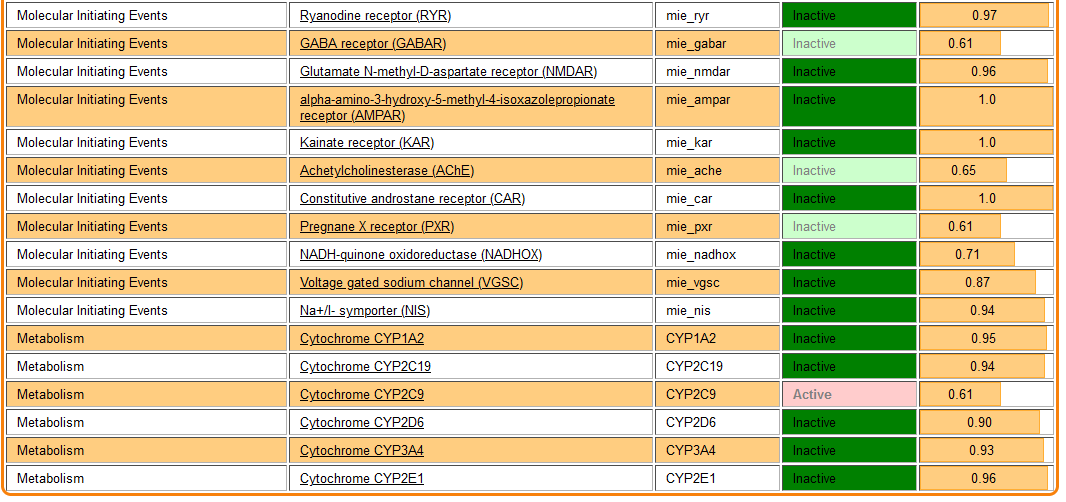


**Supplementary Figure S14:** The toxicity evaluations (screenshot) for scandium oxide to illustrate the confidence of biocompatibility/toxicity predictions with bio-systems. Priyanka Banerjee, Kemmler et al, ProTox 3.0: a webserver for the prediction of toxicity of chemicals, *Nucleic Acids Research*, Volume 52, I W1, 2024, P W513–W520 <https://tox.charite.de/>.<https://doi.org/10.1093/nar/gkae303>


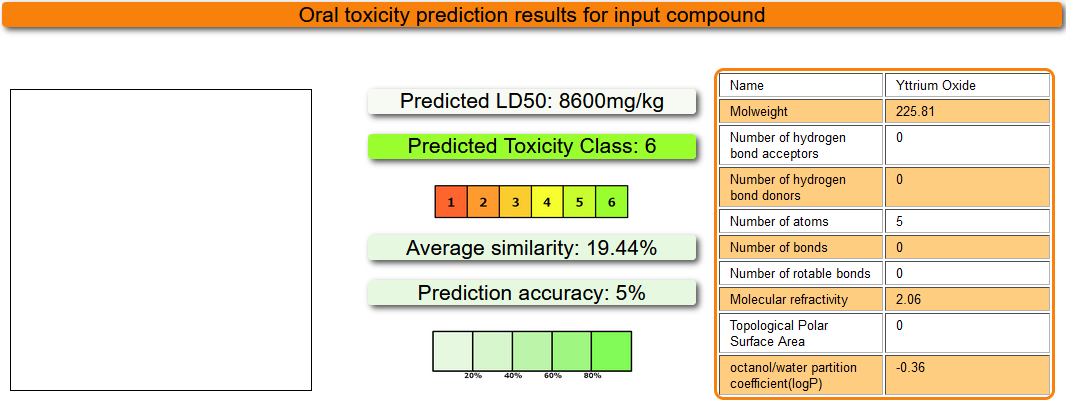


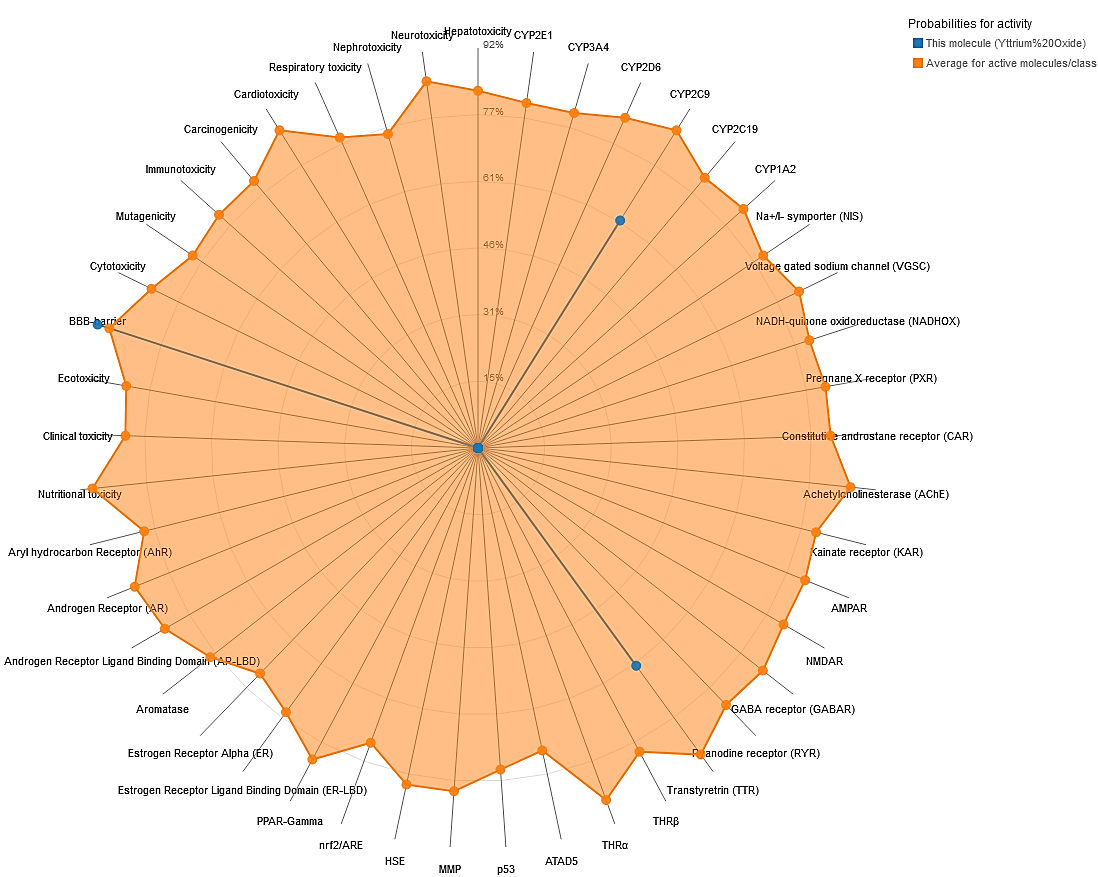


**Supplementary Figure S15:** The predicted biocompatibility/toxicity evaluation of yttrium oxide (screenshot). Software by: Priyanka Banerjee, Emanuel Kemmler, Mathias Dunkel, Robert Preissner, ProTox 3.0: a webserver for the prediction of toxicity of chemicals, *Nucleic Acids Research*, Vol 52, I. W1, 5 July 2024, W513–W520 <https://tox.charite.de/>.<https://doi.org/10.1093/nar/gkae303>


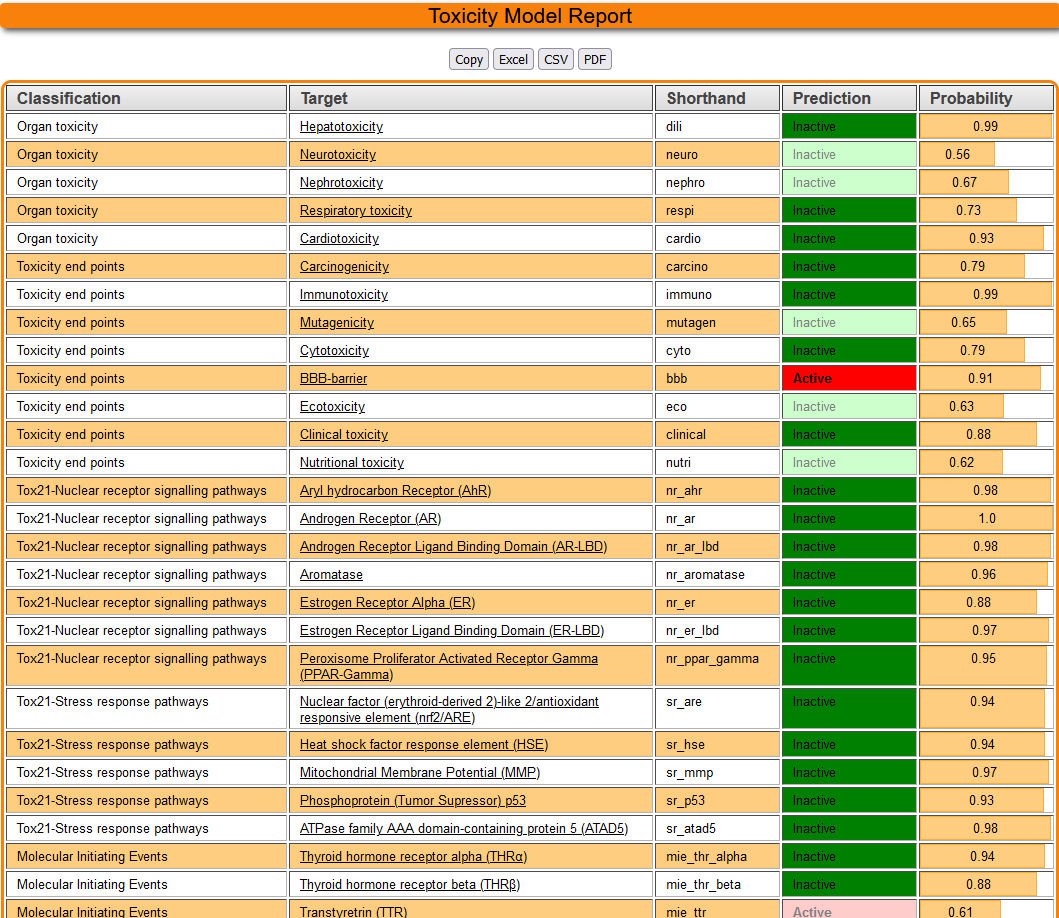


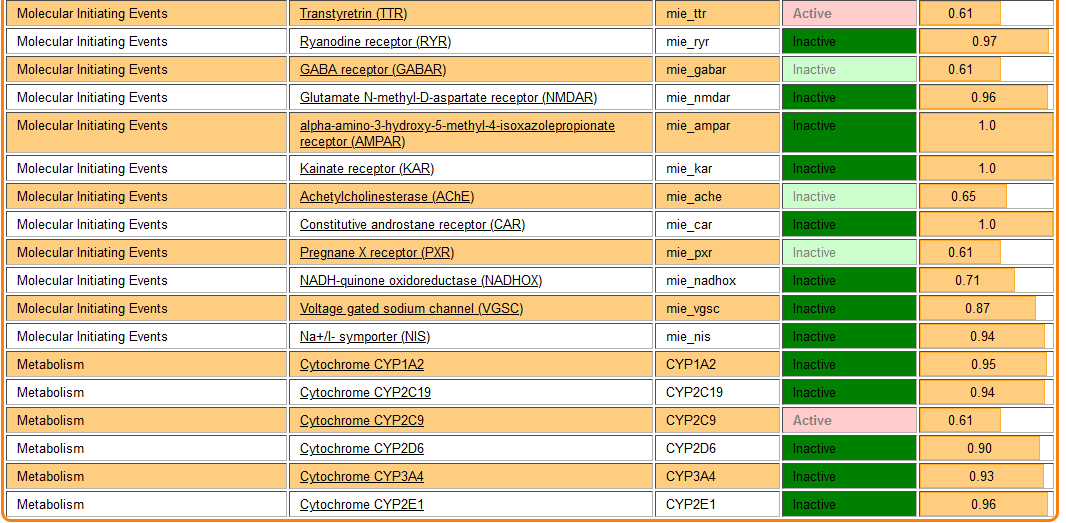


**Supplementary Figure S16:** The toxicity evaluations (screenshot) for yttrium oxide to illustrate the confidence of biocompatibility/toxicity predictions with bio-systems. Priyanka Banerjee, Kemmler et al, ProTox 3.0: a webserver for the prediction of toxicity of chemicals, *Nucleic Acids Research*, Volume 52, I W1, 2024, P W513–W520 <https://tox.charite.de/>.<https://doi.org/10.1093/nar/gkae303>


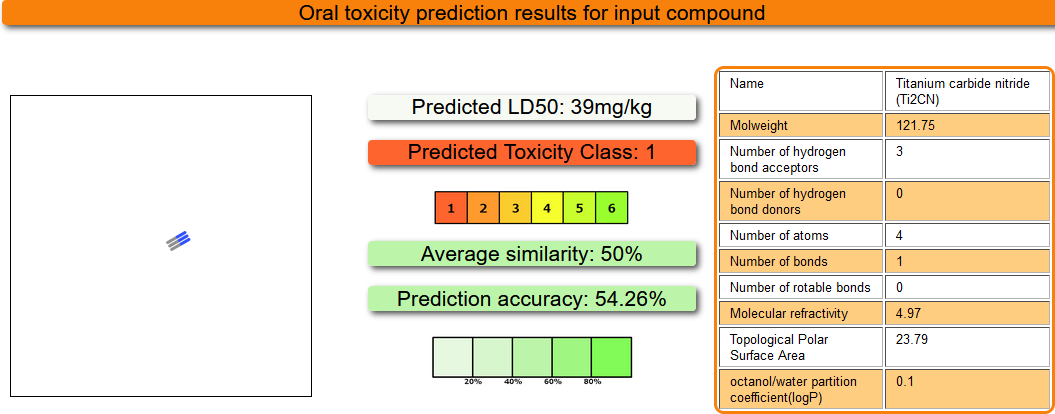


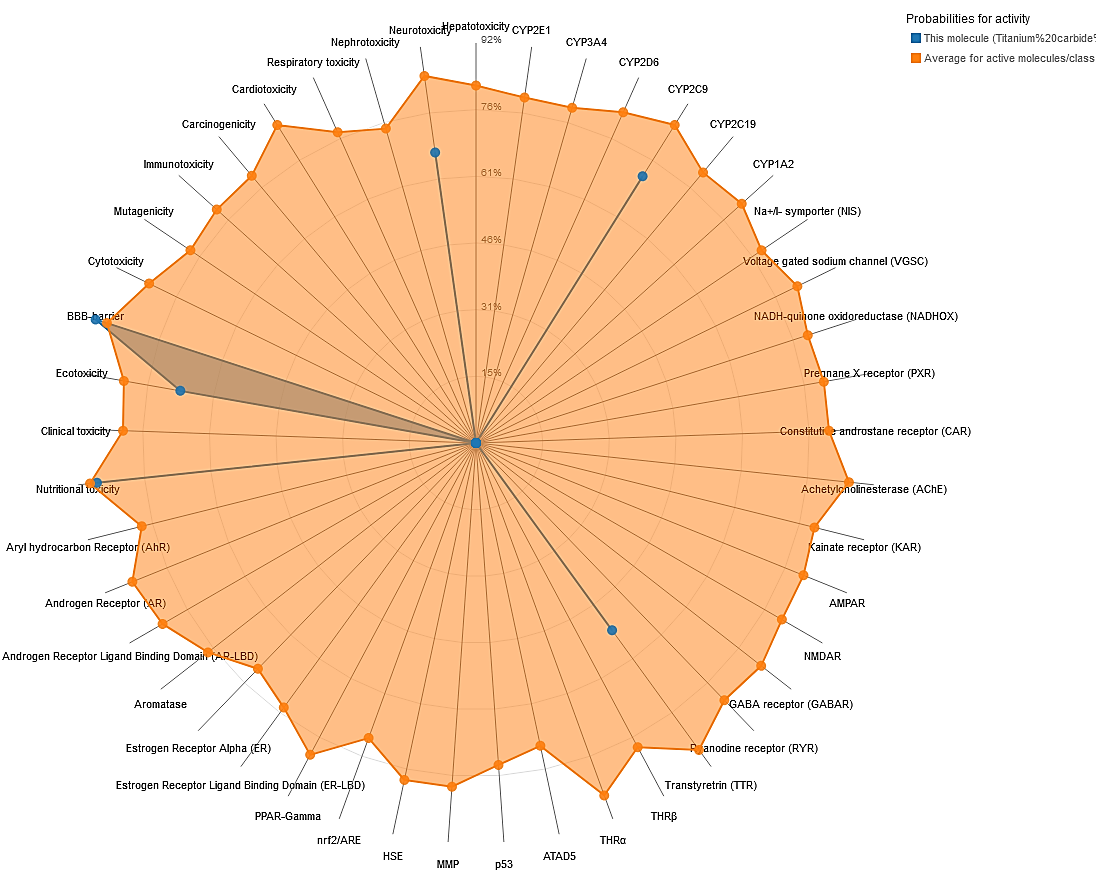


**Supplementary Figure S17:** The predicted biocompatibility/toxicity evaluation of titanium carbide nitride (Ti_2_CN). Software by: Priyanka Banerjee, Emanuel Kemmler, Mathias Dunkel, Robert Preissner, ProTox 3.0: a webserver for the prediction of toxicity of chemicals, *Nucleic Acids Research*, Vol 52, I. W1, 5 July 2024, W513–W520 <https://tox.charite.de/>.<https://doi.org/10.1093/nar/gkae303>


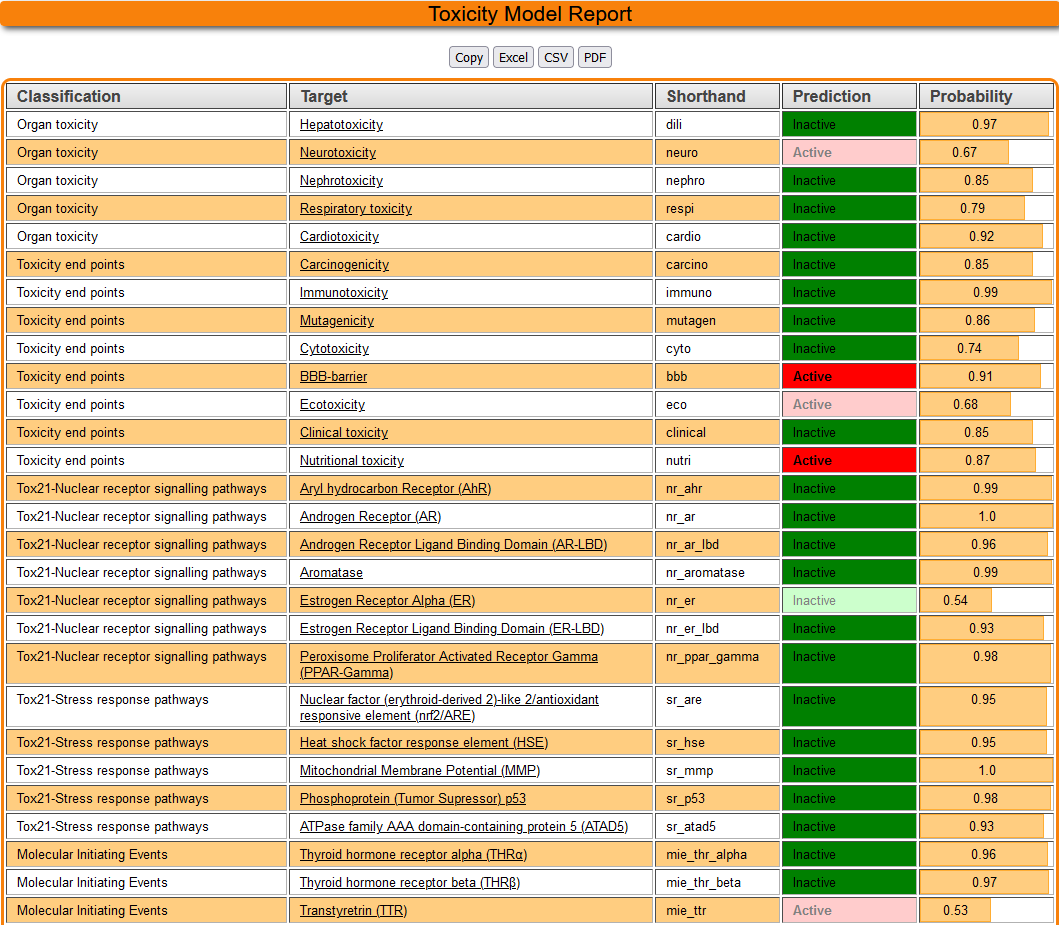


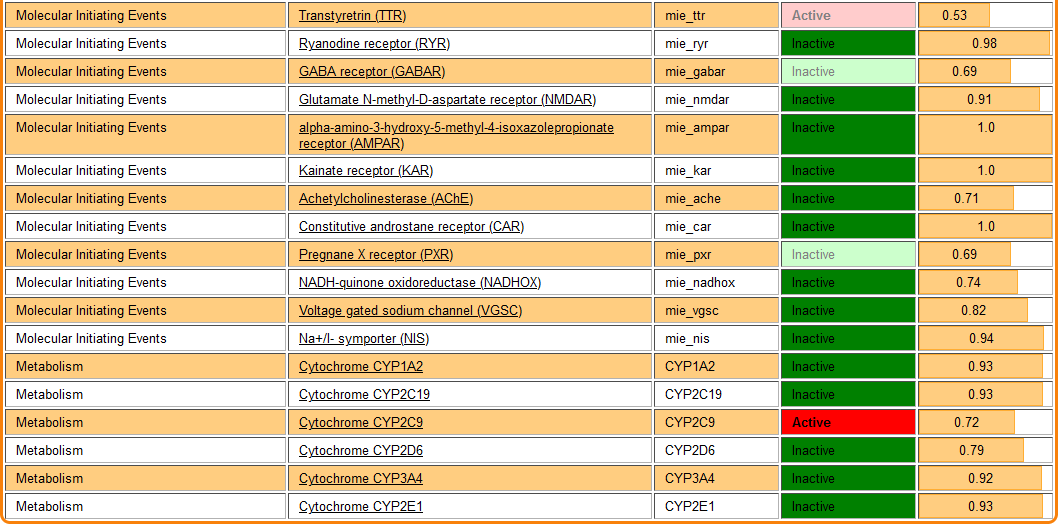


**Supplementary Figure S18:** The toxicity evaluations for titanium carbide nitride (Ti_2_CN) to illustrate the confidence of biocompatibility/toxicity predictions with bio-systems. Priyanka Banerjee, Kemmler et al, ProTox 3.0: a webserver for the prediction of toxicity of chemicals, *Nucleic Acids Research*, 52, 2024, W513–W520 <https://tox.charite.de/>.<https://doi.org/10.1093/nar/gkae303>


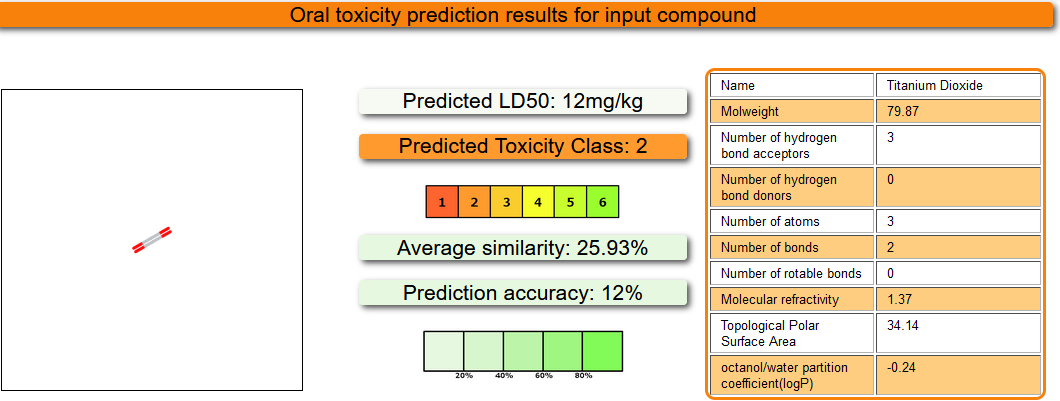


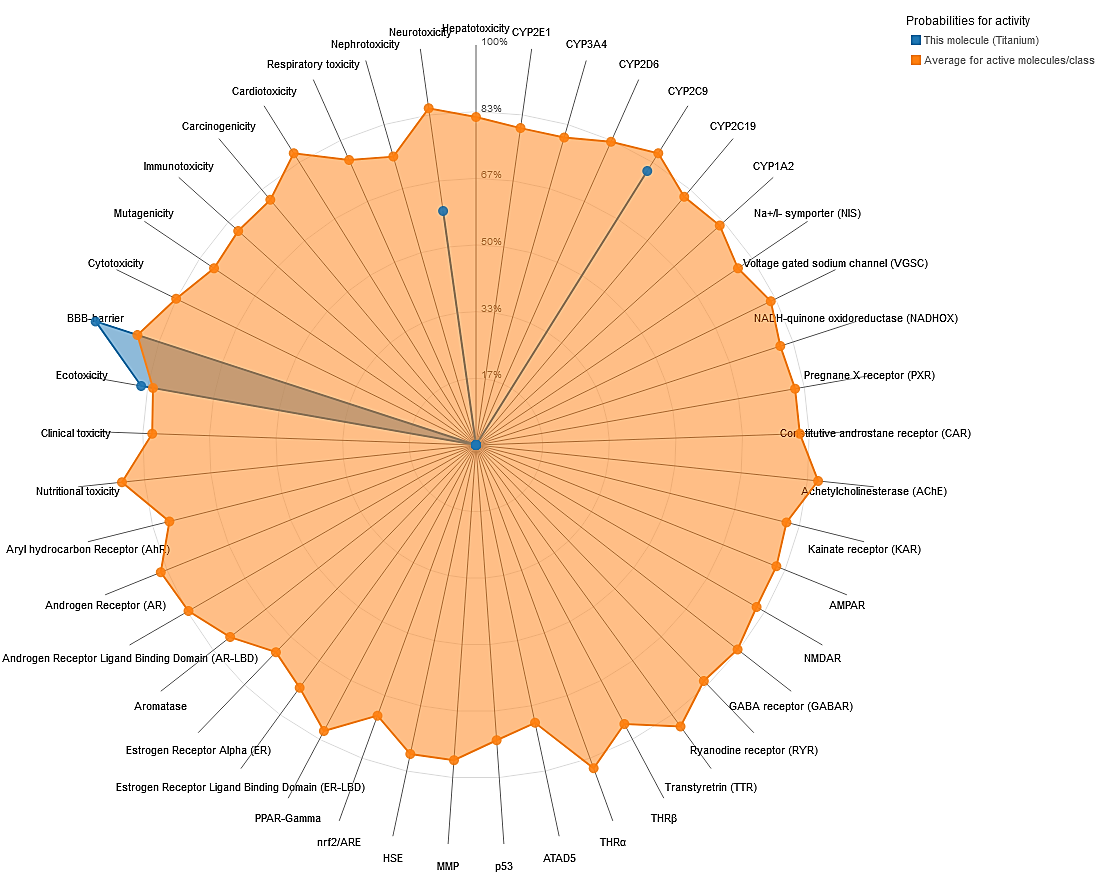


**Supplementary Figure S19:** The predicted biocompatibility/toxicity evaluation of titanium dioxide (screenshot). Software by: Priyanka Banerjee, Emanuel Kemmler, Mathias Dunkel, Robert Preissner, ProTox 3.0: a webserver for the prediction of toxicity of chemicals, *Nucleic Acids Research*, Vol 52, I. W1, 5 July 2024, W513–W520 <https://tox.charite.de/>.<https://doi.org/10.1093/nar/gkae303>


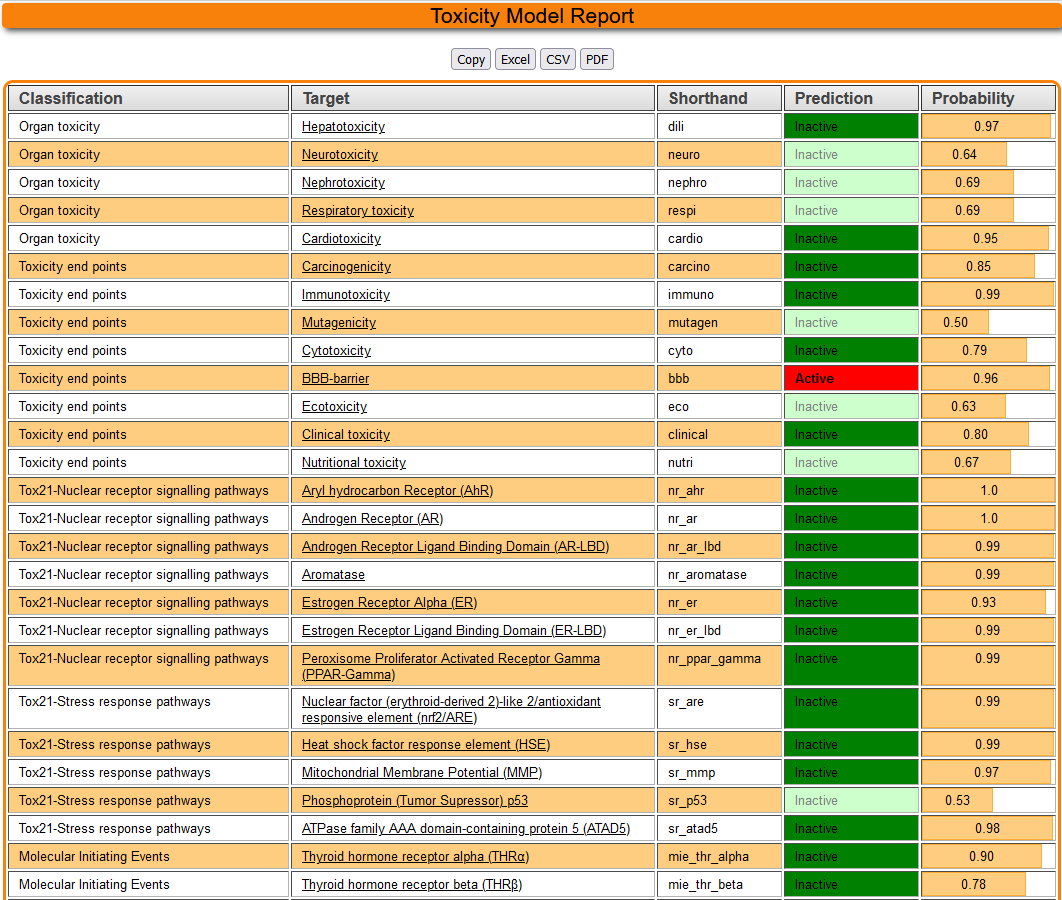


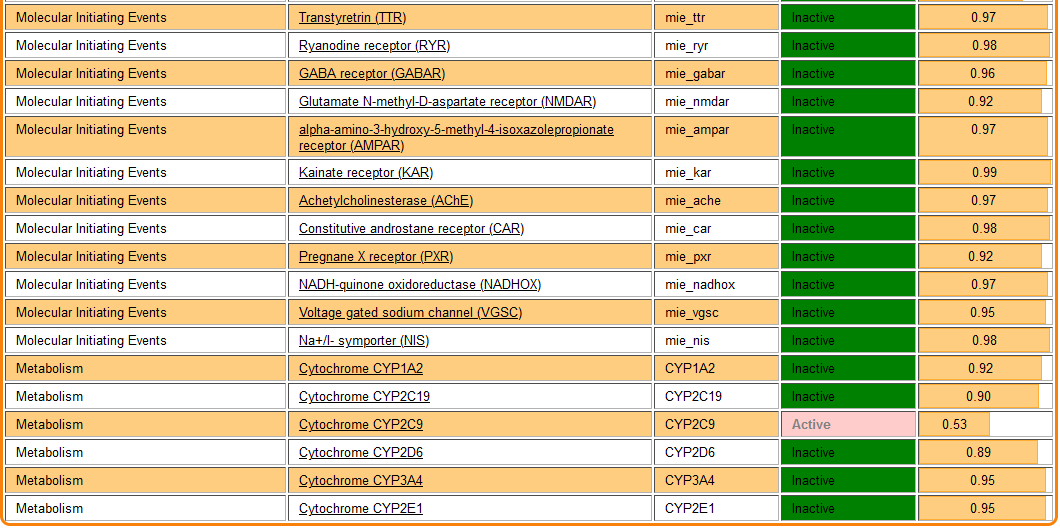


**Supplementary Figure S20:** The biocompatibility/toxicity evaluations for titanium dioxide to illustrate the confidence of biocompatibility/toxicity predictions with bio-systems. Priyanka Banerjee, Kemmler et al, ProTox 3.0: a webserver for the prediction of toxicity of chemicals, *Nucleic Acids Research*, 52, 2024, W513–W520 <https://tox.charite.de/>.<https://doi.org/10.1093/nar/gkae303>


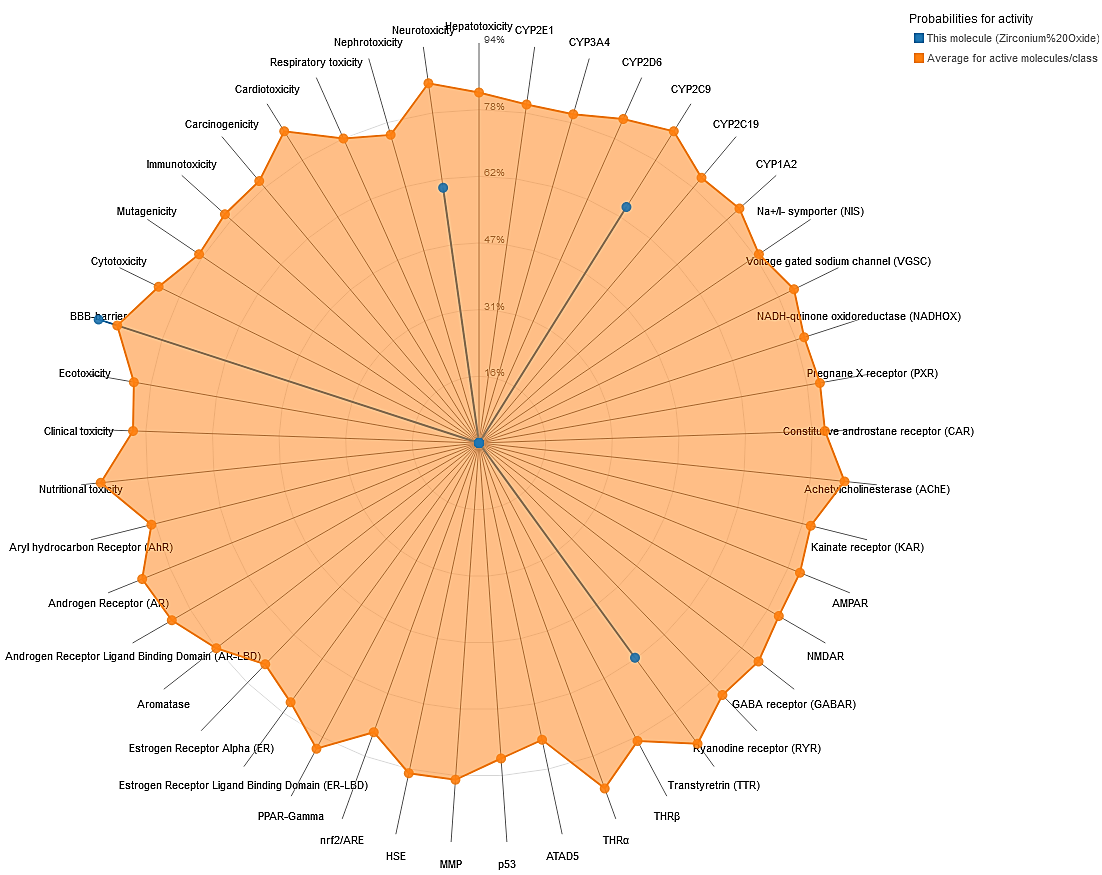


**Supplementary Figure S21:** The predicted biocompatibility/toxicity evaluation of zirconium /zirconium oxide (screenshot). Software by: Priyanka Banerjee, Emanuel Kemmler, Mathias Dunkel, Robert Preissner, ProTox 3.0: a webserver for the prediction of toxicity of chemicals, *Nucleic Acids Research*, Volume 52, Issue W1, 5 July 2024, pages W513–W520 <https://tox.charite.de/>.<https://doi.org/10.1093/nar/gkae303>


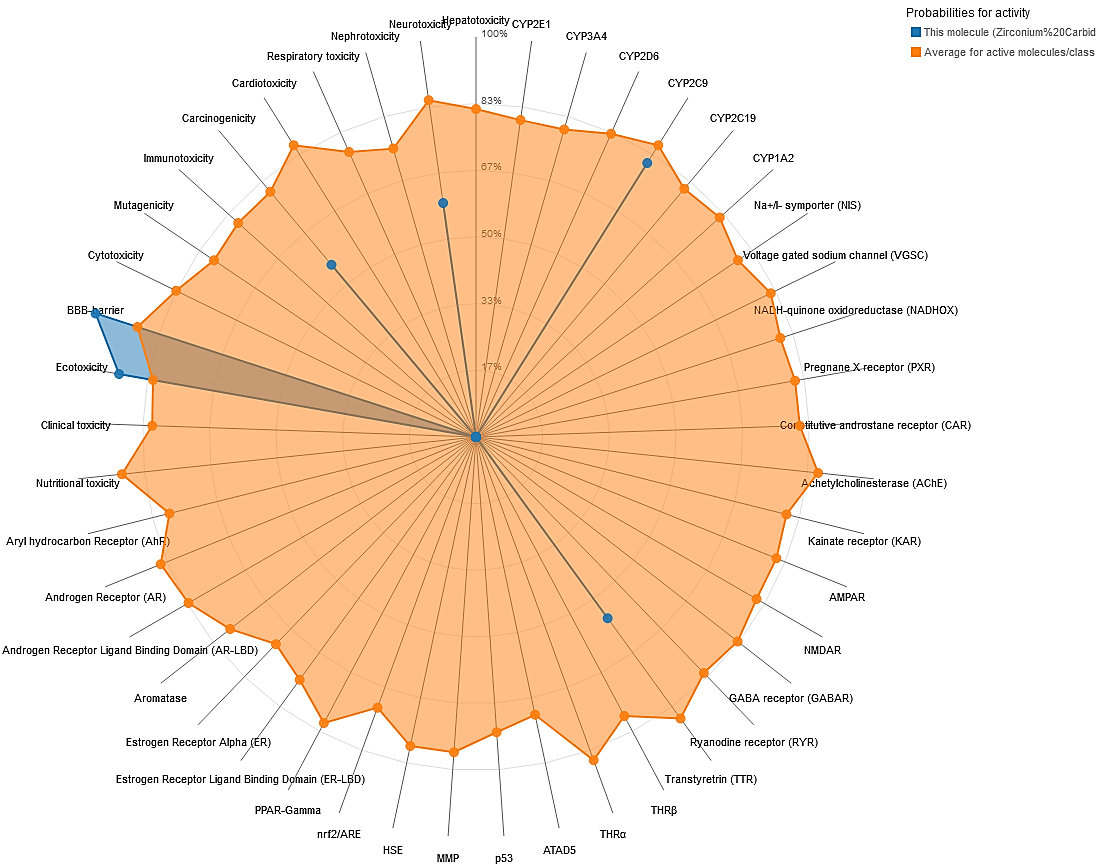


**Supplementary Figure S22:** The predicted biocompatibility/toxicity evaluation of zirconium/ zirconium carbide (screenshot). Software by: Priyanka Banerjee, Emanuel Kemmler, Mathias Dunkel, Robert Preissner, ProTox 3.0: a webserver for the prediction of toxicity of chemicals, *Nucleic Acids Research*, Volume 52, Issue W1, 5 July 2024, pages W513–W520 <https://tox.charite.de/>.<https://doi.org/10.1093/nar/gkae303>


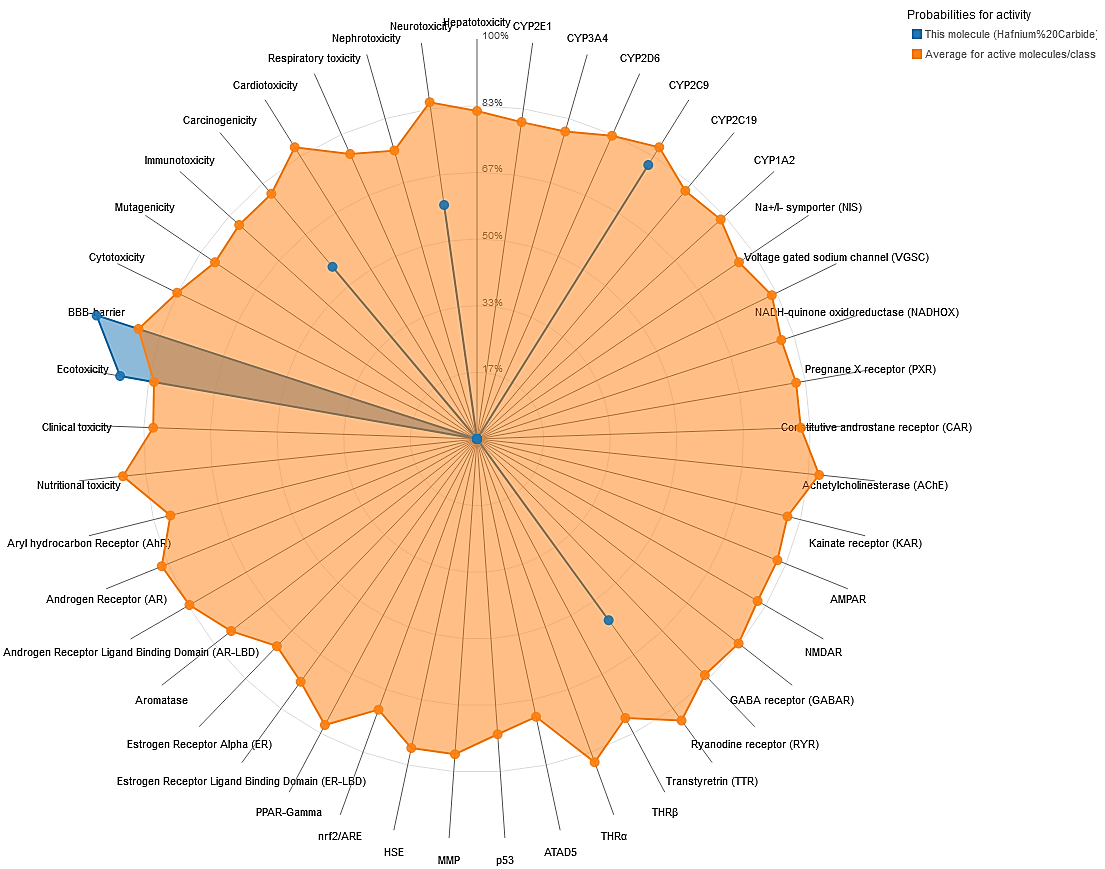


**Supplementary Figure S23:** The predicted biocompatibility/toxicity evaluation of hafnium carbide (screenshot). Software by: Priyanka Banerjee, Emanuel Kemmler, Mathias Dunkel, Robert Preissner, ProTox 3.0: a webserver for the prediction of toxicity of chemicals, *Nucleic Acids Research*, Volume 52, I W1, 5 J 2024, W513–W520 <https://tox.charite.de/>.<https://doi.org/10.1093/nar/gkae303>


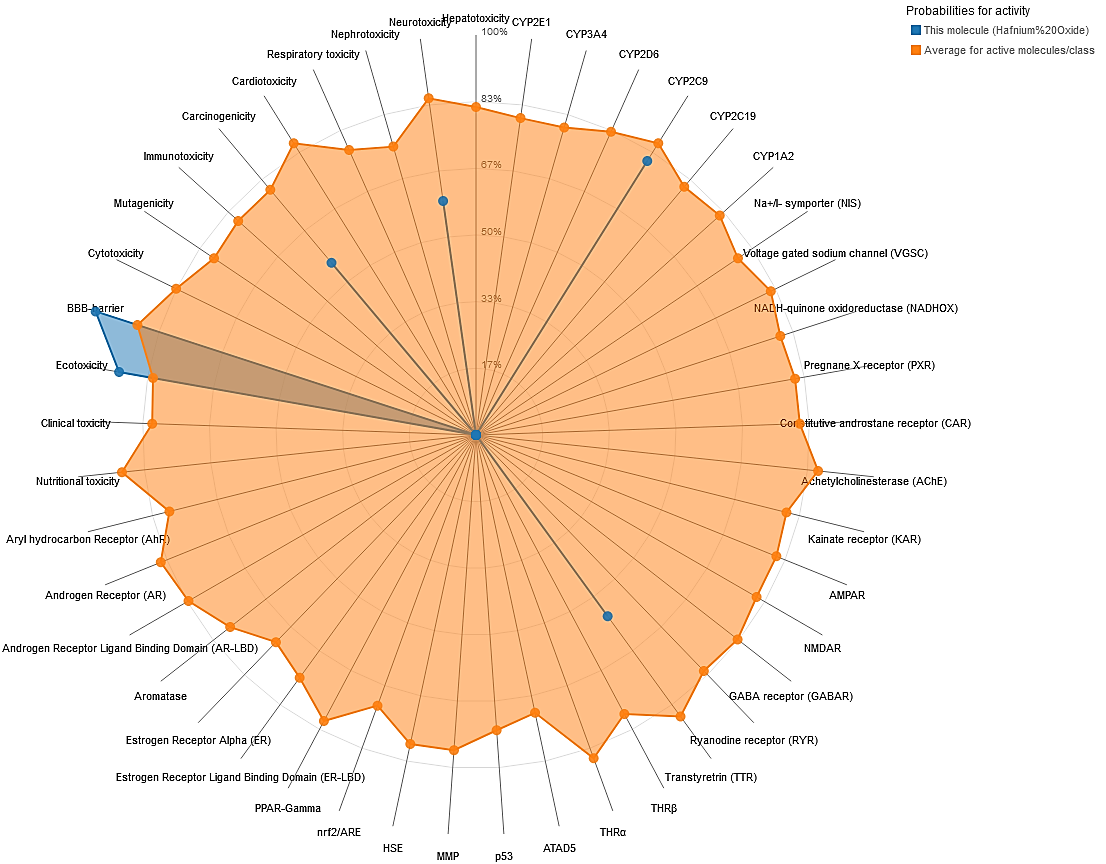


**Supplementary Figure S24:** The predicted biocompatibility/toxicity evaluation of hafnium oxide (screenshot). Software by: Priyanka Banerjee, Emanuel Kemmler, Mathias Dunkel, Robert Preissner, ProTox 3.0: a webserver for the prediction of toxicity of chemicals, *Nucleic Acids Research*, Volume 52, I W1, 5 J 2024, W513–W520 <https://tox.charite.de/>.<https://doi.org/10.1093/nar/gkae303>


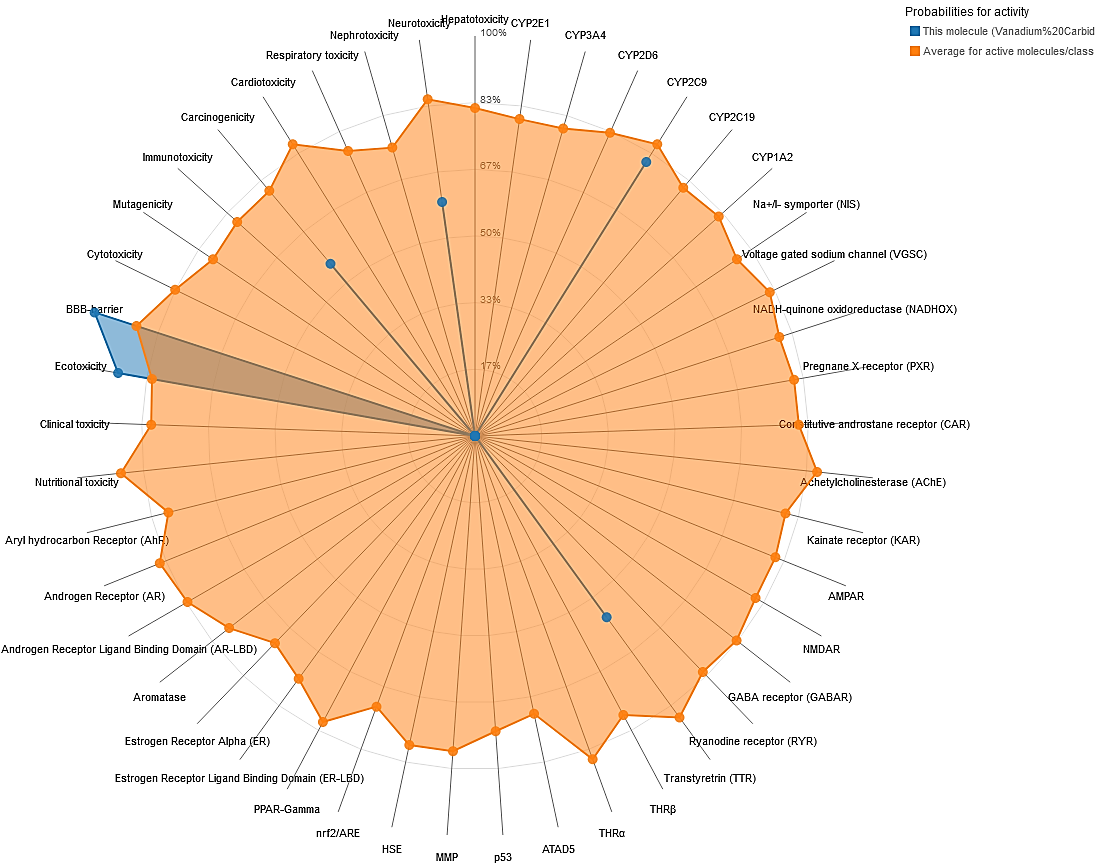


**Supplementary Figure S25:** The predicted biocompatibility/toxicity evaluation of vanadium carbide (screenshot). Software by: Priyanka Banerjee, Emanuel Kemmler, Mathias Dunkel, Robert Preissner, ProTox 3.0: a webserver for the prediction of toxicity of chemicals, *Nucleic Acids Research*, Volume 52, I W1, 5 J 2024, W513–W520 <https://tox.charite.de/>.<https://doi.org/10.1093/nar/gkae303>


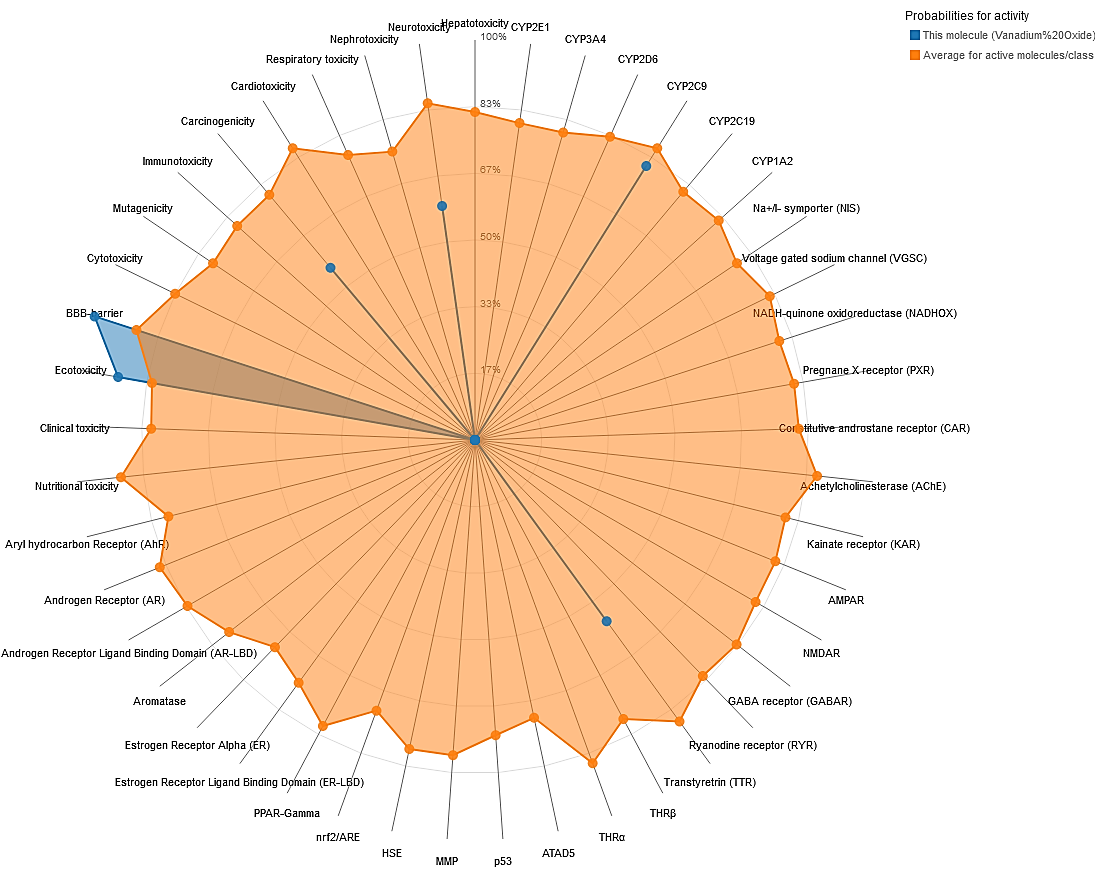


**Supplementary Figure S26:** The predicted biocompatibility/toxicity evaluation of vanadium oxide (screenshot). Software by: Priyanka Banerjee, Emanuel Kemmler, Mathias Dunkel, Robert Preissner, ProTox 3.0: a webserver for the prediction of toxicity of chemicals, *Nucleic Acids Research*, Volume 52, I W1, 5 J 2024, W513–W520 <https://tox.charite.de/>.<https://doi.org/10.1093/nar/gkae303>


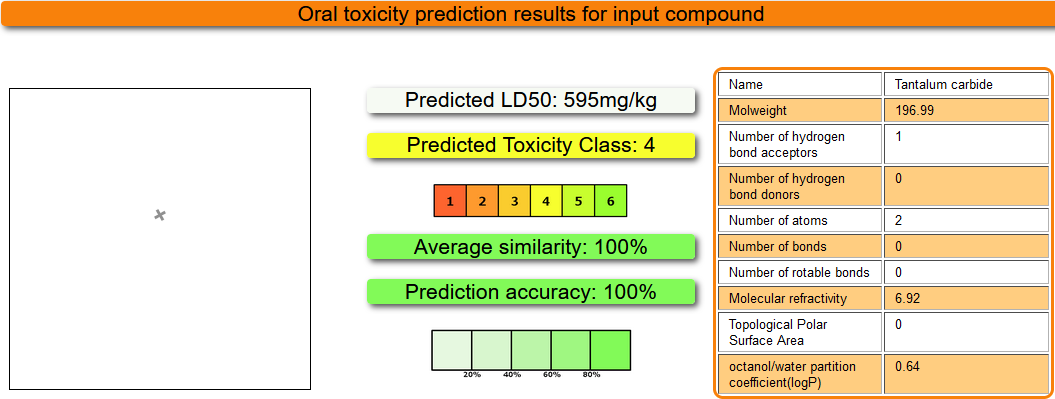


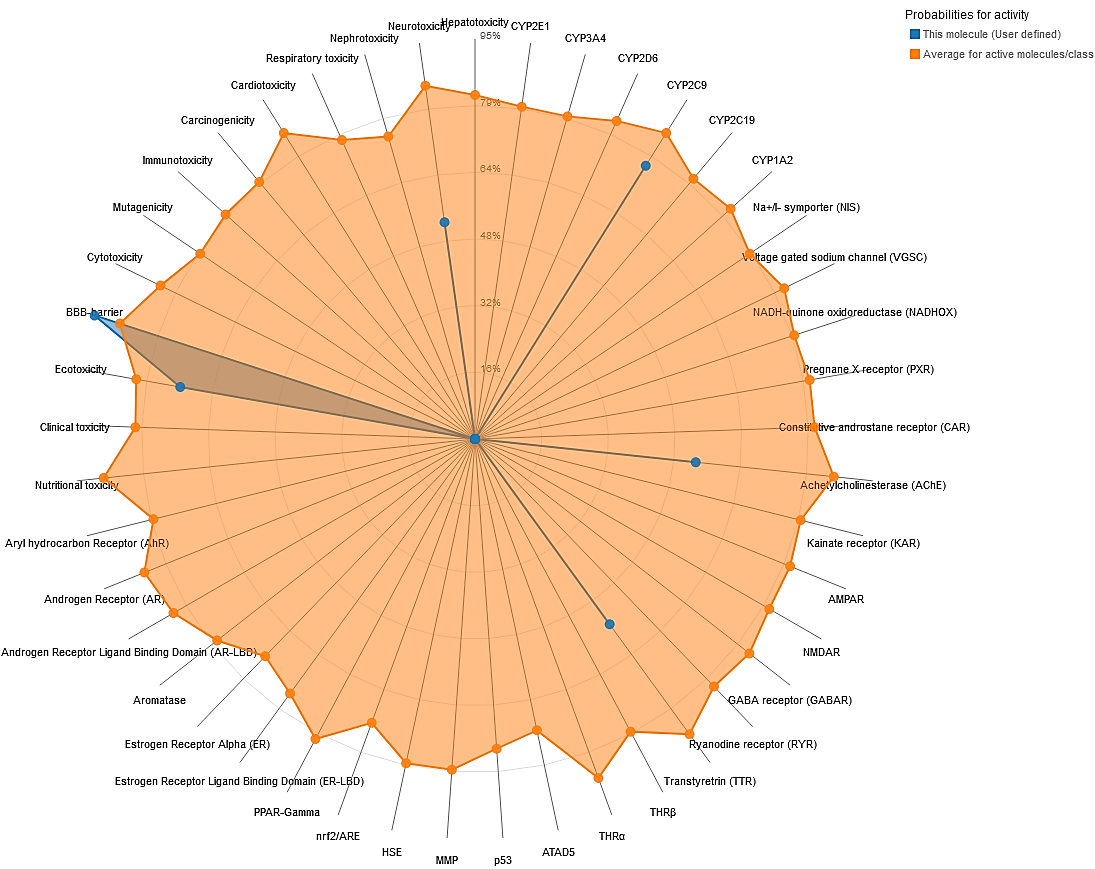


**Supplementary Figure S27:** The predicted biocompatibility/toxicity evaluation of tantalum carbide (screenshot). Software by: Priyanka Banerjee, Emanuel Kemmler, Mathias Dunkel, Robert Preissner, ProTox 3.0: a webserver for the prediction of toxicity of chemicals, *Nucleic Acids Research*, Vol 52, I. W1, 5 July 2024, W513–W520 <https://tox.charite.de/>.<https://doi.org/10.1093/nar/gkae303>


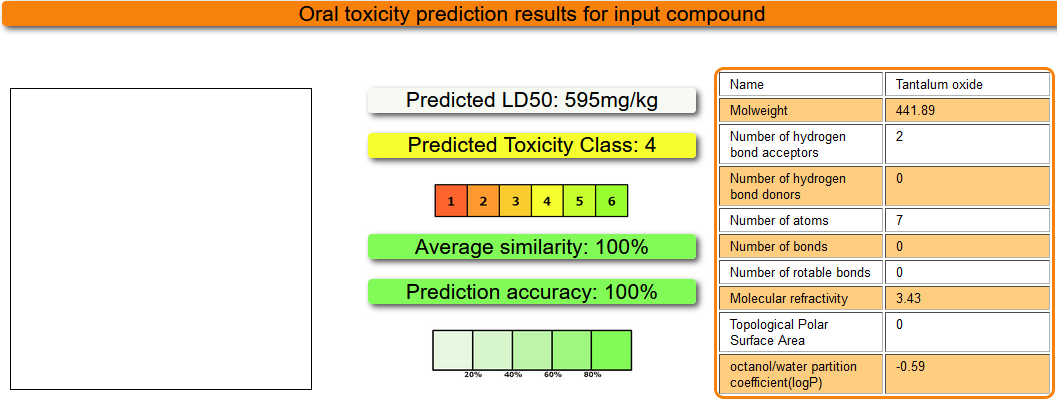


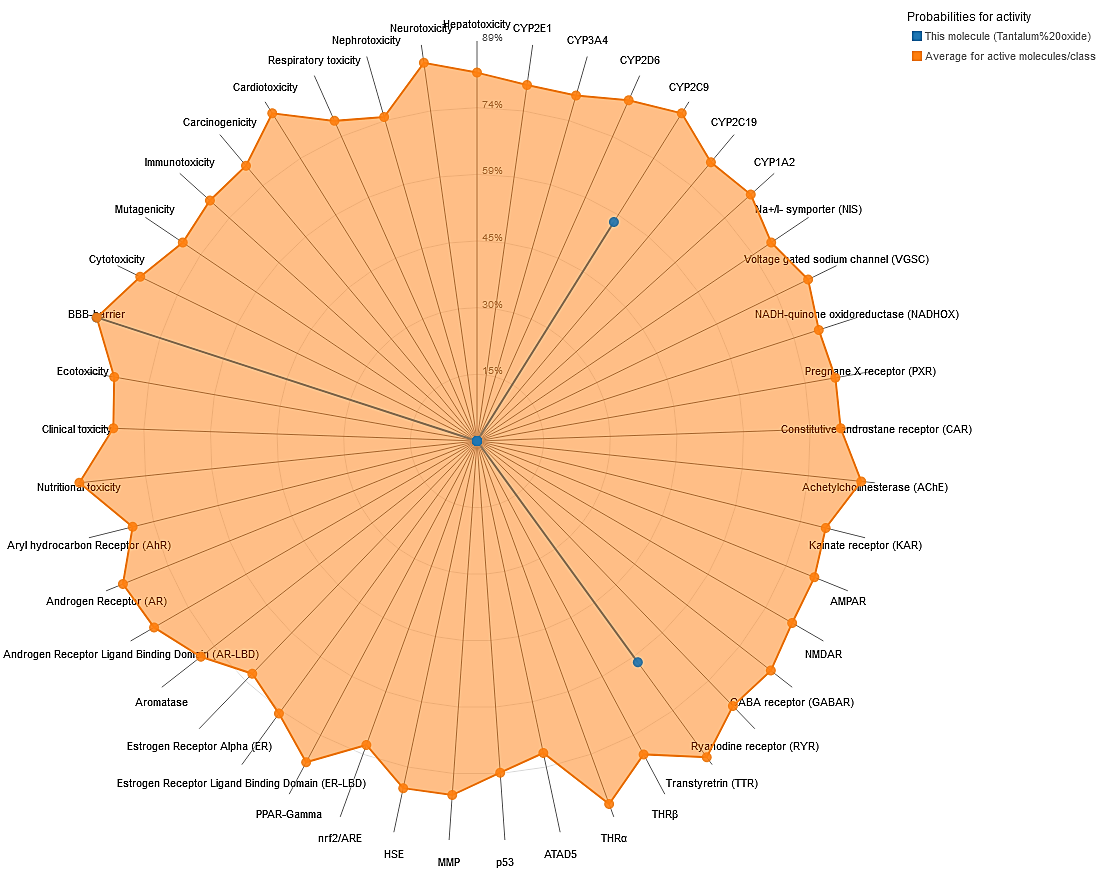


**Supplementary Figure S28:** The predicted biocompatibility/toxicity evaluation of tantalum oxide (screenshot). Software by: Priyanka Banerjee, Emanuel Kemmler, Mathias Dunkel, Robert Preissner, ProTox 3.0: a webserver for the prediction of toxicity of chemicals, *Nucleic Acids Research*, Volume 52, I W1, 5 J 2024, W513–W520 <https://tox.charite.de/>.<https://doi.org/10.1093/nar/gkae303>


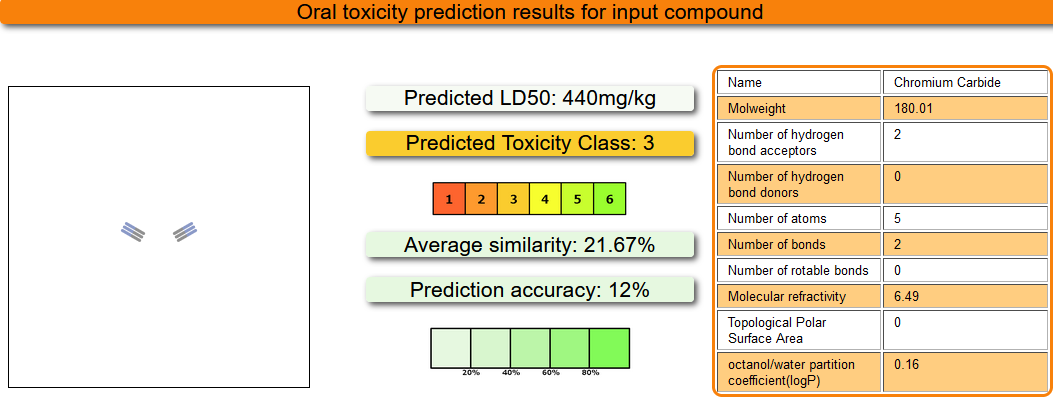


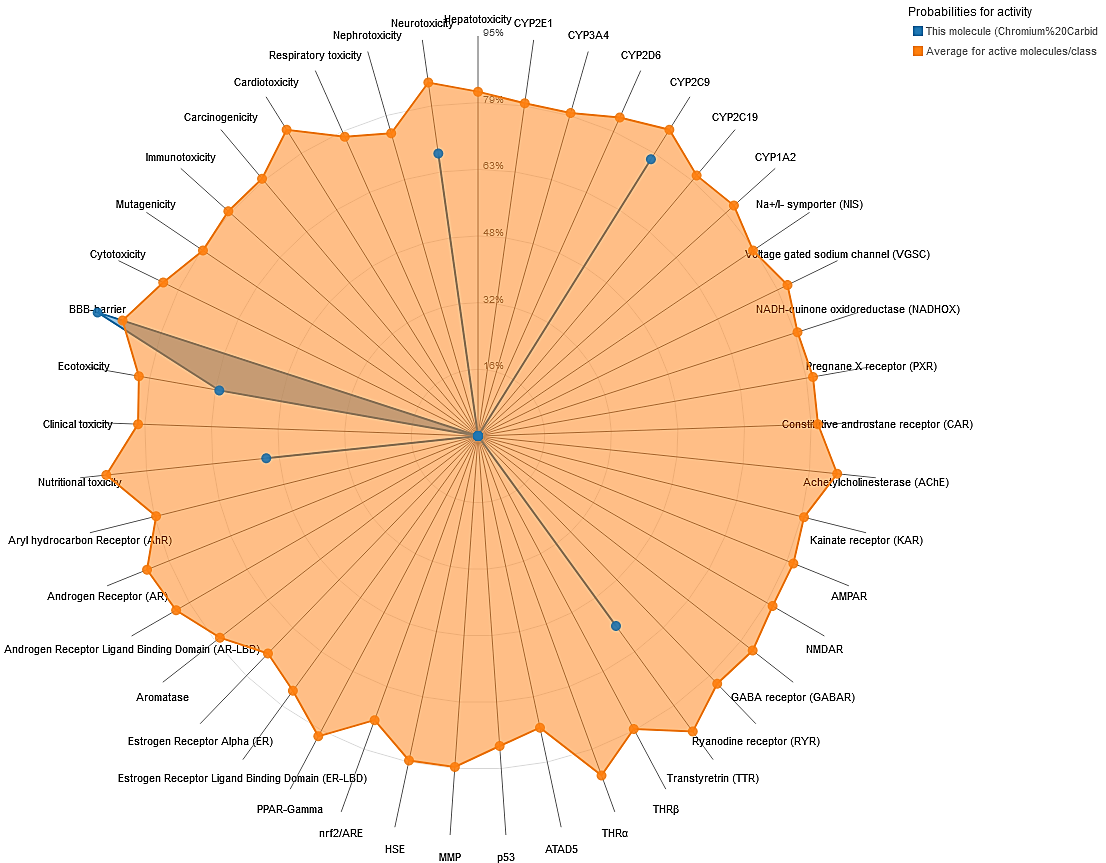


**Supplementary Figure S29:** The predicted biocompatibility/toxicity evaluation of chromium carbide (screenshot). Software by: Priyanka Banerjee, Emanuel Kemmler, Mathias Dunkel, Robert Preissner, ProTox 3.0: a webserver for the prediction of toxicity of chemicals, *Nucleic Acids Research*, Volume 52, I W1, 5 J 2024, W513–W520 <https://tox.charite.de/>.<https://doi.org/10.1093/nar/gkae303>


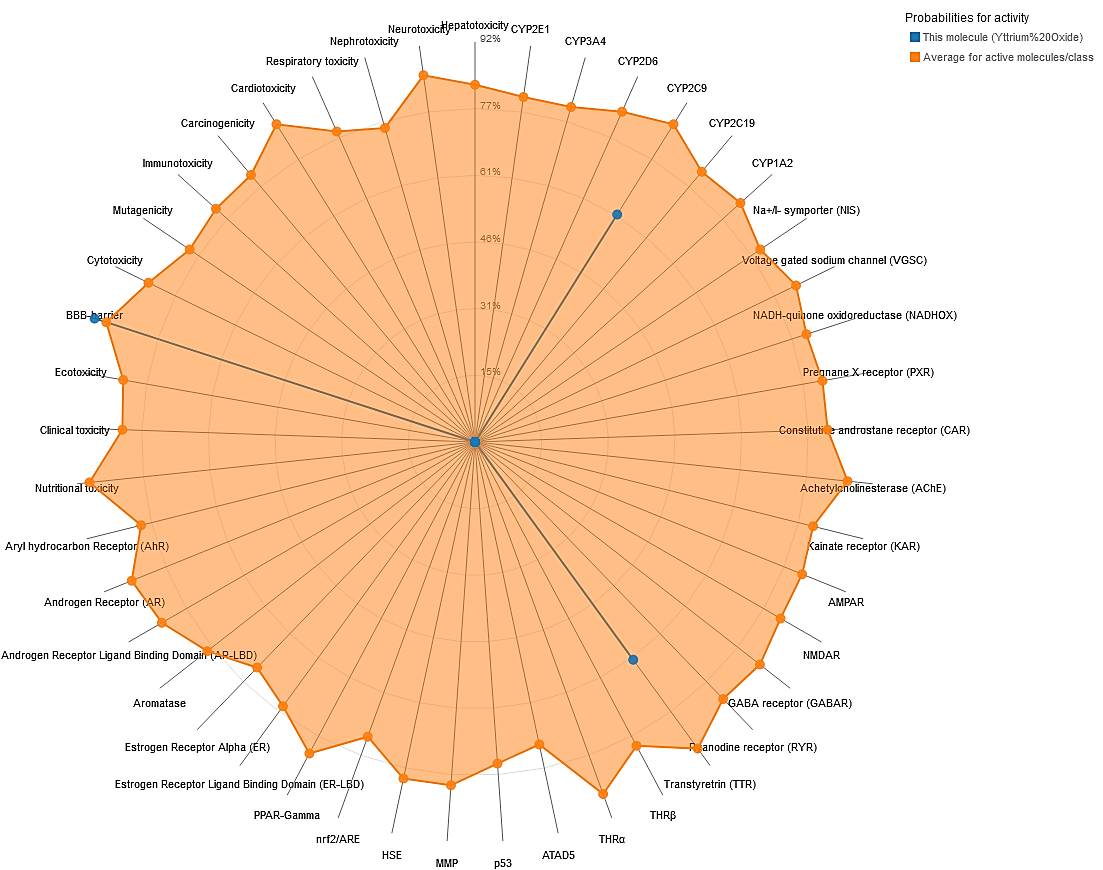


**Supplementary Figure S30:** The predicted biocompatibility/toxicity evaluation of yttrium oxide (screenshot). Software by: Priyanka Banerjee, Emanuel Kemmler, Mathias Dunkel, Robert Preissner, ProTox 3.0: a webserver for the prediction of toxicity of chemicals, *Nucleic Acids Research*, Volume 52, I W1, 5 J 2024, W513–W520 <https://tox.charite.de/>.<https://doi.org/10.1093/nar/gkae303>


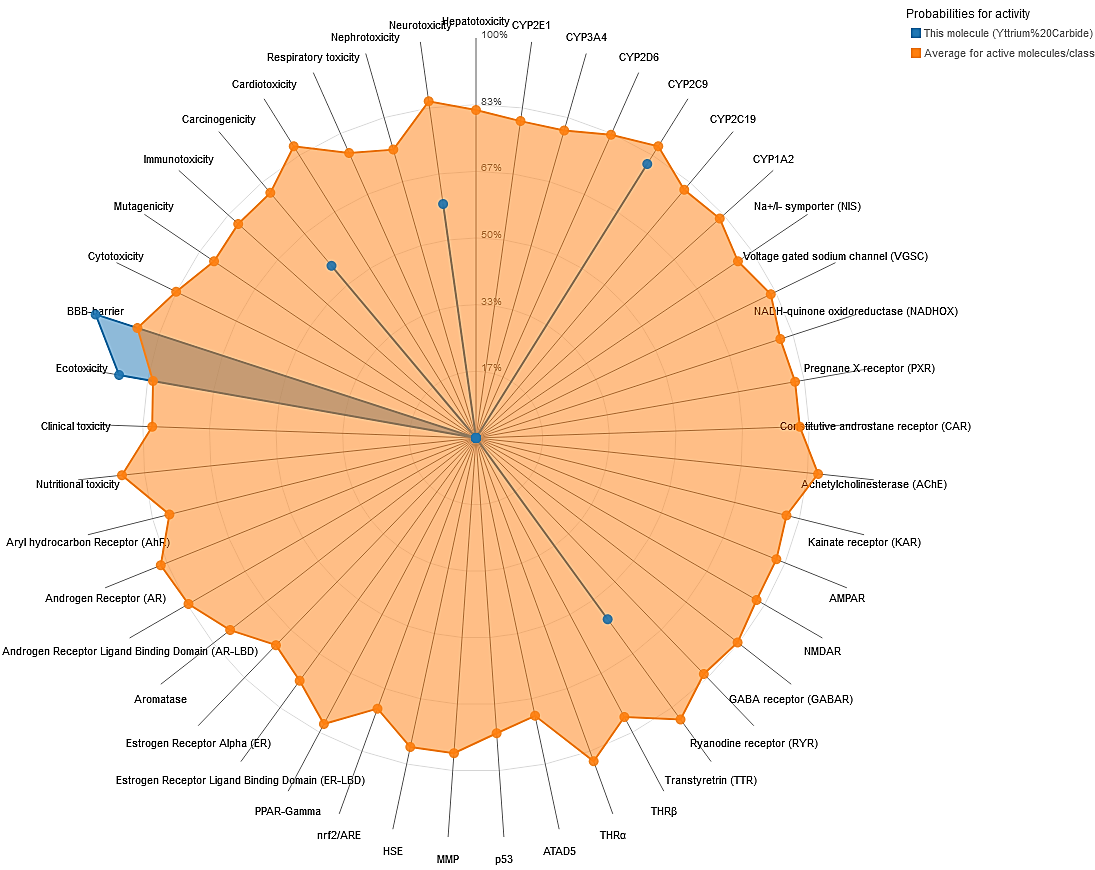


**Supplementary Figure S31:** The predicted biocompatibility/toxicity evaluation of yttrium carbide (screenshot). Software by: Priyanka Banerjee, Emanuel Kemmler, Mathias Dunkel, Robert Preissner, ProTox 3.0: a webserver for the prediction of toxicity of chemicals, *Nucleic Acids Research*, Volume 52, I W1, 5 J 2024, W513–W520 <https://tox.charite.de/>.<https://doi.org/10.1093/nar/gkae303>


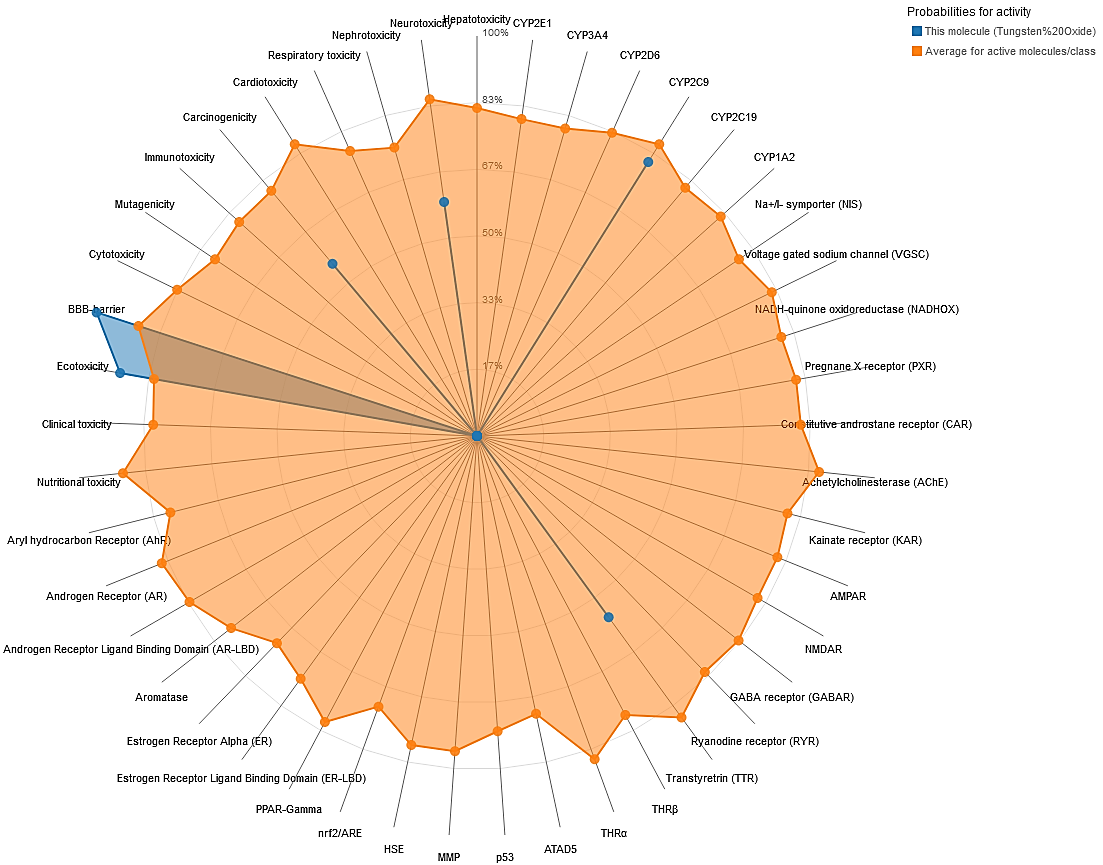


**Supplementary Figure S32:** The predicted biocompatibility/toxicity evaluation of tungsten oxide (screenshot). Software by: Priyanka Banerjee, Emanuel Kemmler, Mathias Dunkel, Robert Preissner, ProTox 3.0: a webserver for the prediction of toxicity of chemicals, *Nucleic Acids Research*, Volume 52, I W1, 5 J 2024, W513–W520 <https://tox.charite.de/>.<https://doi.org/10.1093/nar/gkae303>


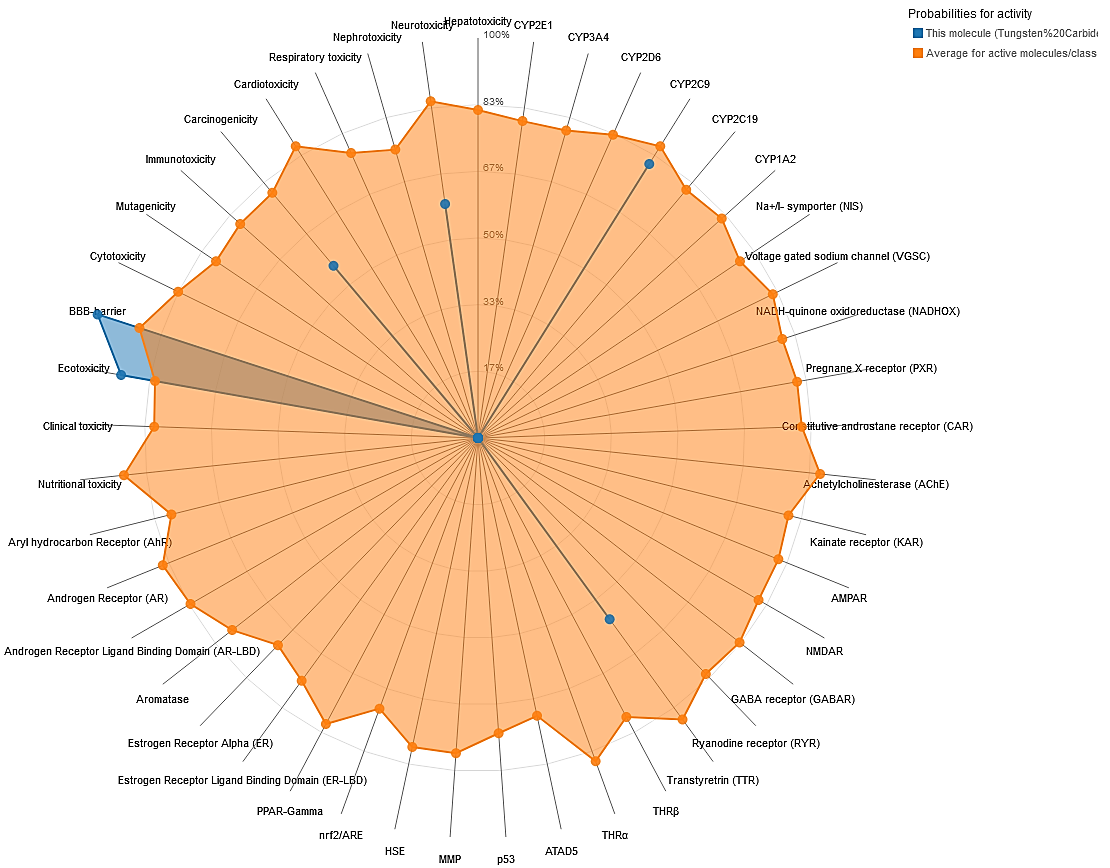


**Supplementary Figure S33:** The predicted biocompatibility/toxicity evaluation of tungsten carbide (screenshot). Software by: Priyanka Banerjee, Emanuel Kemmler, Mathias Dunkel, Robert Preissner, ProTox 3.0: a webserver for the prediction of toxicity of chemicals, *Nucleic Acids Research*, Volume 52, I W1, 5 J 2024, W513–W520 <https://tox.charite.de/>.<https://doi.org/10.1093/nar/gkae303>


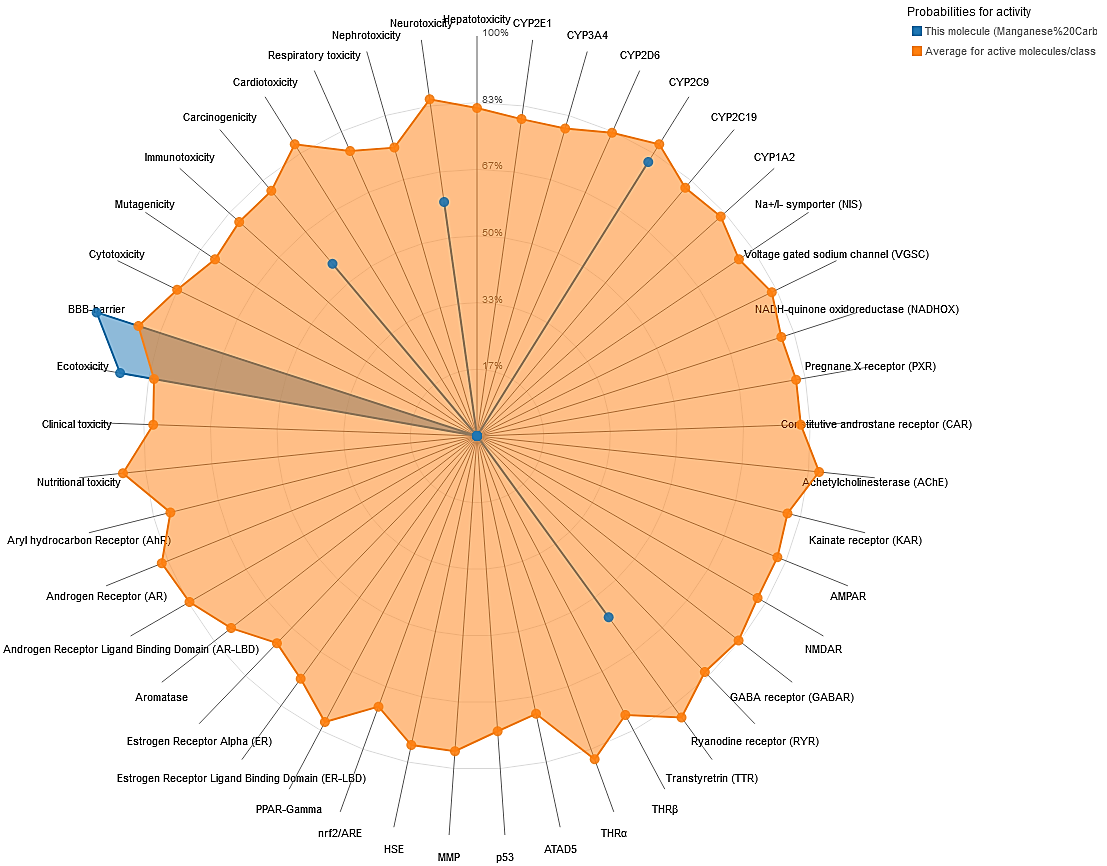


**Supplementary Figure S34:** The predicted biocompatibility/toxicity evaluation of manganese carbide (screenshot). Software by: Priyanka Banerjee, Emanuel Kemmler, Mathias Dunkel, Robert Preissner, ProTox 3.0: a webserver for the prediction of toxicity of chemicals, *Nucleic Acids Research*, Volume 52, I W1, 5 J 2024, W513–W520 <https://tox.charite.de/>.<https://doi.org/10.1093/nar/gkae303>


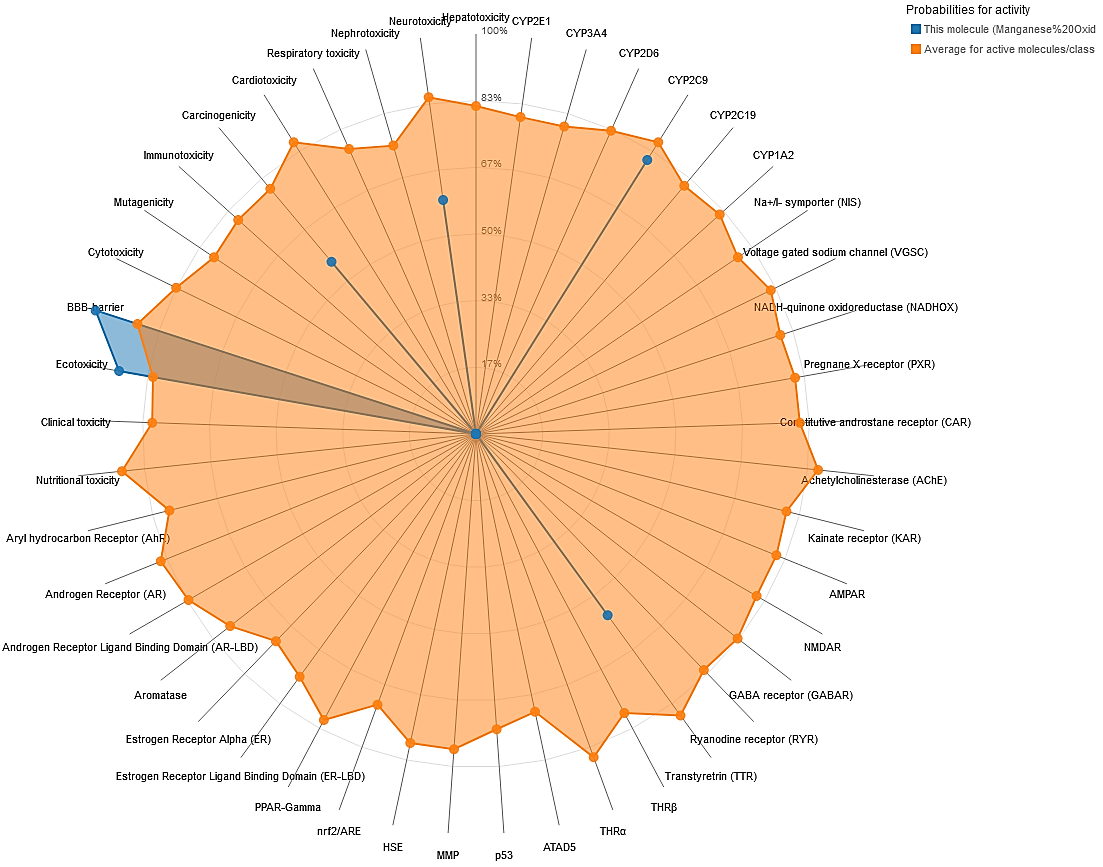


**Supplementary Figure S35:** The predicted biocompatibility/toxicity evaluation of manganese oxide (screenshot). Software by: Priyanka Banerjee, Emanuel Kemmler, Mathias Dunkel, Robert Preissner, ProTox 3.0: a webserver for the prediction of toxicity of chemicals, *Nucleic Acids Research*, Volume 52, I W1, 5 J 2024, W513–W520 <https://tox.charite.de/>.<https://doi.org/10.1093/nar/gkae303>


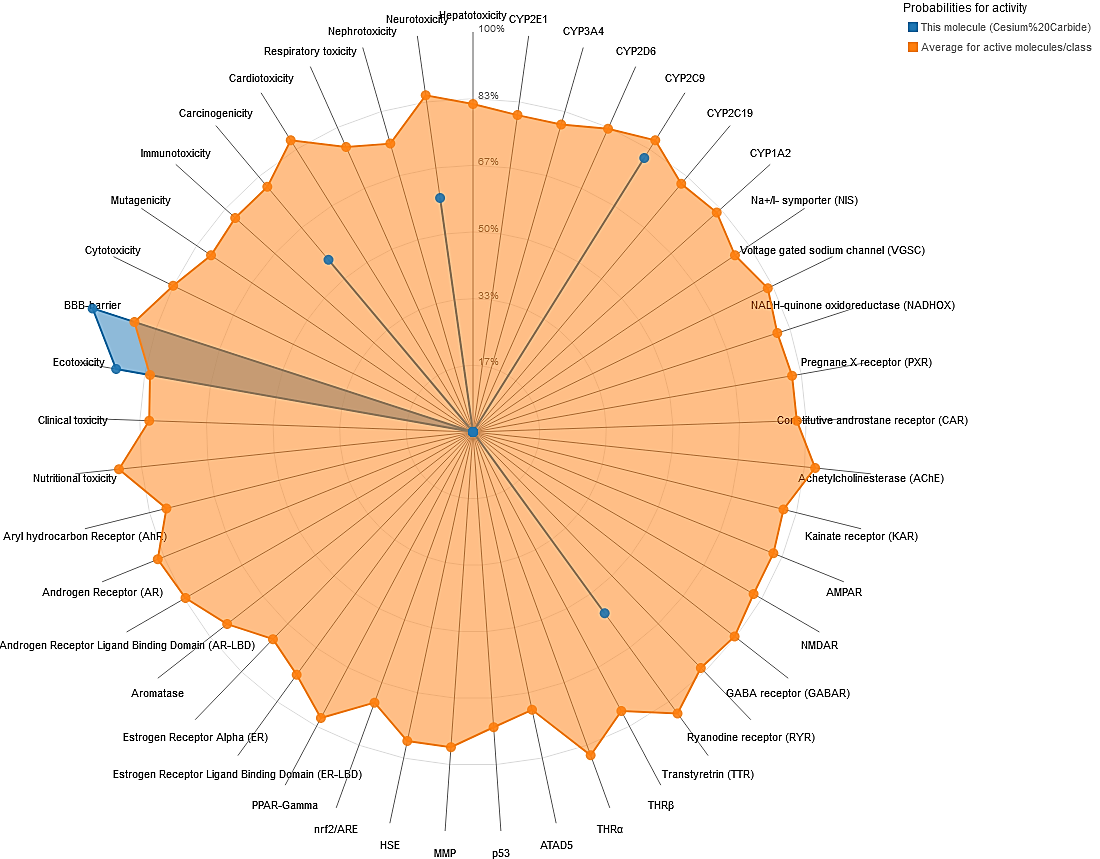


**Supplementary Figure S36:** The predicted biocompatibility/toxicity evaluation of cesium carbide (screenshot). Software by: Priyanka Banerjee, Emanuel Kemmler, Mathias Dunkel, Robert Preissner, ProTox 3.0: a webserver for the prediction of toxicity of chemicals, *Nucleic Acids Research*, Volume 52, I W1, 5 J 2024, W513–W520 <https://tox.charite.de/>.<https://doi.org/10.1093/nar/gkae303>


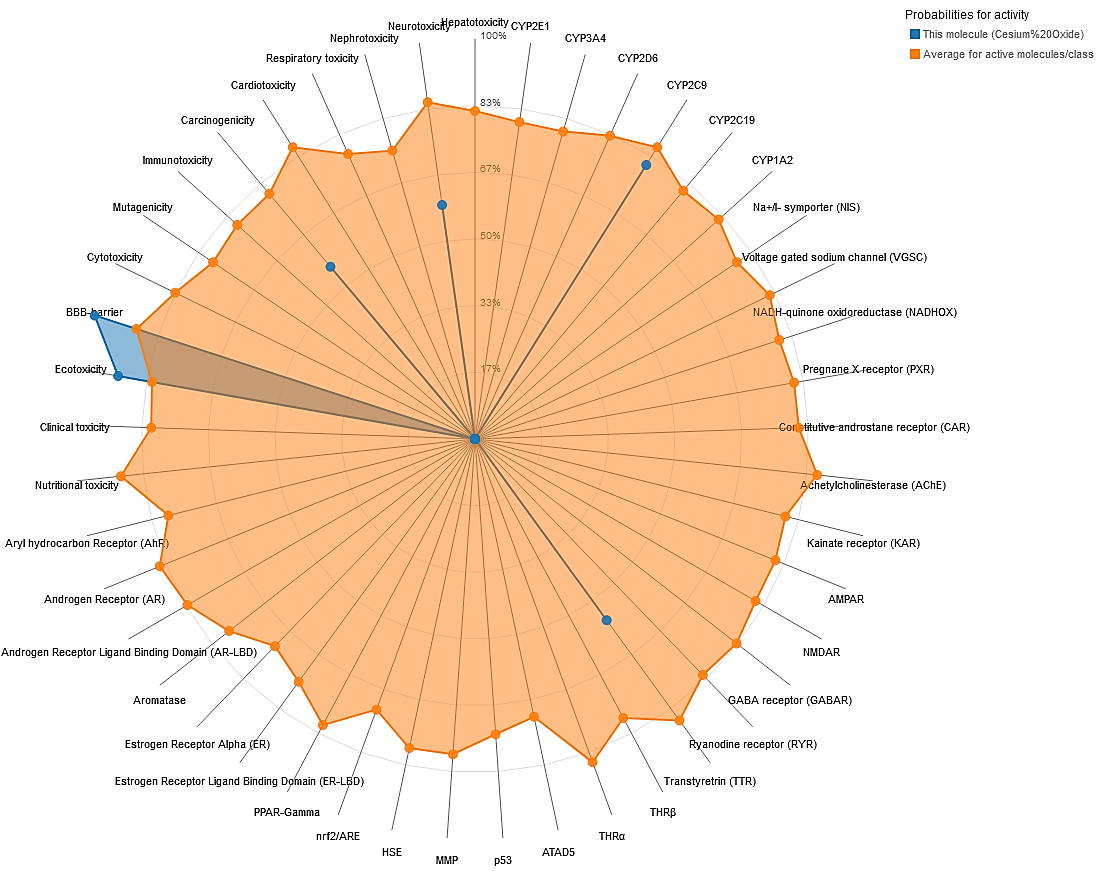


**Supplementary Figure S37:** The predicted biocompatibility/toxicity evaluation of cesium oxide (screenshot). Software by: Priyanka Banerjee, Emanuel Kemmler, Mathias Dunkel, Robert Preissner, ProTox 3.0: a webserver for the prediction of toxicity of chemicals, *Nucleic Acids Research*, Volume 52, I W1, 5 J 2024, W513–W520 <https://tox.charite.de/>.<https://doi.org/10.1093/nar/gkae303>


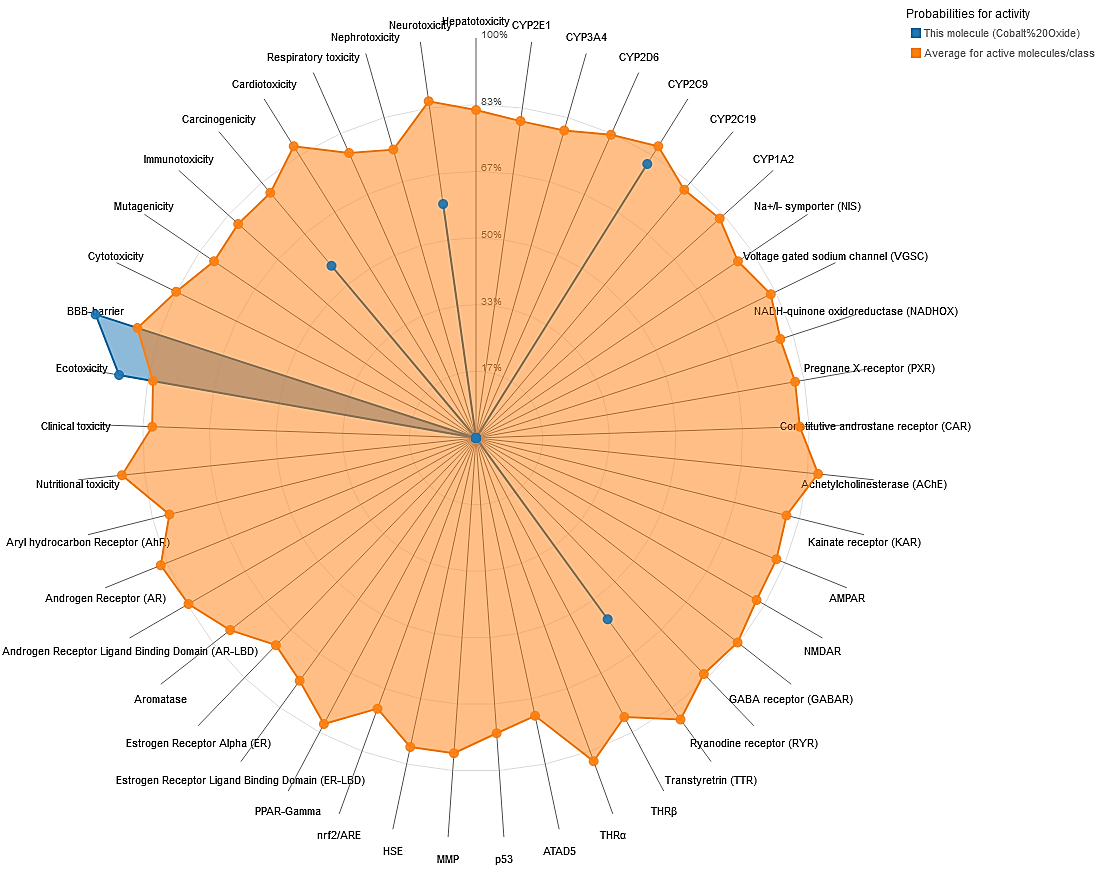


**Supplementary Figure S38:** The predicted biocompatibility/toxicity evaluation of cobalt oxide (screenshot). Software by: Priyanka Banerjee, Emanuel Kemmler, Mathias Dunkel, Robert Preissner, ProTox 3.0: a webserver for the prediction of toxicity of chemicals, *Nucleic Acids Research*, Volume 52, I W1, 5 J 2024, W513–W520 <https://tox.charite.de/>.<https://doi.org/10.1093/nar/gkae303>


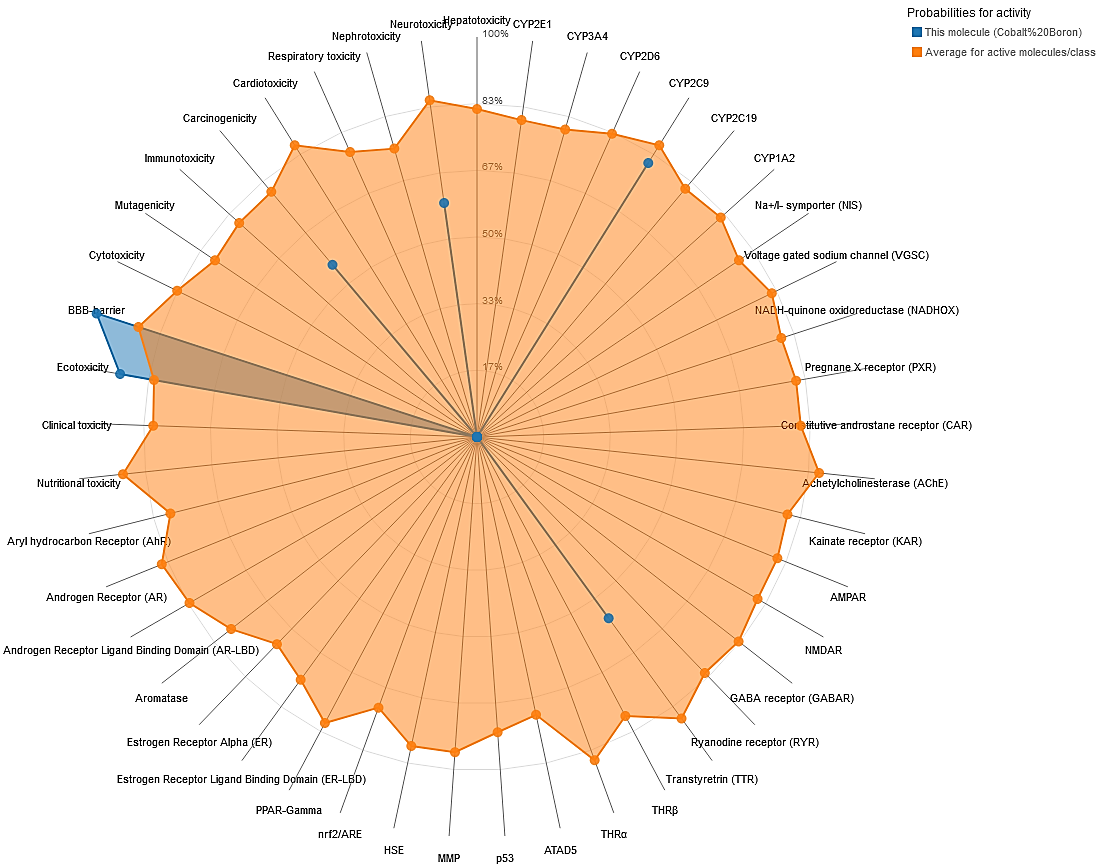


**Supplementary Figure S39:** The predicted biocompatibility/toxicity evaluation of cobalt boron (screenshot). Software by: Priyanka Banerjee, Emanuel Kemmler, Mathias Dunkel, Robert Preissner, ProTox 3.0: a webserver for the prediction of toxicity of chemicals, *Nucleic Acids Research*, Volume 52, I W1, 5 J 2024, W513–W520 <https://tox.charite.de/>.<https://doi.org/10.1093/nar/gkae303>


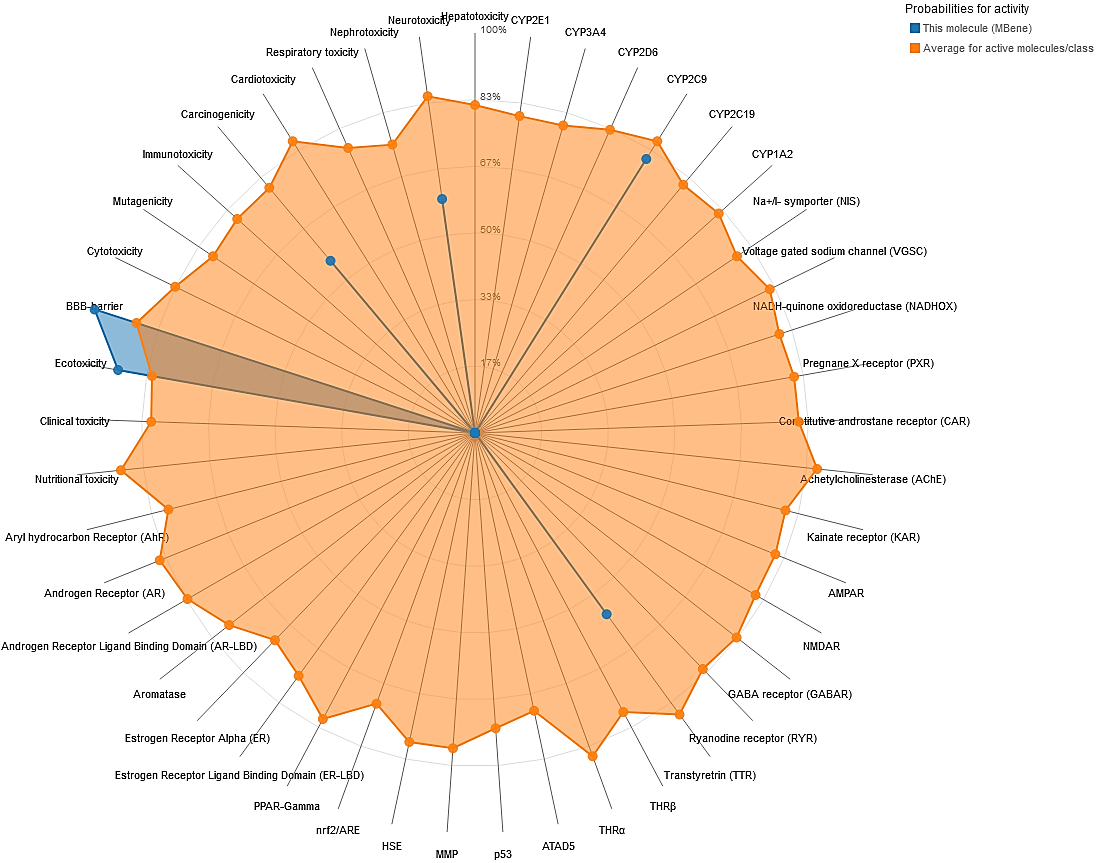


**Supplementary Figure S40:** The predicted biocompatibility/toxicity evaluation of theoretical MBene (screenshot). Software by: Priyanka Banerjee, Emanuel Kemmler, Mathias Dunkel, Robert Preissner, ProTox 3.0: a webserver for the prediction of toxicity of chemicals, *Nucleic Acids Research*, Volume 52, I W1, 5 J 2024, W513–W520 <https://tox.charite.de/>.<https://doi.org/10.1093/nar/gkae303>


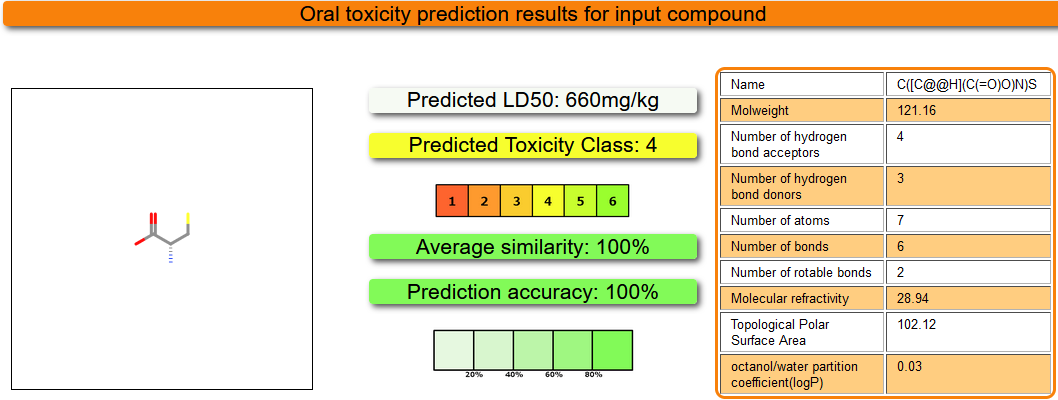


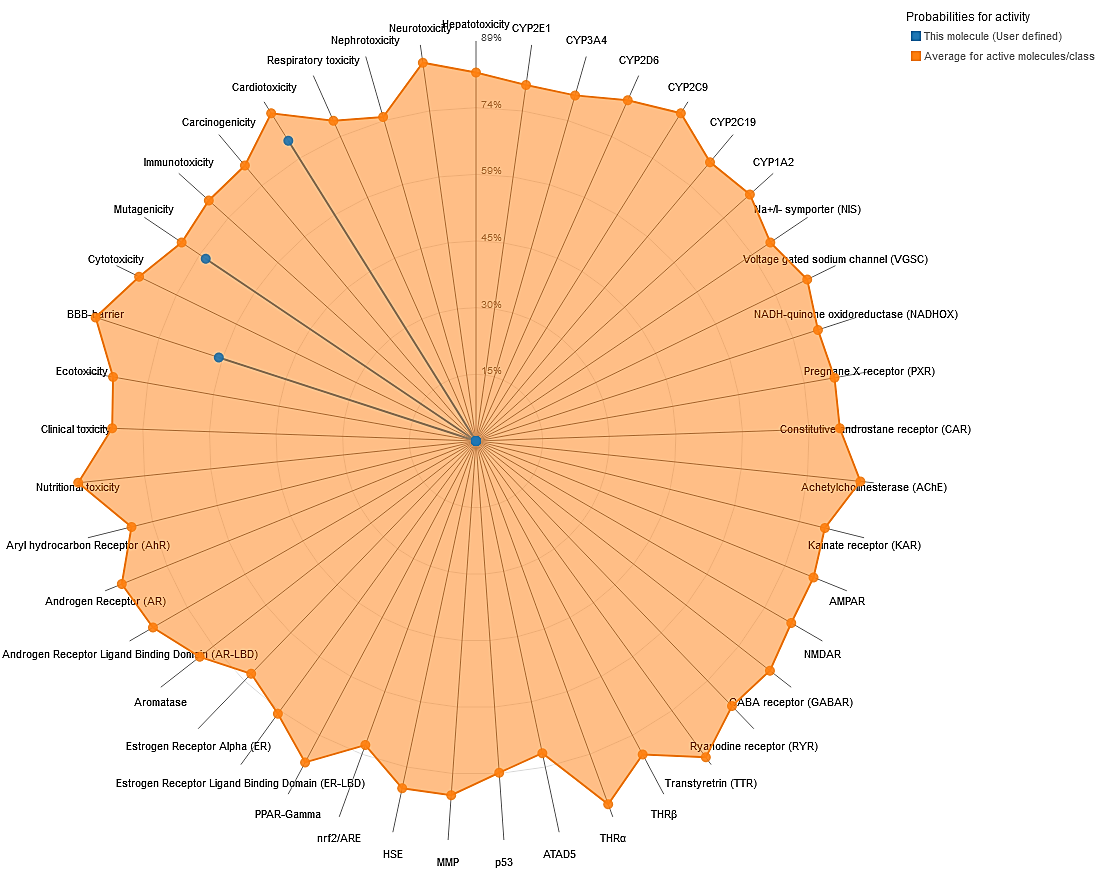


**Supplementary Figure S41:** The biocompatibility/toxicity evaluations for L-cysteine: C_3_H_7_NO_2_S, PubChem: 5862, C([C@@H](C(=O)O)N)S, <https://pubchem.ncbi.nlm.nih.gov/compound/5862>, article: DOI:10.1107/S0108270196003952. Priyanka Banerjee, Kemmler et al, ProTox 3.0: a webserver for the prediction of toxicity of chemicals, *Nucleic Acids Research*, 52, 2024, W513–W520 <https://tox.charite.de/>.<https://doi.org/10.1093/nar/gkae303>


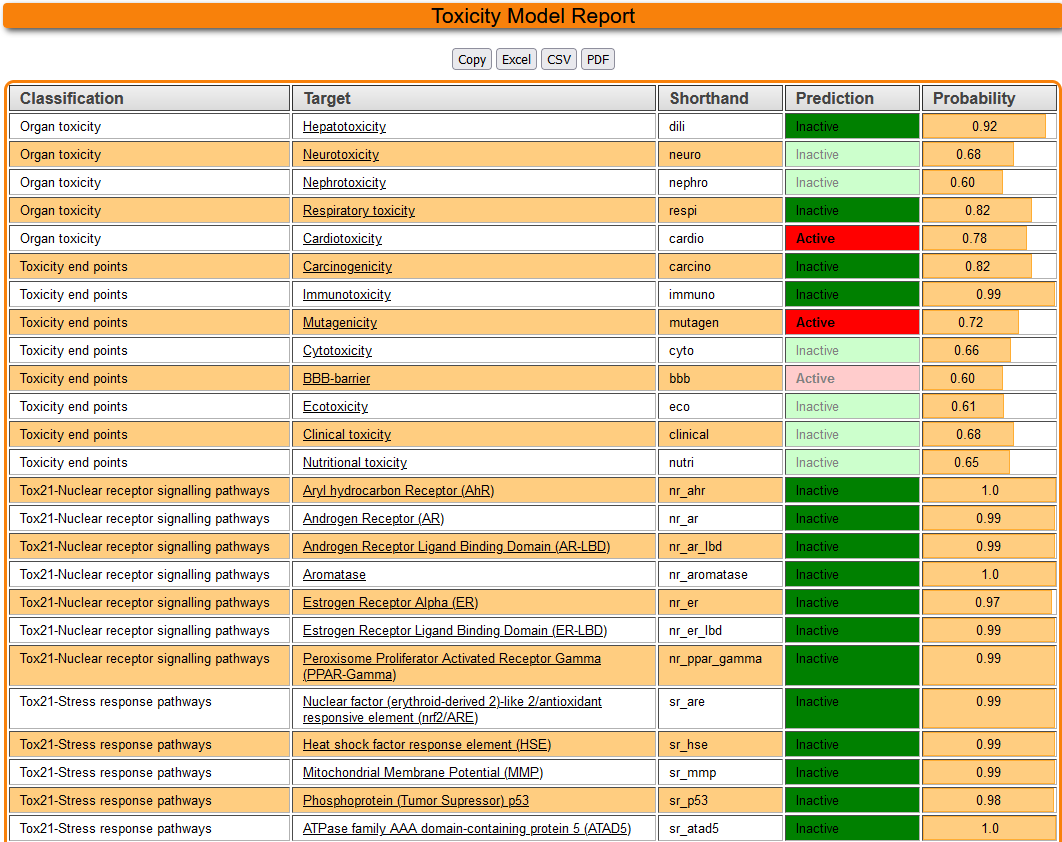


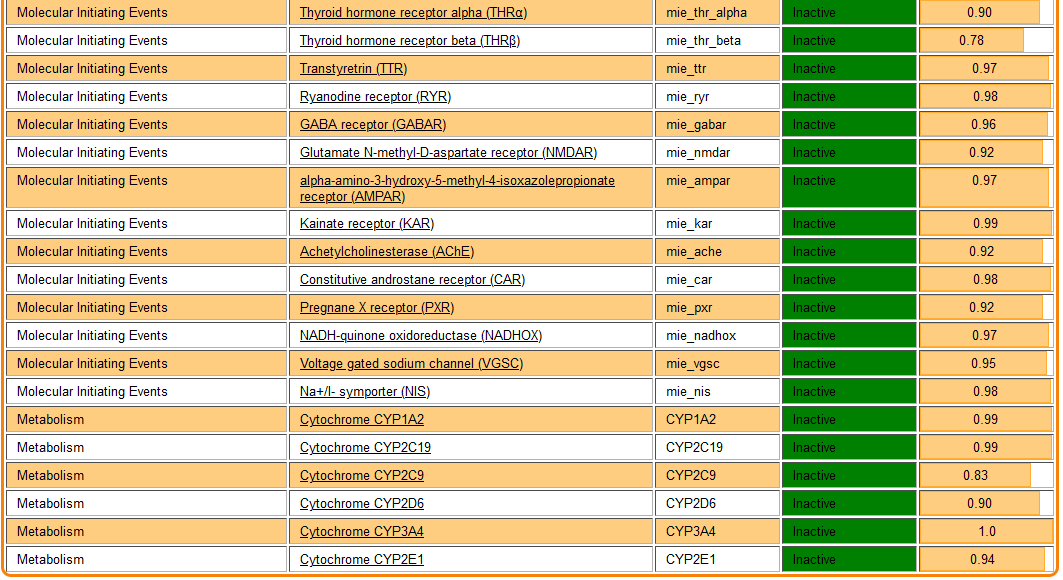


**Supplementary Figure S42:** Biocompatibility for L-cysteine: SMILES C([C@@H](C(=O)O)N)S <https://pubchem.ncbi.nlm.nih.gov/compound/5862>,: DOI:10.1107/S0108270196003952. Priyanka Banerjee, Kemmler et al, ProTox 3.0: a webserver for the prediction of toxicity of chemicals, *Nucleic Acids Research*, 52, 2024, W513–W520 <https://tox.charite.de/>.<https://doi.org/10.1093/nar/gkae303>


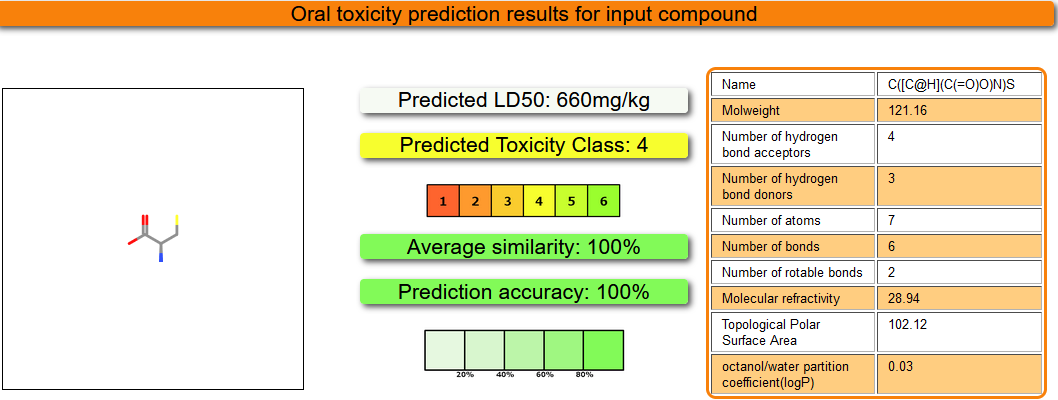


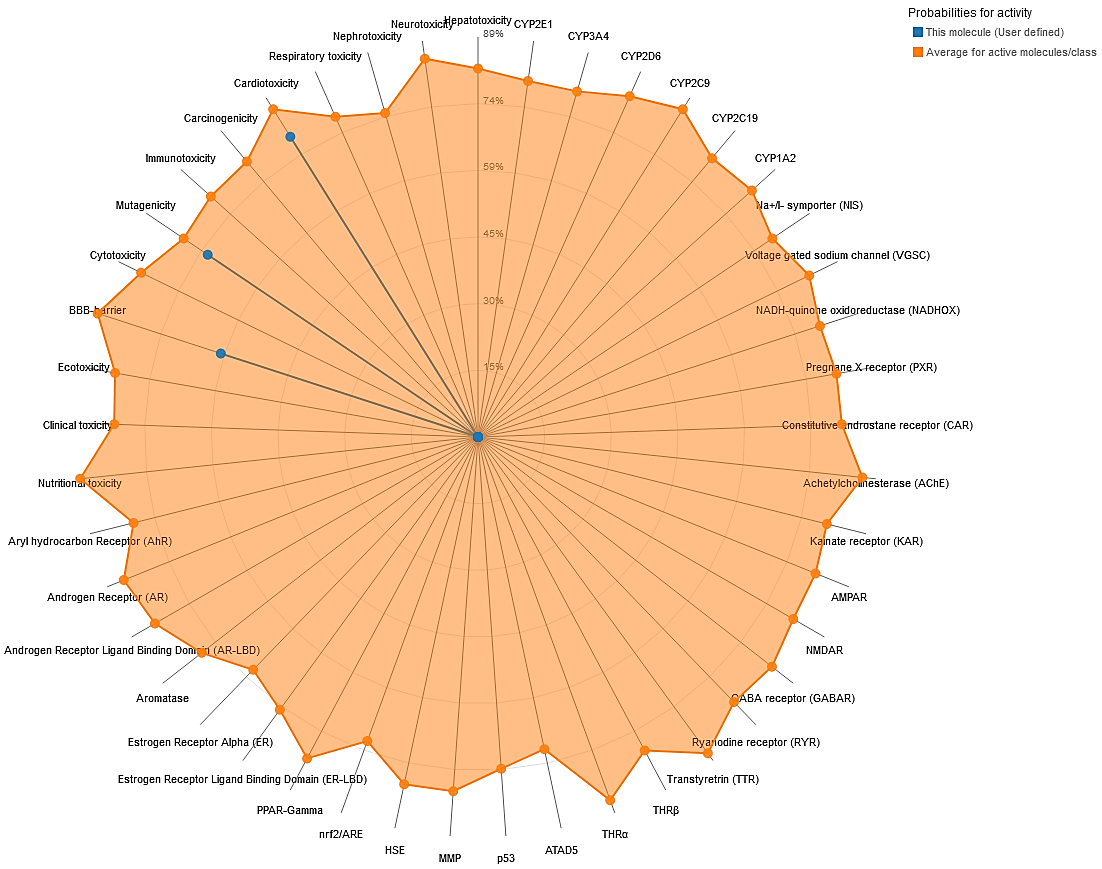


**Supplementary Figure S43:** The biocompatibility/toxicity evaluations for D-cysteine, chemical formula: C_3_H_7_NO_2_S, PubChem: 92851, SMILES: C([C@H](C(=O)O)N)S, molecular weight:121.16 g/mol, <https://pubchem.ncbi.nlm.nih.gov/compound/92851>. Priyanka Banerjee, Kemmler et al, ProTox 3.0: a webserver for the prediction of toxicity of chemicals, *Nucleic Acids Research*, 52, 2024, W513–W520 <https://tox.charite.de/>.<https://doi.org/10.1093/nar/gkae303>


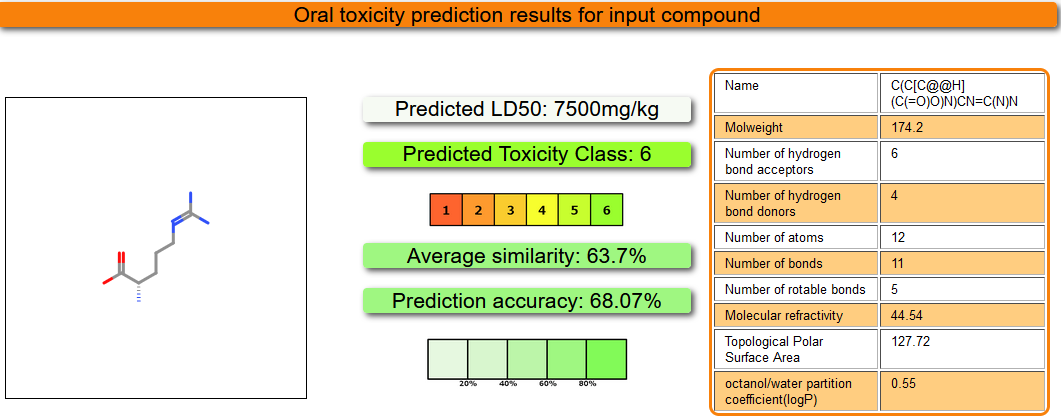


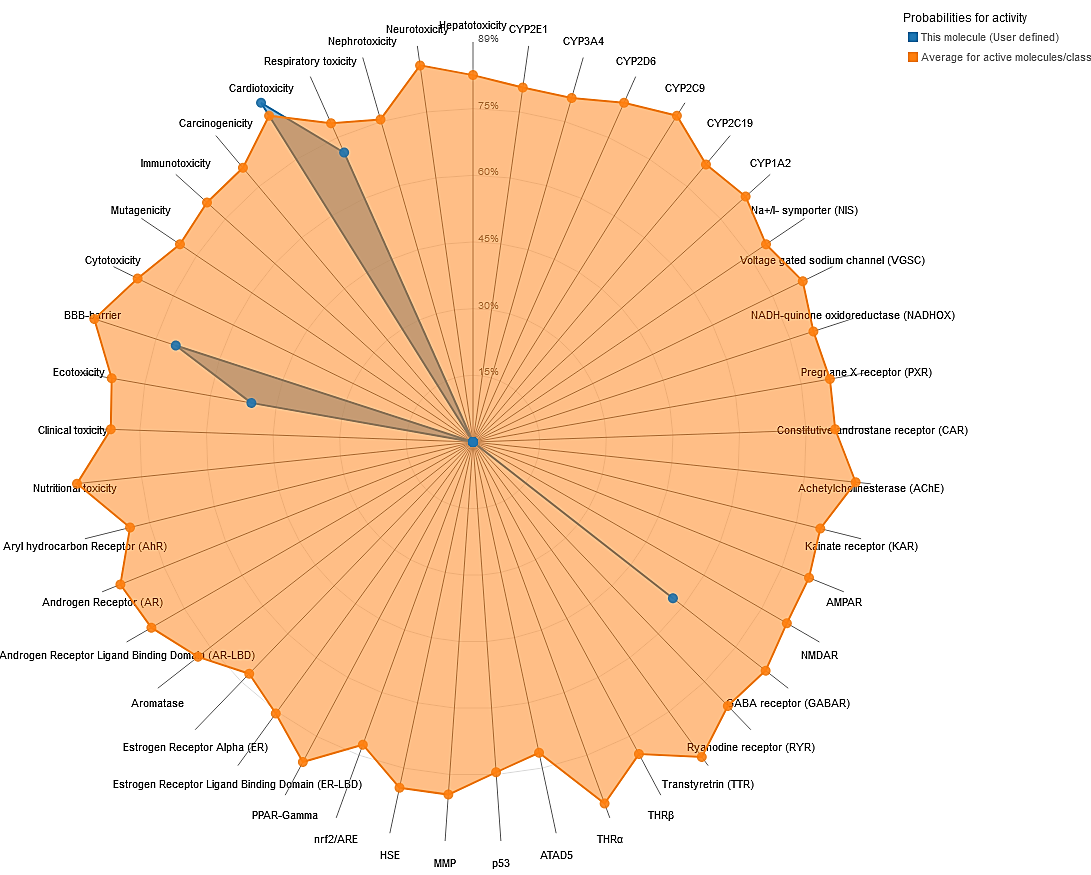


**Supplementary Figure S44:** The biocompatibility/toxicity evaluations for L-arginine: C_6_H_14_N_4_O_2_, PubChem: 6322, SMILES: C(C[C@@H](C(=O)O)N)CN=C(N)N, molecular weight: 174.20 g/mol, <https://pubchem.ncbi.nlm.nih.gov/compound/6322>. Priyanka Banerjee, Kemmler et al, ProTox 3.0: a webserver for the prediction of toxicity of chemicals, *Nucleic Acids Research*, 52, 2024, W513–W520 <https://tox.charite.de/>.<https://doi.org/10.1093/nar/gkae303>


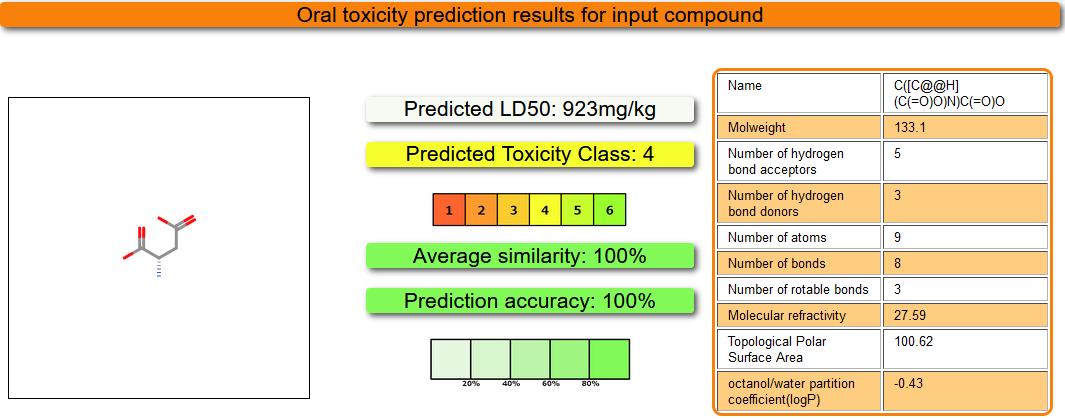


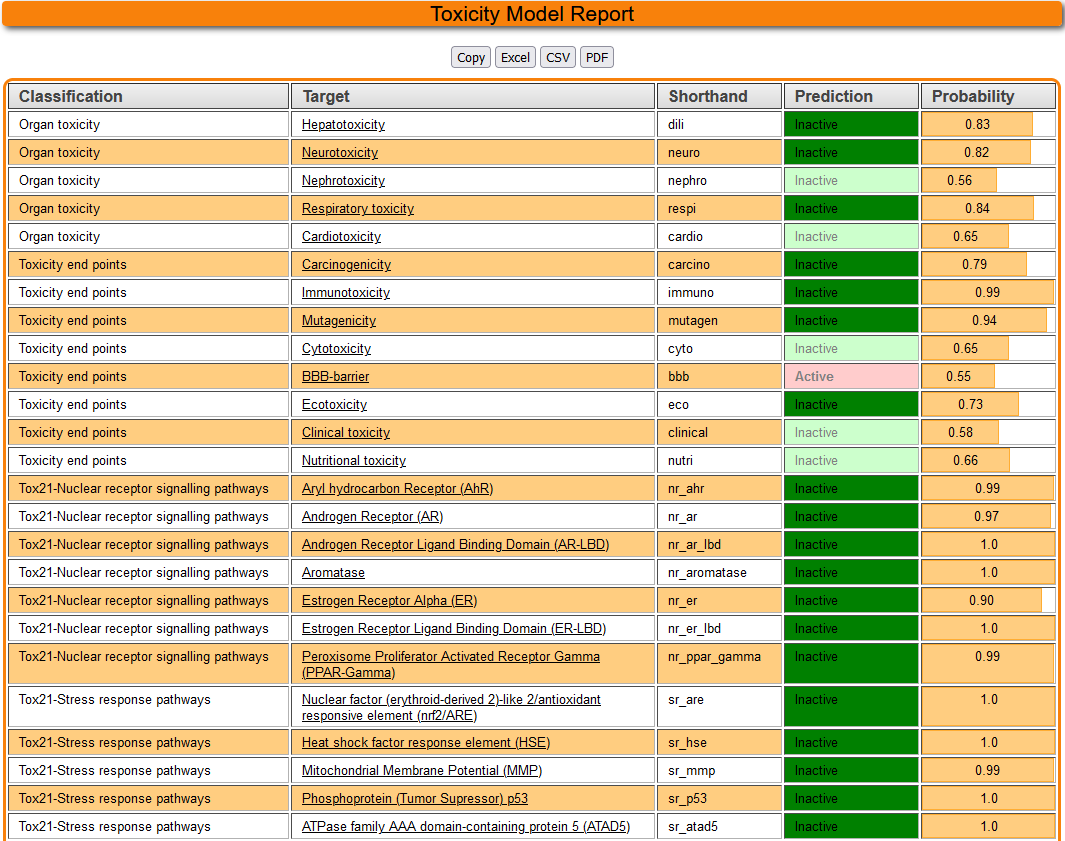


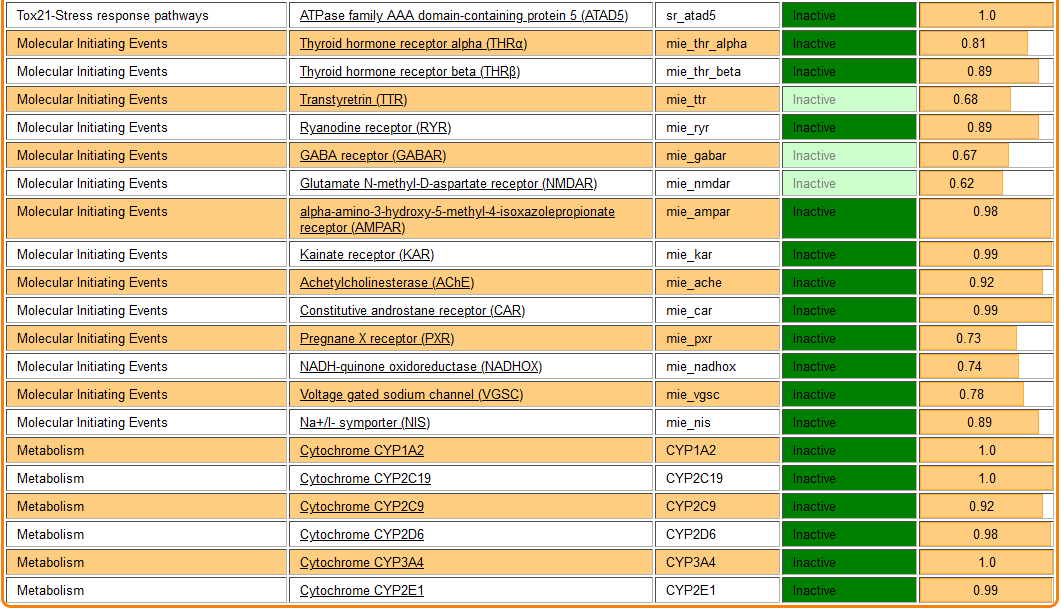


**Supplementary Figure S45:** The biocompatibility evaluations for L-aspartic acid: C_4_H_7_NO_4_, C([C@@H](C(=O)O)N)C(=O)O, <https://pubchem.ncbi.nlm.nih.gov/compound/5960>. P Banerjee, Kemmler et al ProTox 3.0 *Nucleic Acids Research* 2024, <https://tox.charite.de/>.<https://doi.org/10.1093/nar/gkae303>


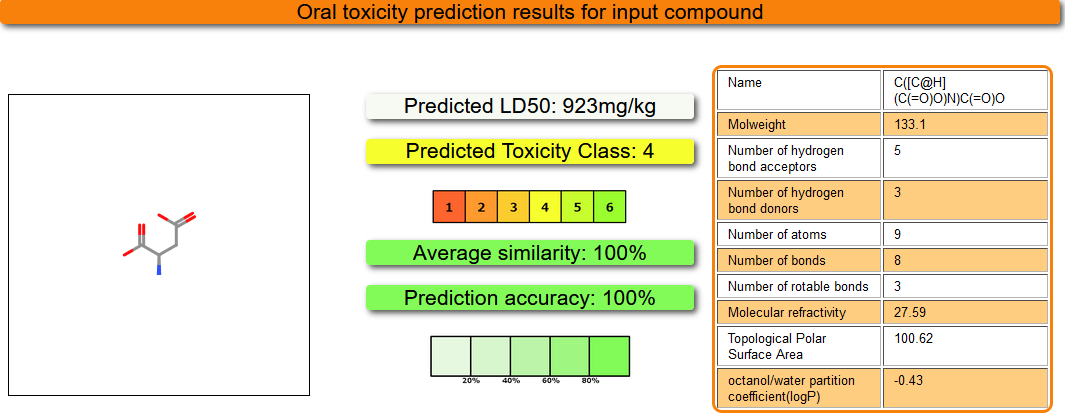


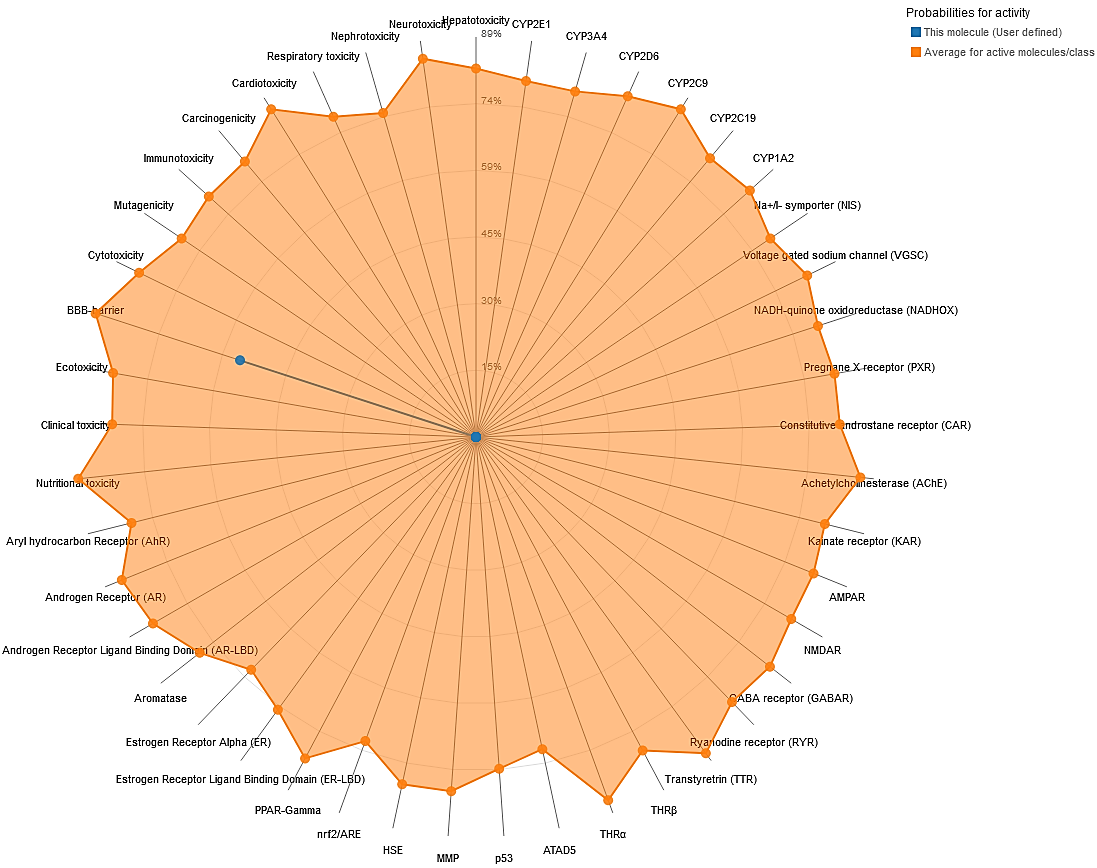


**Supplementary Figure S46:** The biocompatibility/toxicity prediction evaluations for D-aspartic acid, formula: C_4_H_7_NO_4_, PubChem: 83887, SMILES: C([C@H](C(=O)O)N)C(=O)O, molecular weight: 133.10 g/mol, <https://pubchem.ncbi.nlm.nih.gov/compound/83887>. Priyanka Banerjee, Kemmler et al, ProTox 3.0: a webserver for the prediction of toxicity of chemicals, *Nucleic Acids Research*, 52, 2024, W513–W520 <https://tox.charite.de/>.<https://doi.org/10.1093/nar/gkae303>


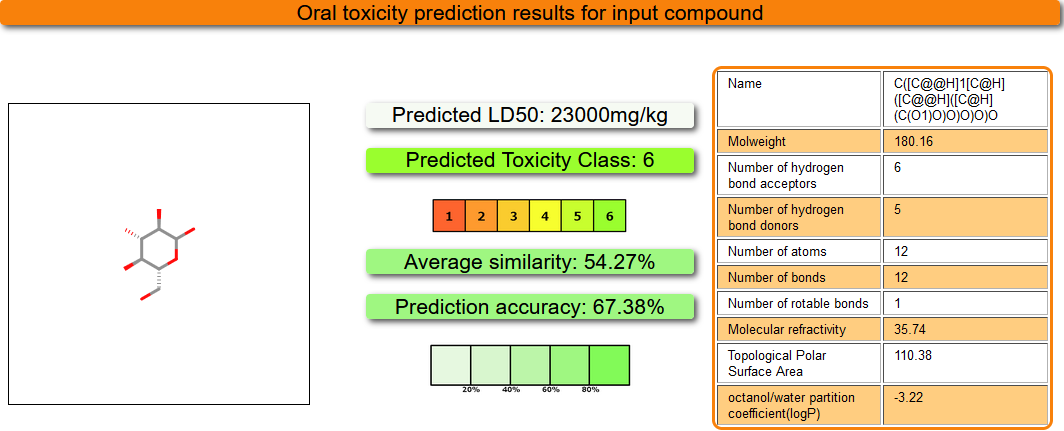


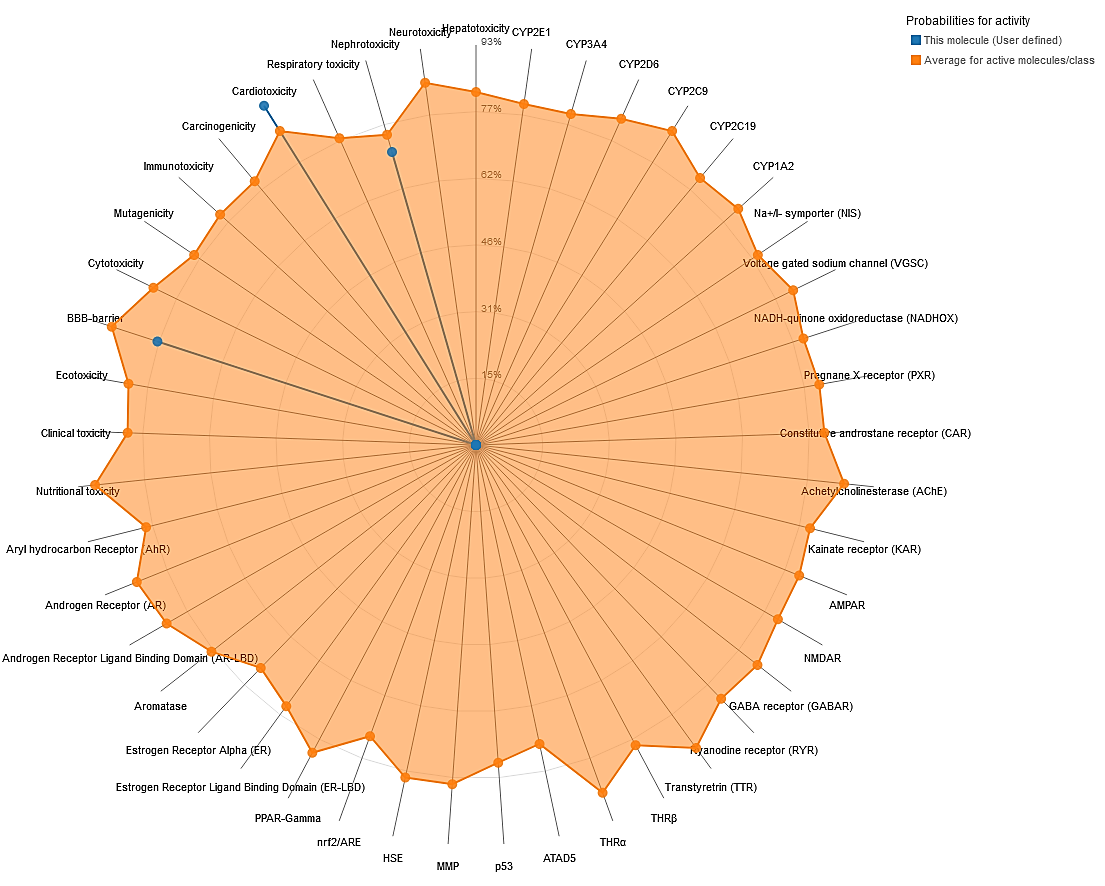


**Supplementary Figure S47:** The biocompatibility/toxicity prediction evaluations for D-glucose: chemical: C_6_H_12_O_6_, PubChem: 5793: C([C@@H]1[C@H]([C@@H]([C@H](C(O1)O)O)O)O)O, molecular weight: 180.16 g/mol, <https://pubchem.ncbi.nlm.nih.gov/compound/5793>. Priyanka Banerjee, Kemmler et al, ProTox 3.0: a webserver for the prediction of toxicity of chemicals, *Nucleic Acids Research*, 52, 2024, W513–W520 <https://tox.charite.de/>.<https://doi.org/10.1093/nar/gkae303>


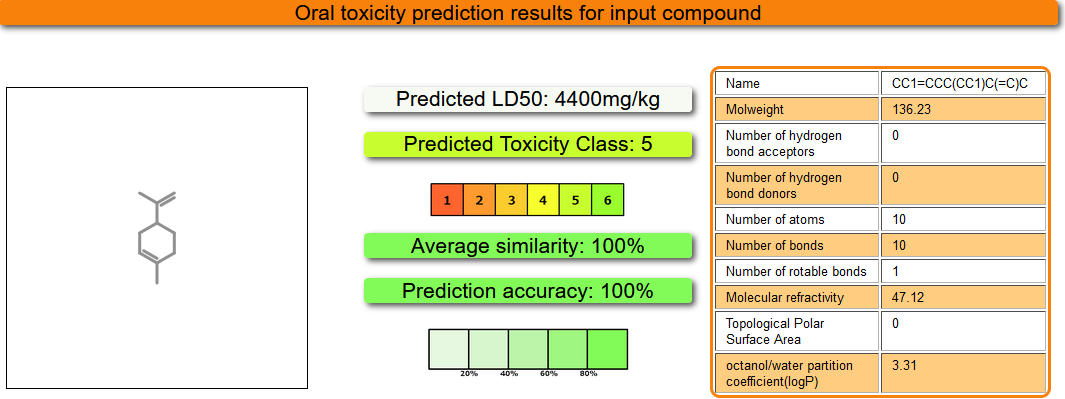


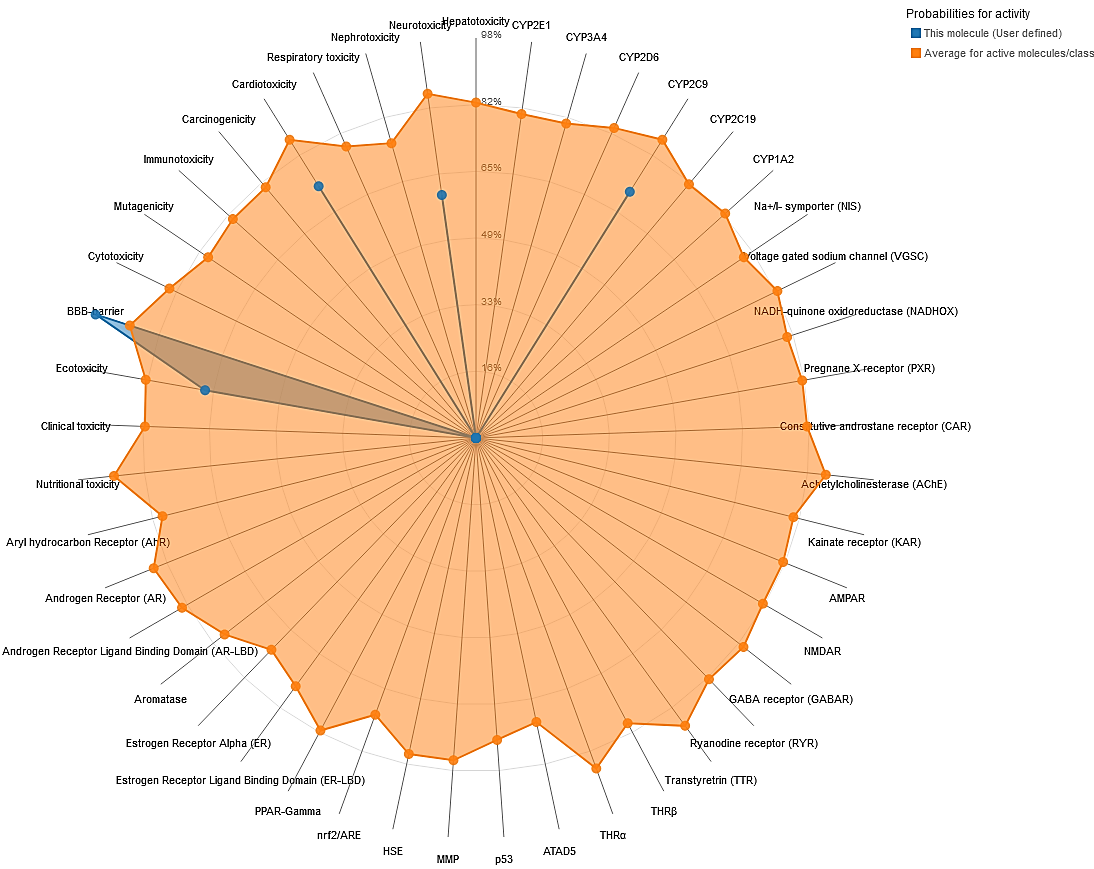


**Supplementary Figure S48:** The biocompatibility/toxicity prediction evaluations for Limonene, chemical formula: C_10_H_16_, PubChem ID: 22311: CC1=CCC(CC1)C(=C)C molecular weight: 136.23 g/mol, <https://pubchem.ncbi.nlm.nih.gov/compound/22311>. Priyanka Banerjee, Kemmler et al, ProTox 3.0: a webserver for the prediction of toxicity of chemicals, *Nucleic Acids Research*, 52, 2024, W513–W520 <https://tox.charite.de/>.<https://doi.org/10.1093/nar/gkae303>


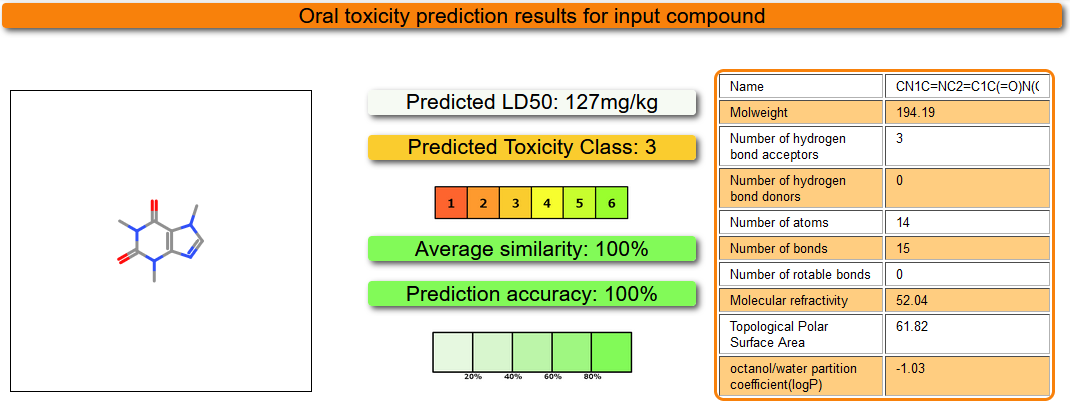


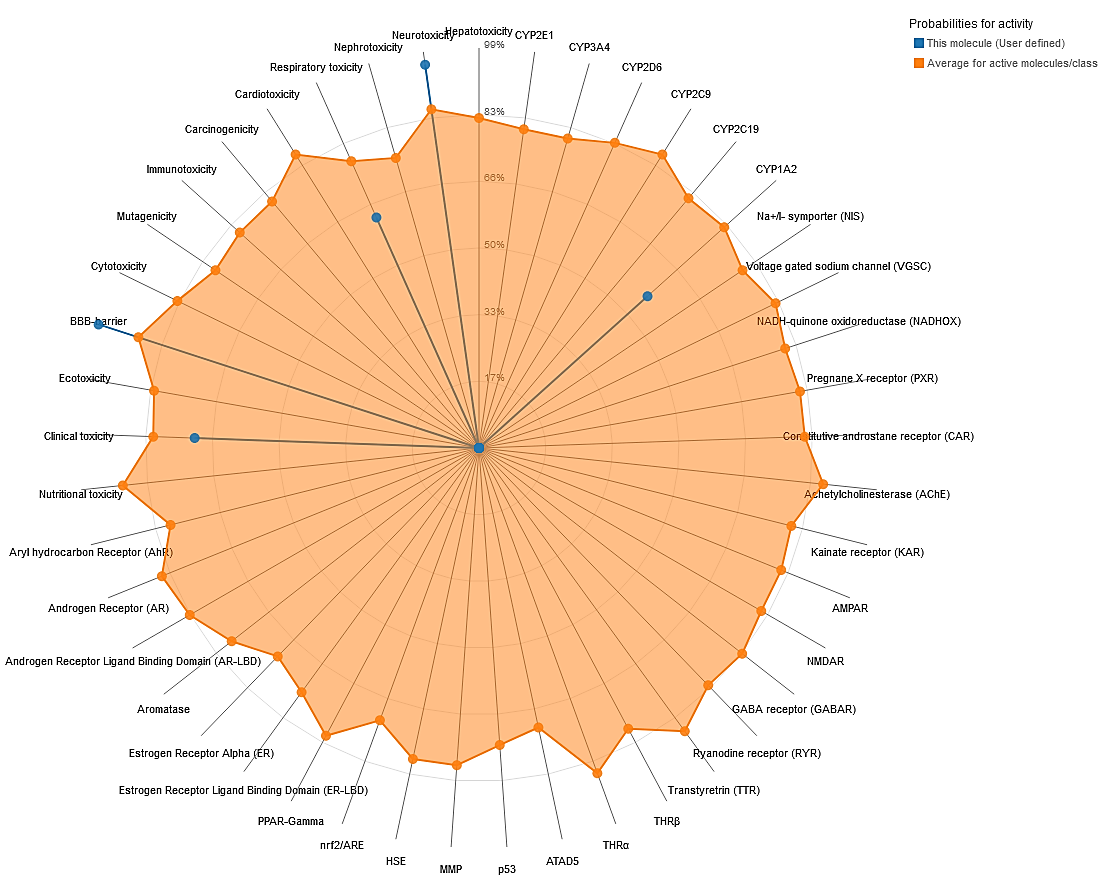


**Supplementary Figure S49:** The biocompatibility/toxicity prediction evaluations for Caffeine, formula: C_8_H_10_N_4_O_2_, PubChem: 2519: SMILES CN1C=NC2=C1C(=O)N(C(=O)N2C)C molecular weight: 194.19 g/mol, <https://pubchem.ncbi.nlm.nih.gov/compound/2519>. Priyanka Banerjee, Kemmler et al, ProTox 3.0: a webserver for the prediction of toxicity of chemicals, *Nucleic Acids Research*, 52, 2024, W513–W520 <https://tox.charite.de/>.<https://doi.org/10.1093/nar/gkae303>


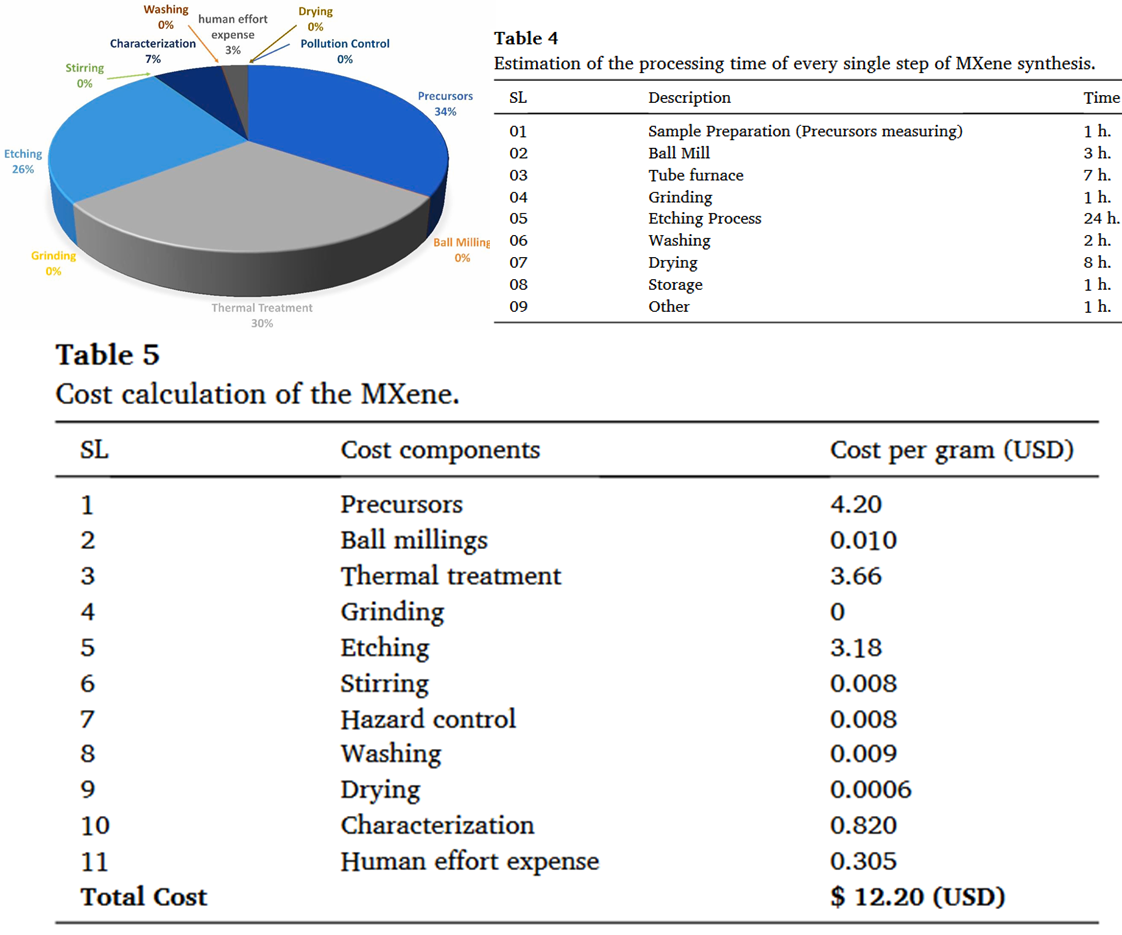


**Supplementary Figure S50:** Representative illustration of recently-reported cost consideration of MXenes towards their price reduction and large-scale production. *Panels are adapted and merged from references:* Zaed, Md Abu, Kim Han Tan, Norulsamani Abdullah, R. Saidur, Adarsh Kumar Pandey, and Ahmed Mortuza Saleque. "Cost analysis of MXene for low-cost production, and pinpointing of its economic footprint." *Open Ceramics* 17 (2024): 100526. <https://doi.org/10.1016/j.oceram.2023.100526> And Zaed, M.A., Tan, K.H., Saidur, R. *et al.* Invited viewpoint: pathways to low-cost MXene synthesis. *J Mater Sci* **59**, 7575–7594 (2024). <https://doi.org/10.1007/s10853-024-09666-6>.
